# p300‐Mediated H3K18 Lactylation Drives Radiation‐Induced Pulmonary Fibrosis via VIRMA‐Dependent m6A Modification of GATA3

**DOI:** 10.1002/advs.77328

**Published:** 2026-08-24

**Authors:** Junxuan Yi, Mingwei Wang, Duo Yu, Mingqi Zhao, Xinyan Wang, Shiqi Li, Xiaowen Han, Wei Wei, Shunzi Jin

**Affiliations:** ^1^ NHC Key Laboratory of Radiobiology School of Public Health Jilin University Changchun Jilin China; ^2^ Department of Radiotherapy China‐Japan Union Hospital of Jilin University Changchun Jilin China; ^3^ NHC Key Laboratory of Nuclear Technology Medical Transformation Mianyang Central Hospital Mianyang China; ^4^ Department of Radiation Oncology, Senior Department of Oncology the Fifth Medical Center of PLA General Hospital Beijing China

**Keywords:** GATA3, H3K18la, m6A, Radiation‐induced pulmonary fibrosis, VIRMA

## Abstract

Radiation‐induced pulmonary fibrosis, a devastating thoracic radiotherapy sequela, features aberrant ECM deposition and irreversible loss of functional lung architecture. However, the exact molecular mechanisms governing AECII dysfunction and fate transition during RIPF remain poorly understood. Here, we identify a novel metabolic‐epigenetic‐epitranscriptomic cascade that orchestrates the pro‐fibrotic fate of AECIIs. We demonstrate that radiation exposure triggers a glycolytic shift in AECIIs, resulting in pathological intracellular lactate accumulation. This metabolic stress is directly coupled to chromatin remodeling via p300‐mediated H3K18la at the *VIRMA* promoter, driving its robust transcriptional activation. Consequently, elevated VIRMA promotes a pro‐fibrotic epitranscriptomic landscape by increasing m6A enrichment on the *GATA3* mRNA. Recognition of this modification by the m6A reader YTHDF1 extends *GATA3* mRNA half‐life. The ensuing GATA3 accumulation initiates EMT and exacerbates ECM deposition. Strikingly, utilizing AECII‐specific Virma conditional knockout mice, we confirmed the essential role of this axis in vivo; genetic ablation of Virma preserved alveolar integrity and conferred resistance to radiation‐induced fibrogenesis. Furthermore, pharmacological inhibition of the H3K18la writer p300 using C646 abrogated the fibrotic phenotype. Collectively, our findings elucidate how radiation‐induced metabolic reprogramming is durably inscribed into the epigenome to dictate cell fate, offering a therapeutic rationale for targeting the H3K18la/VIRMA/GATA3 axis in RIPF.

## Graphical abstract

Radiation triggers glycolytic reprogramming in alveolar epithelial type II cells, causing pathological lactate accumulation. This metabolic stress drives p300‐mediated H3K18 lactylation at the VIRMA promoter, activating transcription. Elevated VIRMA enriches m6A on GATA3 mRNA, which YTHDF1 stabilizes, ultimately driving epithelial–mesenchymal transition, extracellular matrix deposition, and radiation‐induced pulmonary fibrosis.

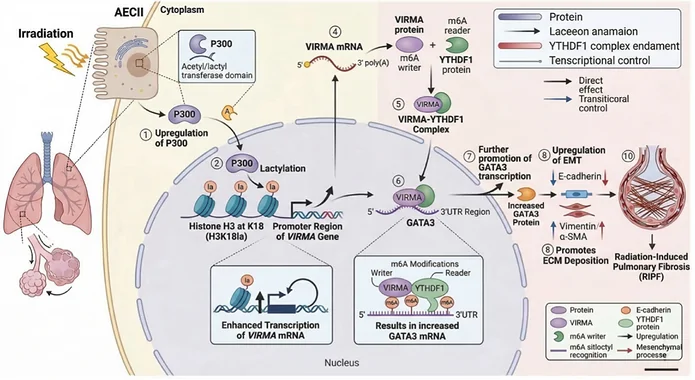

## 1 Introduction

Radiation‐induced pulmonary fibrosis (RIPF) is a devastating, permanent consequence of thoracic radiation [1, 2], characterized by progressive alveolar destruction and massive extracellular matrix (ECM) deposition [3, 4]. As the principal progenitors maintaining pulmonary homeostasis, type II alveolar epithelial cells (AECIIs) paradoxically exacerbate RIPF by undergoing epithelial‐mesenchymal transition (EMT) [5], acting as a major source of lesional fibroblasts. Historically, the canonical TGF‐β/Smad cascade has been viewed as the primary driver of fibrogenesis [6, 7]. However, this paradigm fails to elucidate a critical “metabolic‐epigenetic” paradox: how a transient ionizing radiation insult triggers a self‐perpetuating pro‐fibrotic program that persists long after the initial exposure. Consequently, decoding the molecular mechanisms that lock AECIIs into this sustained pathological memory is imperative for discovering effective therapeutic targets.

Metabolic reprogramming, a hallmark of the Warburg effect [8], is increasingly recognized as a primary cause of chronic fibroproliferative disorders [9]. As a hypoxic waste product, lactate has emerged as a strong signaling molecule that directly regulates cellular fate [10, 11]. Upon alveolar injury, AECIIs undergo a “Warburg‐like” glycolytic shift, resulting in pathological intracellular lactate accumulation [12]. This metabolic perturbation extends beyond bioenergetics to directly remodel the epigenome via histone lactylation (Kla) [13, 14]. Lactate, as a substrate for “writer” enzymes such as p300, enables changes like H3K18 lactylation (H3K18la), effectively translating transient metabolic fluctuations into stable epigenetic memory [15, 16]. Emerging evidence underscores the pathological relevance of this metabolic‐epigenetic crosstalk. In models of bleomycin‐ and arsenic‐induced lung injury, p300‐mediated lactylation at pro‐fibrotic gene promoters, together with upregulation of RNA readers such as YTHDF1, drives fibrogenesis [17, 18]. Despite these insights, the specific molecular mechanisms sustaining RIPF remain elusive. Whether ionizing radiation initiates a similar metabolically driven epigenetic mechanism, and whether radiation‐induced lactylation serves as a key switch for a self‐perpetuating pro‐fibrotic program, remains an open question.

Beyond the epigenome, the epitranscriptome further dictates transcript fate, driving dynamic cellular adaptations to stress. As the predominant internal mRNA modification, N6‐methyladenosine (m6A) is dynamically regulated by the VIRMA‐scaffolded methyltransferase complex [19, 20, 21]. It is unclear how this epitranscriptomic machinery stabilizes pro‐fibrotic networks during radiation injury, even while it controls particular m6A deposition and frequently acts in concert with “readers” like YTHDF1 to improve transcript stability [22, 23]. We previously identified the transcription factor GATA3 as a critical driver of RIPF [24]. Yet, the upstream post‐transcriptional mechanisms sustaining aberrant GATA3 expression following radiation exposure are unknown. Bridging this gap, we investigate whether ionizing radiation regulates the VIRMA/YTHDF1 axis to hypermethylate and stabilize *GATA3* mRNA.

Here, we hypothesize that radiation‐induced metabolic shifts are integrated into the epitranscriptome via a p300‐mediated lactylation cascade. We demonstrate that p300, functioning as a histone lactyltransferase, catalyzes H3K18la at the *VIRMA* promoter, activating its transcription in a lactate‐dependent manner. This upregulation facilitates the recruitment of YTHDF1 to the *GATA3* 3′ UTR, where m6A modification acts as a post‐transcriptional stabilizer. This stabilization irreversibly commits AECIIs to a mesenchymal phenotype, driving massive ECM accumulation. Using AECII‐specific *Virma* conditional knockout mice and pharmacological blockade with the p300 inhibitor C646, we confirmed the indispensable role of this axis in vivo. Collectively, our work delineates a novel p300/H3K18la/VIRMA/GATA3 signaling module, offering a comprehensive molecular framework for understanding how radiation‐induced metabolic reprogramming dictates long‐term fibrosis.

## 2 Results

### 2.1 m6A‐Mediated GATA3 Upregulation Drives RIPF by Promoting EMT

To investigate the mechanisms of radiation‐induced pulmonary fibrosis (RIPF), we established a murine model by delivering a single 20 Gy dose of thoracic x‐ray irradiation to C57BL/6J mice (Figure 1A). Gross morphological examination revealed that while wild‐type (WT) lungs remained smooth and healthy, RIPF lungs exhibited marked structural consolidation, characterized by a dark red hue and visible gray‐white fibrotic nodules. Consistently, longitudinal micro‐CT imaging demonstrated progressive high‐density opacification and linear fibrous bands in the irradiated group, evolving into extensive consolidation by 24 weeks (Figure 1B). Histopathological analysis via H&E staining at 4–8 weeks post‐irradiation revealed initial septal thickening and loss of alveolar integrity, indicative of acute radiation‐induced inflammation; by 24 weeks, profound interstitial hypercellularity and massive alveolar wall thickening were evident (Figure 1C). Masson's trichrome staining corroborated these findings, showing progressive blue collagen deposition peaking at 24 weeks (Figure 1D), which was further supported by significant elevation in hydroxyproline (HYP) content (Figure S1A). Molecular characterization via qPCR, western blotting (Figure 1E and Figure S1B), and in situ immunohistochemistry (Figure S1C,D) confirmed the upregulation of key extracellular matrix (ECM) markers, including Acta2 (α‐SMA) and Col1a1 (Collagen‐1), validating successful model establishment. Parallel in vitro irradiation of A549 and MLE‐12 cells similarly induced an epithelial‐mesenchymal transition (EMT) phenotype, evidenced by increased α‐SMA and Collagen‐1 expression (Figure 1F and Figure S1E). To elucidate the regulatory landscape, we performed epitranscriptomic and RNA sequencing. GO analysis of differentially expressed genes (DEGs) highlighted RNA processing and translation (Figure S1F), suggesting a pivotal role for RNA modifications. Global m6A levels were shown to rise in fibrotic lungs over time (Figure 1G,H). MeRIP‐seq identified the canonical “GGAC” motif and revealed m6A enrichment predominantly near stop codons and 3′ UTRs (Figure 1I and Figure S1G). Integrated analysis identified 740 hypermethylated m6A peaks, with 331 transcripts significantly upregulated (Figure 1J). Building on our previous work identifying GATA3 as a driver of RIPF, we hypothesized it is regulated by m6A. MeRIP‐seq confirmed significant m6A enrichment on Gata3 mRNA, correlating with its upregulation (Figure 1J–L and Figure S2A–C). Functionally, GATA3 suppression in A549 and MLE‐12 cells restored E‐cadherin expression and decreased radiation‐induced α‐SMA and Collagen‐1(Figure 1M and Figure S2D). Conversely, GATA3 overexpression increased EMT and ECM deposition (Figure S2E,F). Collectively, these results suggest that radiation‐induced m6A modification stabilizes or enhances GATA3 expression, thereby driving RIPF progression.

**FIGURE 1:**
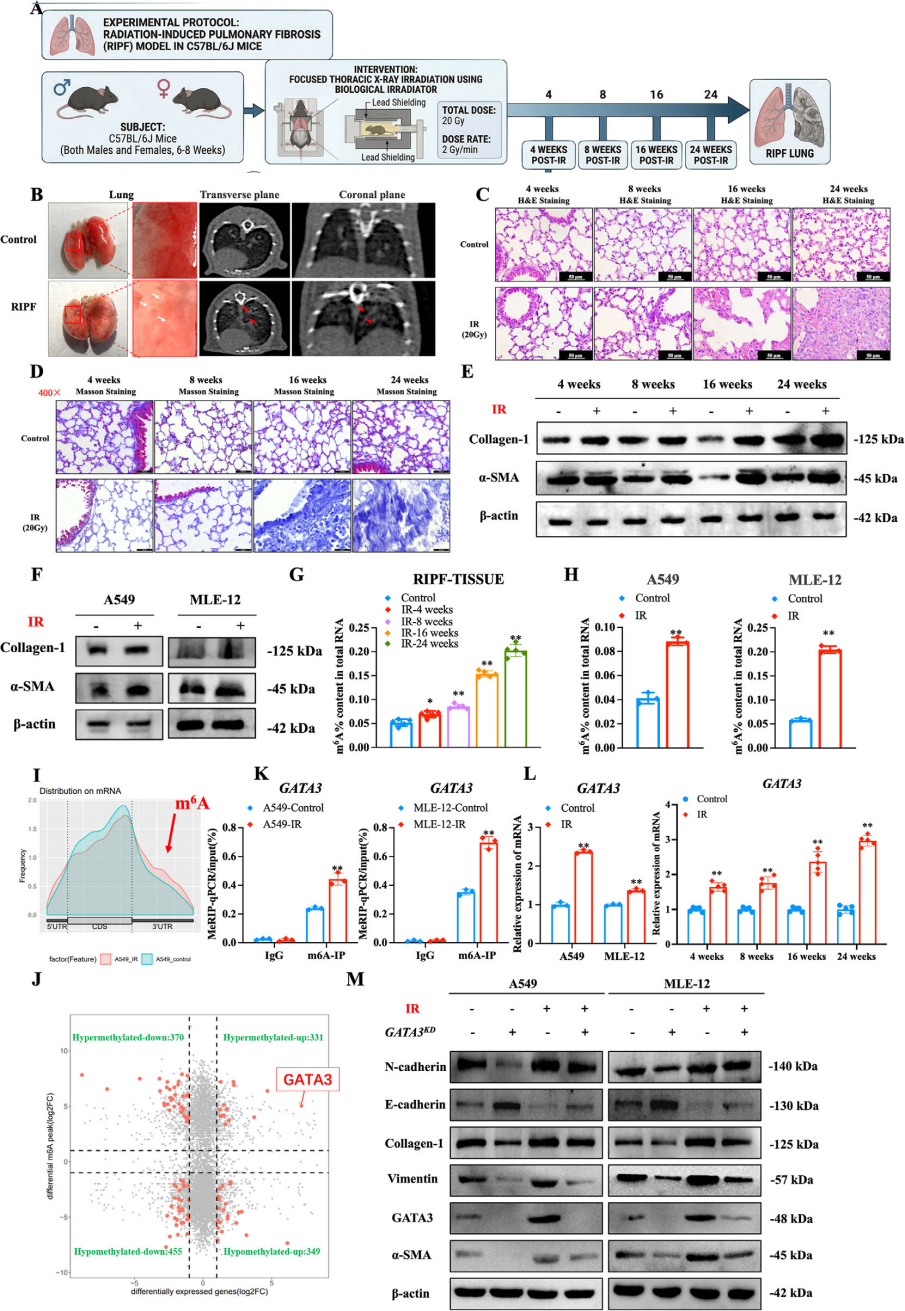
Increased m6A enrichment of *GATA3* in RIPF. (A) Flowchart of Radiation‐Induced Pulmonary Fibrosis. (B) Lung tissue and CT scans from the RIPF mice model. (C) H&E staining on paraffin section of RIPF mice model tissue. (D) Masson staining on paraffin section of RIPF mice model tissue. (E) The protein expression of α‐SMA and Collagen‐1 in RIPF mice model tissue by western blotting. (F) The protein expression of α‐SMA and Collagen‐1 in RIPF cell model by western blotting. (G) The levels of m6A in total RNA in RIPF mice model tissue. (H) The levels of m6A in total RNA in RIPF cell model. (I) Density distribution of m6A peaks across control and RIPF cell model. 5'UTR, 5' untranslated region; CDS, coding region; 3'UTR, 3' untranslated region. (J) Four quadrant graph of genes with differential m6A methylation and differentially expressed mRNA levels. (K) Perform immunoprecipitation of methylated RNA in cells using the m6A antibody, followed by qPCR analysis using primers targeting GATA3 mRNA. L: The mRNA level of GATA3 in the RIPF model. M: The protein expression of EMT and ECM marker by western blotting after GATA3 knockdown. The data are presented as the mean ± SD (*n* = 3 or 5), ^*^ *P* < 0.05 vs. control, ^**^ *P* < 0.01 vs. control.

### 2.2 VIRMA‐Dependent m6A Modification Mediates RIPF Progression

To identify the methyltransferase (“writer”) responsible for GATA3 m6A modification, we integrated differentially expressed genes (DEGs) from public datasets (GSE24206, GSE110147) with our RIPF RNA‐seq data. This analysis identified VIRMA as a top candidate gene, whose expression exhibited a strong positive correlation with GATA3 (Figure 2A,B). In our RIPF mouse and cellular models, VIRMA expression was significantly upregulated, whereas the elevation of METTL14 was negligible. These findings further validate VIRMA as a potential regulator of GATA3 (Figure 2C–F). Immunofluorescence (IF) analysis showed that radiation significantly increased the nuclear fluorescence intensity of VIRMA (Figure 2G). We further performed nuclear‐cytoplasmic fractionation assays, and the results indicated that nuclear VIRMA levels were markedly elevated compared with control cells, whereas cytoplasmic VIRMA abundance remained largely unchanged (Figure 2H). Mechanistically, radiation induced the assembly of F‐actin stress fibers, a vital cytoskeletal remodeling process that drives cell adhesion and epithelial‐mesenchymal transition (EMT). VIRMA knockdown reduced global m^6^A levels and dramatically diminished F‐actin stress fiber formation simultaneously (Figure 2I,J). Consequently, silencing *VIRMA* prevented the development of myofibroblast markers α‐SMA and Collagen‐1, limiting the EMT program and demonstrating its crucial involvement in RIPF progression (Figure 2K,L). We next assessed the organ‐ and cell‐type specificity of the VIRMA/GATA3 axis. Human Protein Atlas (HPA) analysis indicated that both proteins are expressed across diverse pulmonary cell populations (Figure S2A,B). Intriguingly, a cross‐organ meta‐analysis of fibrosis databases revealed that the synchronized upregulation and positive correlation of VIRMA and GATA3 were unique to pulmonary fibrosis (Figure S2C,D). Conversely, these markers exhibited a negative correlation in renal fibrosis (Figure S2E,F) and were absent or downregulated in hepatic and intestinal fibrosis (Figure S2G,H). These findings were validated in vitro using organ‐specific cells. After x‐ray irradiation of renal epithelial cells (HKC), hepatic cells (HSCT6, LX2), and intestinal epithelial cells (IEC‐6, HIEC‐6), the protein expression of α‐SMA and Collagen‐1 was markedly upregulated in these cells, indicating that radiation triggers fibrotic progression in the above cell types. Radiation failed to induce VIRMA in renal (HKC), hepatic (HSCT6, LX2), or intestinal (IEC‐6, HIEC‐6) epithelial cells, often resulting in GATA3 downregulation instead (Figure S2I,J). Similarly, in pulmonary endothelial cells (HUVEC) and fibroblasts (HFL‐1), radiation did not recapitulate the synchronized VIRMA/GATA3 elevation observed in lung epithelial cells (Figure S2K,M). Collectively, these data establish the VIRMA/GATA3 axis as a highly organotropic and cell‐specific regulatory module in radiation‐induced injury.

**FIGURE 2:**
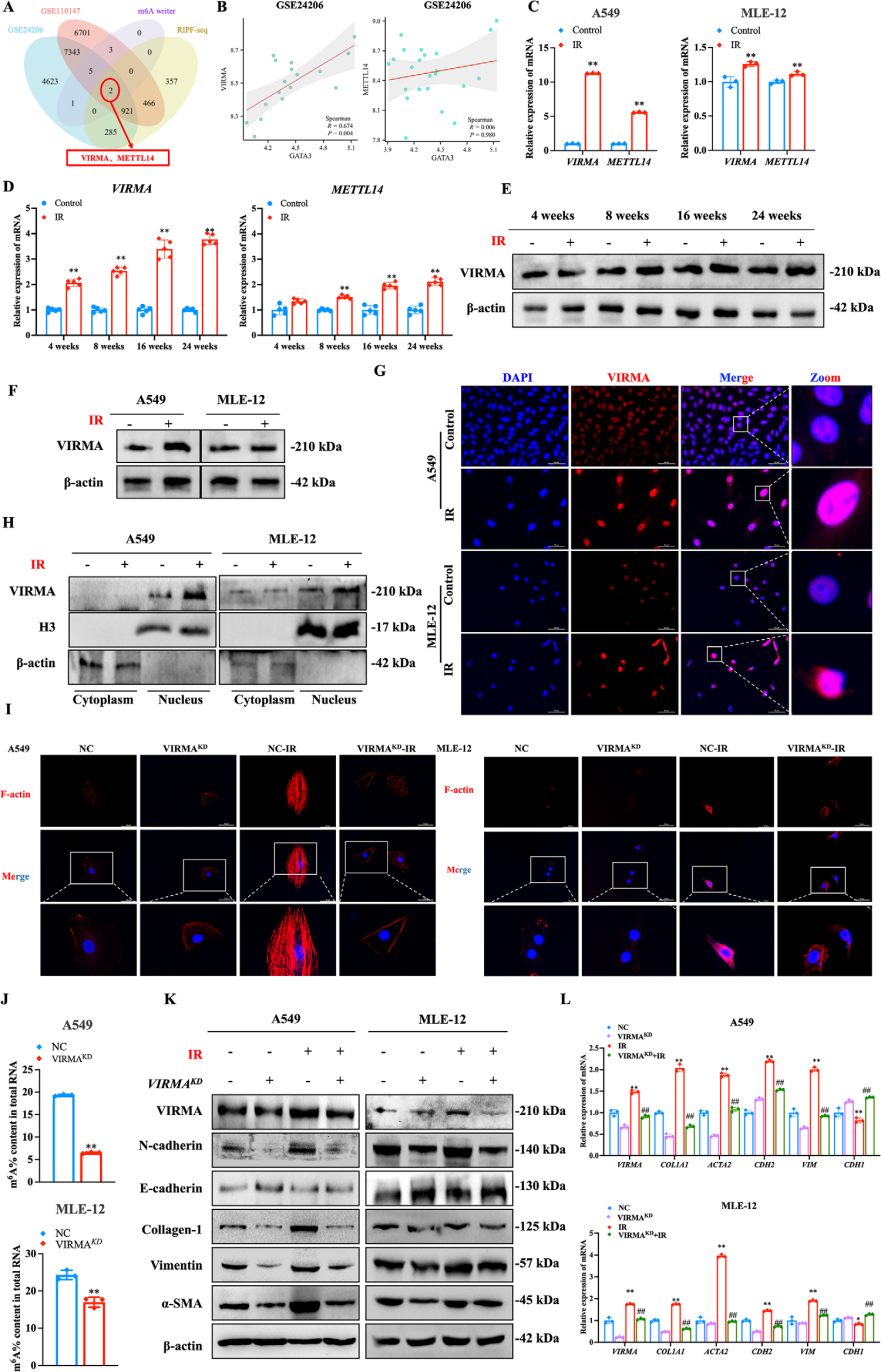
VIRMA regulates RIPF development through m6A modification. (A) Venn diagram showing the overlap of genes between the m6A dataset and differentially expressed genes in the cellular RIPF model RNA‐seq data and GEO database. (B) Correlation of GATA3 and VIRMA or METTL14 in the lung fibrosis database. (C) The mRNA level of VIRMA and METTL14 in the RIPF cell model. (D) The mRNA level of VIRMA and METTL14 in RIPF mice model tissue. (E) The protein expression of VIRMA in RIPF mice model. (F) The protein expression of VIRMA and METTL14 in the RIPF cell model. (G) The protein expression of VIRMA in RIPF cell model by Immunofluorescence. (H) Western blot analysis was used to examine the nuclear and cytoplasmic protein expression levels of GATA3 in a cellular model of RIPF. (I) The protein expression of F‐actin in RIPF cell model by Immunofluorescence. (J) The levels of m6A in total RNA after VIRMA knockdown. (K) The protein expression of EMT and ECM marker after VIRMA knockdown. L: The mRNA level of EMT and ECM marker after VIRMA knockdown. The data are presented as the mean ± SD (*n* = 3 or 5), ^*^ *P* < 0.05 vs. control, ^**^ *P* < 0.01 vs. control, ^#^ *P* < 0.05 vs. IR, ^##^ *P* < 0.01 vs. IR.

### 2.3 The VIRMA‐YTHDF1 Complex Synergistically Orchestrates m6A Modification of the *GATA3* 3′ UTR

To establish whether VIRMA directly targets *GATA3* mRNA, we performed RNA immunoprecipitation (RIP)‐qPCR, which demonstrated that VIRMA physically binds to GATA3 transcripts, an association significantly enhanced following radiation (Figure 3A). Visual inspection of MeRIP‐seq tracks confirmed a prominent m6A enrichment peak within the 3′ untranslated region (3′ UTR) of GATA3 (Figure 3B). To validate the functional impact of this modification, we constructed dual‐luciferase reporter plasmids harboring either the wild‐type (WT) or a mutated GATA3 3′ UTR, where the target adenosine residues within the identified m6A motifs were substituted (Figure 3C). Overexpression of *VIRMA* dramatically increased the WT reporter's luciferase activity but had no effect on the mutant construct, indicating that VIRMA‐mediated m6A alteration at the 3′ UTR is required for *GATA3* post‐transcriptional regulation (Figure 3D). Consistently, MeRIP‐qPCR confirmed that *VIRMA* knockdown markedly diminished m6A enrichment levels on the *GATA3* 3′ UTR (Figure 3E).

**FIGURE 3:**
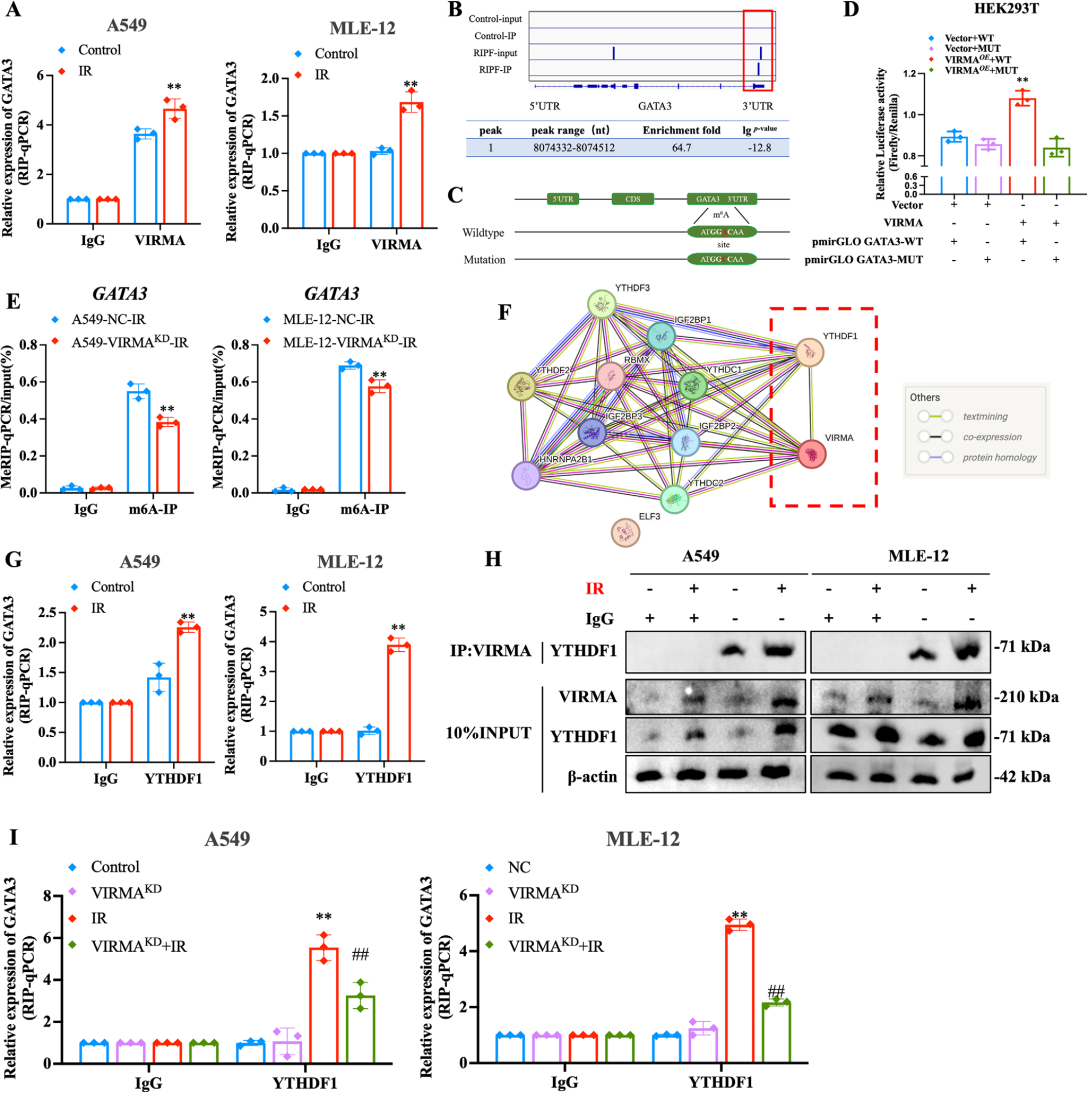
VIRMA/YTHDF1 regulates GATA3 expression through m6A modification. (A) RIP analysis of the interaction of VIRMA or m6A with GATA3 mRNA in RIPF cell model. The enrichment of GATA3 mRNA was measured by qRT‐PCR with antibodies targeted against VIRMA or m6A and normalized to the input. (B) The m6A peak visualization of m6A‐seq in GATA3 transcripts in A549 cells after radiation. The m6A peaks are in the 3’UTR of GATA3. (C) Two luciferase plasmids were constructed by inserting the corresponding 3′UTR into pmirGLO vectors. Wild‐type (WT) reporter constituted the full‐length 3′UTR of GATA3 with intact m6A sites, while the mutant one (MUT) carried one A‐C mutation on the first predicted m6A site. (D) Luciferase vectors with the wild‐type (WT) or mutated m6A nucleotides (MT) in the GATA3 gene were transfected into 293 T cells after VIRMA overexpression. Relative luciferase activity was measured. (E) Methylated RNA in cells VIRMA knockdown depletion was immunoprecipitated with an m6A antibody, followed by qPCR analyses with primers against GATA3 mRNA. (F) The PPI website predicts the binding between the VIRMA protein and the m6A‐binding reader. (G) RNA‐Binding Protein Immunoprecipitation (RIP) followed by RT‐qPCR was conducted to determine whether YTHDF1 binds to GATA3 mRNA. (H) Immunoprecipitation assay examining the binding of VIRMA to YTHDF1 in irradiated cells. I: Binding of YTHDF1 protein to GATA3 mRNA in cells subjected to combined VIRMA knockdown and irradiation. The data are presented as the mean ± SD (*n* = 3), ^*^ *P* < 0.05 vs. control, ^**^ *P* < 0.01 vs. control, ^#^ *P* < 0.05 vs. IR, ^##^ *P* < 0.01 vs. IR.

Since m6A “writers” typically function in concert with specific “readers” to dictate mRNA fate, we used protein‐protein interaction (PPI) databases to identify potential co‐factors, which predicted an interaction between VIRMA and the m6A reader YTHDF1 (Figure 3F). Subsequent RIP‐qPCR assays confirmed that YTHDF1 directly binds to *GATA3* mRNA, an interaction further intensified by radiation (Figure 3G). Given these findings, we hypothesized that VIRMA and YTHDF1 form a functional complex to coordinately regulate GATA3. Co‐immunoprecipitation (Co‐IP) assays in A549 and MLE‐12 cells validated this model, showing that radiation exposure significantly promotes the assembly of the VIRMA‐YTHDF1 complex (Figure 3H). Crucially, silencing VIRMA not only disrupted the VIRMA‐YTHDF1 protein interaction but also reduced the recruitment of the complex to *GATA3* mRNA (Figure 3I). Together, these results establish that radiation triggers the formation of a VIRMA‐YTHDF1 complex to drive the m6A‐dependent upregulation of GATA3 during pulmonary fibrosis.

### 2.4 VIRMA‐Mediated m6A Stabilization of GATA3 mRNA Drives RIPF

To elucidate the regulatory mechanism by which VIRMA controls GATA3, we employed VIRMA‐silenced A549 and MLE‐12 cell lines. The genetic knockdown of VIRMA significantly reduced GATA3 expression at both the transcript and protein levels (Figure 4A,B). Since m6A modifications predominantly dictate mRNA fate by modulating stability, we performed RNA decay assays using actinomycin D (Act D). VIRMA silencing significantly accelerated the degradation of GATA3 mRNA, shortening its half‐life compared to control groups (Figure 4C). These data demonstrate that VIRMA‐mediated m6A modification enhances GATA3 expression by increasing its post‐transcriptional stability, thereby preventing the rapid turnover of GATA3 transcripts in a methyltransferase‐dependent manner.

**FIGURE 4:**
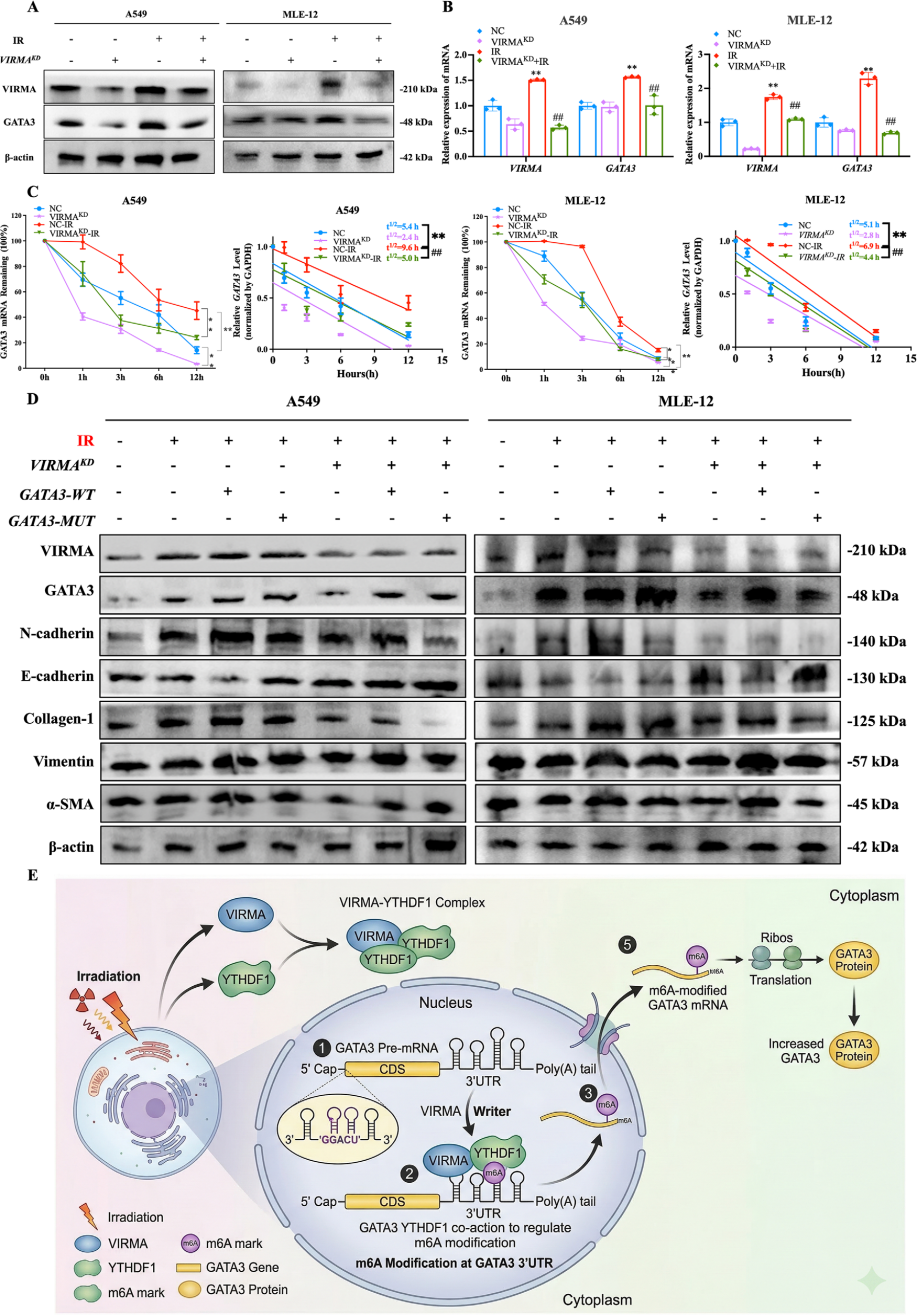
VIRMA promotes GATA3 mRNA stability during the RIPF process. (A) The protein expression of GATA3 after VIRMA knockdown. (B) The mRNA level of GATA3 after VIRMA knockdown. (C) GATA3 mRNA expression was detected after incubation with 5 µg/mL actinomycin D for the indicated times and normalized to β‐actin. The mRNA half‐lives were estimated according to linear regression analysis after the indicated actinomycin D treatment. (D) The protein expression of EMT and ECM markers after VIRMA knockdown and GATA3 (WT or MUT) overexpression. (E) Schematic diagram illustrating the mechanism by which VIRMA/YTHDF1 enhances GATA3 stability through m6A modification. The data are presented as the mean ± SD (*n* = 3), ^*^ *P* < 0.05 vs. NC, ^**^ *P* < 0.01 vs. NC, ^#^ *P* < 0.05 vs. IR or VIRMA^KD^+IR, ^##^ *P* <0.01 vs. IR or VIRMA^KD^+IR.

To further investigate how the VIRMA/GATA3 axis drives radiation‐induced epithelial‐mesenchymal transition (EMT) and extracellular matrix (ECM) deposition, we performed rescue experiments by constructing two overexpression plasmids: GATA3‐WT (containing the CDS and 3'UTR regions of GATA3) and GATA3‐MUT (carrying point mutations at the m^6^A modification sites within the GATA3 3'UTR). Following VIRMA knockdown, cells were transfected with GATA3 3'UTR‐WT or GATA3 3'UTR‐Mut constructs and subsequently exposed to x‐ray irradiation.

Western blot results revealed that relative to the IR group, the protein levels of α‐SMA, Collagen I, N‐cadherin, and Vimentin were elevated, while E‐cadherin expression was reduced in the GATA3‐WT+IR group. In contrast, no significant alterations in EMT and ECM‐related markers were observed in the GATA3‐MUT+IR group. Compared with the IR group, the VIRMA KD+IR group exhibited markedly decreased expression of α‐SMA, Collagen I, N‐cadherin and Vimentin, accompanied by increased E‐cadherin levels. Notably, concurrent VIRMA knockdown and GATA3‐WT overexpression restored the upregulation of α‐SMA, Collagen I, N‐cadherin and Vimentin, whereas overexpression of GATA3‐Mut failed to exert this rescue effect (Figure 4D). In irradiation A549 and MLE‐12 cells, VIRMA knockdown restored epithelial indicators E‐cadherin while lowering mesenchymal markers (N‐cadherin, Vimentin) and ECM markers (α‐SMA, Collagen‐1), thereby abrogating the fibrotic phenotype. Crucially, exogenous overexpression of GATA3 partially rescued these effects, re‐initiating the EMT program and reversing the anti‐fibrotic protection conferred by VIRMA deficiency (Figure S4A,B). Collectively, our findings establish a novel mechanistically linked cascade: radiation‐induced VIRMA coordinates with YTHDF1 to target the GATA3 3′ UTR for m6A modification, which stabilizes the transcript and upregulates GATA3 expression. This axis subsequently triggers the EMT program and massive ECM accumulation, identifying the VIRMA/YTHDF1/GATA3 signaling module as a pivotal driver and therapeutic vulnerability in RIPF (Figure 4E).

### 2.5 AECII‐Specific VIRMA Ablation Attenuates RIPF by Disrupting the m6A‐GATA3 Axis

To rigorously evaluate the in vivo requirement of VIRMA in RIPF, we generated an alveolar type II epithelial cell (AECII)‐specific conditional knockout (cKO) mouse model by crossing *Virma* ^fl/fl^ mice with Sftpc‐CreERT2 transgenic mice. Following tamoxifen induction and subsequent thoracic irradiation (Figure 5A), successful AECII‐specific ablation of VIRMA was validated via immunohistochemistry (IHC), which confirmed a profound reduction of VIRMA protein and mRNA levels in the KO lung parenchyma (Figure 5B,C). Mechanistically, VIRMA depletion markedly attenuated radiation‐triggered global m^6^A modification and repressed m^6^A enrichment on GATA3 mRNA, which consequently led to reduced GATA3 expression (Figure 5D–F). Histopathological analysis via H&E staining revealed that, compared to the IR‐only group, *Virma* cKO mice exhibited better preserved alveolar architecture, reduced septal thickening, and diminished inflammatory infiltration (Figure 5G). Masson's trichrome staining further demonstrated that *Virma* loss abrogated interstitial collagen deposition and structural remodeling (Figure 5H). VIRMA depletion significantly reduced hydroxyproline content (Figure 5I). At the molecular level, IHC and qPCR analyses confirmed that AECII‐specific *Virma* deletion significantly suppressed the induction of extracellular matrix (ECM) markers (*Acta2, Col1a1*) and mesenchymal signatures (*Cdh2, Vim*) (Figure 5J,K). Collectively, these genetic loss‐of‐function data demonstrate that VIRMA orchestrates the m6A/GATA3 axis within the AECII compartment to drive epithelial‐to‐mesenchymal transition (EMT) and fibrotic progression, highlighting VIRMA as a pivotal therapeutic target for alleviating the pathological features of RIPF.

**FIGURE 5:**
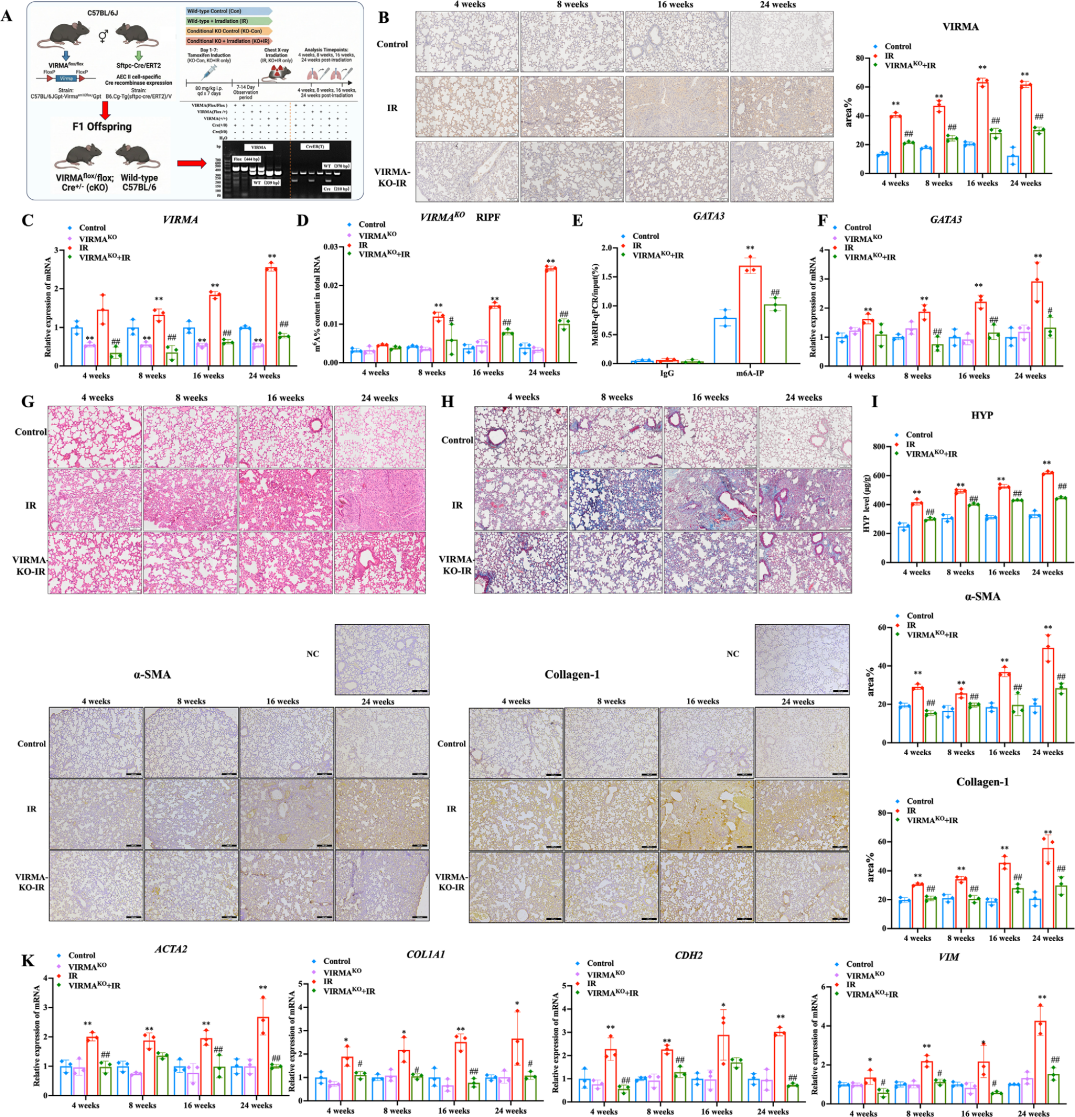
Knockout of VIRMA inhibits RIPF processes. (A) Schematic diagram of the construction of a mouse model with a *Virma* gene knockout in type II alveolar epithelial cells. (B) Immunohistochemistry was used to detect the expression of VIRMA protein in *Virma* cKO RIPF mice model tissue. (C) The mRNA level of VIRMA in *Virma* cKO RIPF mice model tissue. (D) The levels of m6A in total RNA in *Virma* cKO RIPF mice model tissue. (E) Methylated RNA in VIRMA knockout mice depletion was immunoprecipitated with an m6A antibody, followed by qPCR analyses with primers against GATA3 mRNA. (F) The mRNA level of GATA3 in *Virma* cKO RIPF mice model tissue. (G) H&E staining on paraffin section of *Virma* cKO RIPF mice model tissue. (H) Masson staining on paraffin section of *Virma* cKO RIPF mice model tissue. I: The expression of HYP in *Virma* cKO RIPF mice model tissue. (J) Immunohistochemistry was used to detect the expression of α‐SMA and Collagen‐1 protein in *Virma* cKO RIPF mice model tissue. (K) The mRNA level of EMT and ECM in *Virma* cKO RIPF mice model tissue. The data are presented as the mean ± SD (*n* = 3), ^*^ *P* < 0.05 vs. control, ^**^ *P* < 0.01 vs. control, ^#^ *P* < 0.05 vs. VIRMA^KO^+IR, ^##^ *P* < 0.01 vs. VIRMA^KO^+IR.

### 2.6 Metabolic‐Epigenetic Crosstalk via H3K18la Mediates Radiation‐Induced VIRMA Activation

To elucidate the precise mechanisms underlying VIRMA upregulation, we conducted further investigations. Given that histones are integral components of chromatin, their lactylation dynamically regulates chromatin accessibility, altering histone structure, function, and interactomes to modulate downstream target gene expression. Accordingly, our RNA‐Seq data's KEGG pathway analysis showed a high enrichment in glycolysis‐related pathways (Figure 6A). Interrogation of publicly available transcriptomic datasets (GSE110147 and GSE24206) further demonstrated an upregulation of LDHA and a concomitant downregulation of MCT4 in fibrotic tissues. This suggests a shift toward aerobic glycolysis and a pathological intracellular accumulation of lactate in profibrotic cells (Figure S5A). Consistent with this, in our in vitro RIPF cellular model, radiation exposure significantly elevated the extracellular acidification rate (ECAR) and lactate production while depleting intracellular ATP levels. Concurrently, radiation induced the upregulation of key glycolytic enzymes and transporters (LDHA, GLUT1, HK1, and MCT1) alongside the downregulation of MCT4 (Figure 6B and Figure S5B–D), collectively underscoring a radiation‐induced glycolytic switch. Histone lactylation (Kla) is a new epigenetic post‐translational modification that uses intracellular lactate as its principal substrate, we assessed global Kla levels, which were elevated in RIPF cells (Figure 6C). To establish causality, we treated lung epithelial cells with exogenous sodium lactate (Lactate) to mimic a lactate‐rich environment, or co‐treated irradiated cells with dichloroacetate (DCA), an inhibitor of lactate production. As expected, irradiated (IR) cells exhibited significantly enhanced ECAR and Kla levels compared to the control group, phenocopying the lactate‐treated group. Strikingly, treatment with DCA (IR+DCA) completely abrogated these radiation‐induced metabolic and epigenetic alterations (Figure 6D,E and Figure S5E). Furthermore, lactate restriction via DCA significantly reduced radiation‐induced induction of VIRMA and GATA3, as well as reversed the expression of EMT and ECM markers (Figure 6F and Figure S5F). These findings indicate that VIRMA transcription is tightly coupled to histone lactylation. To identify the specific histone targets mediating VIRMA transcription, we performed immunoprecipitation assays. Quantitative analysis demonstrated that radiation specifically increased the levels of lactylation on histone H3 (Figure 6G). By interrogating the CPLM and UniProt databases, we identified highly conserved and high‐confidence lactylation sites across human and mouse species (Figure 6H). Subsequent biochemical validation confirmed that H3K18la levels were elevated in both in vitro and in vivo RIPF models (Figure 6I). However, in A549 cells, H3K9la levels were slightly decreased while H3K14la levels were increased in the IR group compared with the control group. No obvious changes in these two histone modifications were observed in MLE‐12 cells (Figure S5G). Taken together, lysine 18 (K18) was identified as the candidate modification site. Importantly, DCA treatment markedly abrogated the radiation‐induced accumulation of H3K18la (Figure 6J), leading us to hypothesize that H3K18la directly drives VIRMA transcription. To delineate the specific transcriptional regulation of VIRMA by H3K18la, we integrated publicly available H3K18la ChIP‐seq datasets (GSE171832 and GSE156675). This analysis revealed an enrichment of H3K18la at the VIRMA promoter region. Subsequent ChIP‐qPCR assays in A549 and MLE‐12 cells corroborated that radiation exposure significantly augmented H3K18la occupancy at the VIRMA promoter, thereby driving its transcriptional upregulation (Figure 6K and Figure S5H,I). Taken together, these findings illuminate a novel epigenetic‐metabolic axis wherein radiation‐induced metabolic reprogramming leads to H3K18la modification, which directly transactivates VIRMA expression to sustain the profibrotic cascade in RIPF.

**FIGURE 6:**
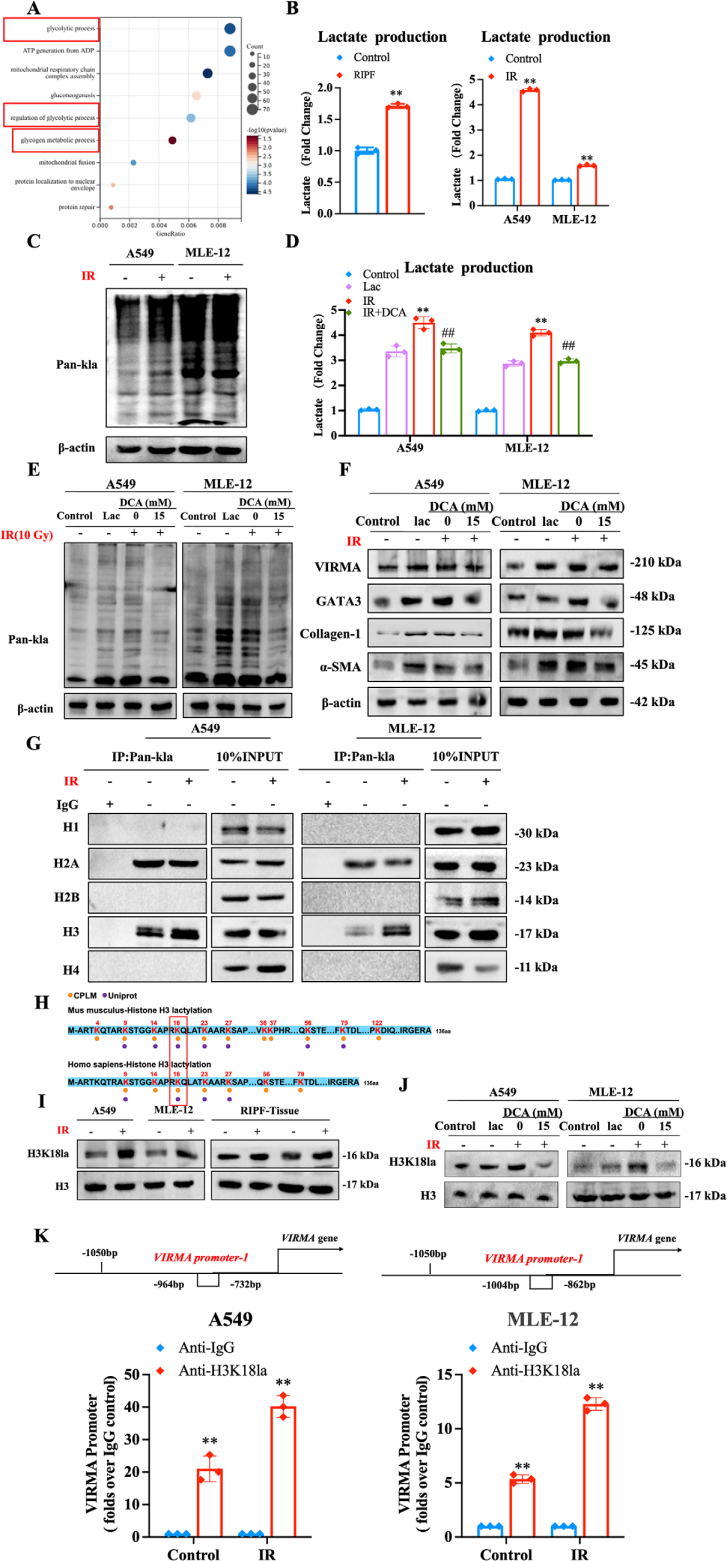
Radiation‐induced metabolic reprogramming transactivates VIRMA via H3K18la‐mediated epigenetic modification. (A) KEGG pathway enrichment analysis of RNA‐seq data derived from the RIPF cellular model. (B) Quantification of intracellular lactate levels in control and irradiated RIPF cells. (C) Immunoblotting analysis of global lactylation (Pan‐Kla) levels in the in vitro RIPF model. (D) Assessment of intracellular lactate production in irradiated cells following treatment with exogenous sodium lactate (lac) or dichloroacetate (DCA). (E) Western blotting analysis of Pan‐Kla expression in irradiated cells following treatment with exogenous sodium lactate (lac) or dichloroacetate (DCA). (F) Protein expression of VIRMA, GATA3, and classical extracellular matrix (ECM) markers assessed by immunoblotting in cells treated with radiation, Lac, or DCA. (G) Co‐immunoprecipitation (Co‐IP) assay demonstrating the specific enrichment of lactylation on histone H3 following radiation exposure. (H) Bioinformatics identification of highly conserved lactylation modification sites on histone H3 using the CPLM and UniProt databases, pinpointing the K18 residue. (I) Representative immunoblot showing H3K18la protein levels in the RIPF model. (J) Western blotting analysis illustrating the dynamic changes in H3K18la expression in irradiated cells co‐treated with lac or DCA. (K) ChIP‐qPCR was performed in A549 and MLE‐12 using IgG and H3K18la antibodies, followed by qRT‐PCR with primers of the VIRMA promoter region specific to H3K18la. The data are presented as the mean ± SD (*n* = 3), ^*^ *P* < 0.05 vs. control, ^**^ *P* < 0.01 vs. control, ^#^ *P* < 0.05 vs. IR, ^##^ *P* < 0.01 vs. IR.

### 2.7 Targeting the p300/H3K18la/VIRMA Axis Alleviates RIPF

Lactyl groups are transferred from lactyl‐CoA to histones by the lysine lactyltransferase p300. We observed that p300 expression is significantly upregulated at both the mRNA and protein levels in RIPF cell models (Figure 7A). ChIP‐qPCR analysis revealed that *p300* knockdown (KD) markedly reduced H3K18la occupancy at the *VIRMA* promoter compared to irradiated controls (Figure 7B), suggesting that p300‐mediated lactylation directly drives VIRMA transcription. Functional rescue experiments in A549 and MLE‐12 cells confirmed this axis: knocking down *p300* restores E‐cadherin while decreasing mesenchymal markers (N‐cadherin, Vimentin) and myofibroblast signatures (α‐SMA, Collagen‐1), thereby preventing radiation‐induced EMT. Critically, exogenous *VIRMA* overexpression partially rescued the fibrotic phenotype (Figure 7C), whereas *VIRMA* KD impeded the EMT program initiated by p300 overexpression (Figure S6A). These findings establish the p300/H3K18la/VIRMA axis as a key driver of fibrotic remodeling. To validate the in vivo therapeutic potential of targeting this axis, we used the p300 inhibitor C646 in a murine RIPF model. IHC confirmed successful p300 inhibition in lung tissues (Figure 7D). Longitudinal CT imaging demonstrated that C646 treatment significantly attenuated radiation‐induced ground‐glass opacities (Figure 7E and Figure S7C). In p300‐inhibited mice, histopathological examination with H&E and Masson's trichrome staining indicated maintained alveolar architecture, reduced septal thickness, and a decrease in interstitial collagen deposition compared to the IR‐only group (Figure 7F,G and Figure S7D,E). Furthermore, p300 inhibition significantly suppressed the expression of key ECM markers, including Collagen‐1 and α‐SMA (Figure 7H and Figure S7F,G). In summary, radiation‐induced p300 upregulation catalyzes H3K18 lactylation at the VIRMA promoter, thereby activating the VIRMA/m6A program to drive pulmonary fibrogenesis (Figure 7I).

**FIGURE 7:**
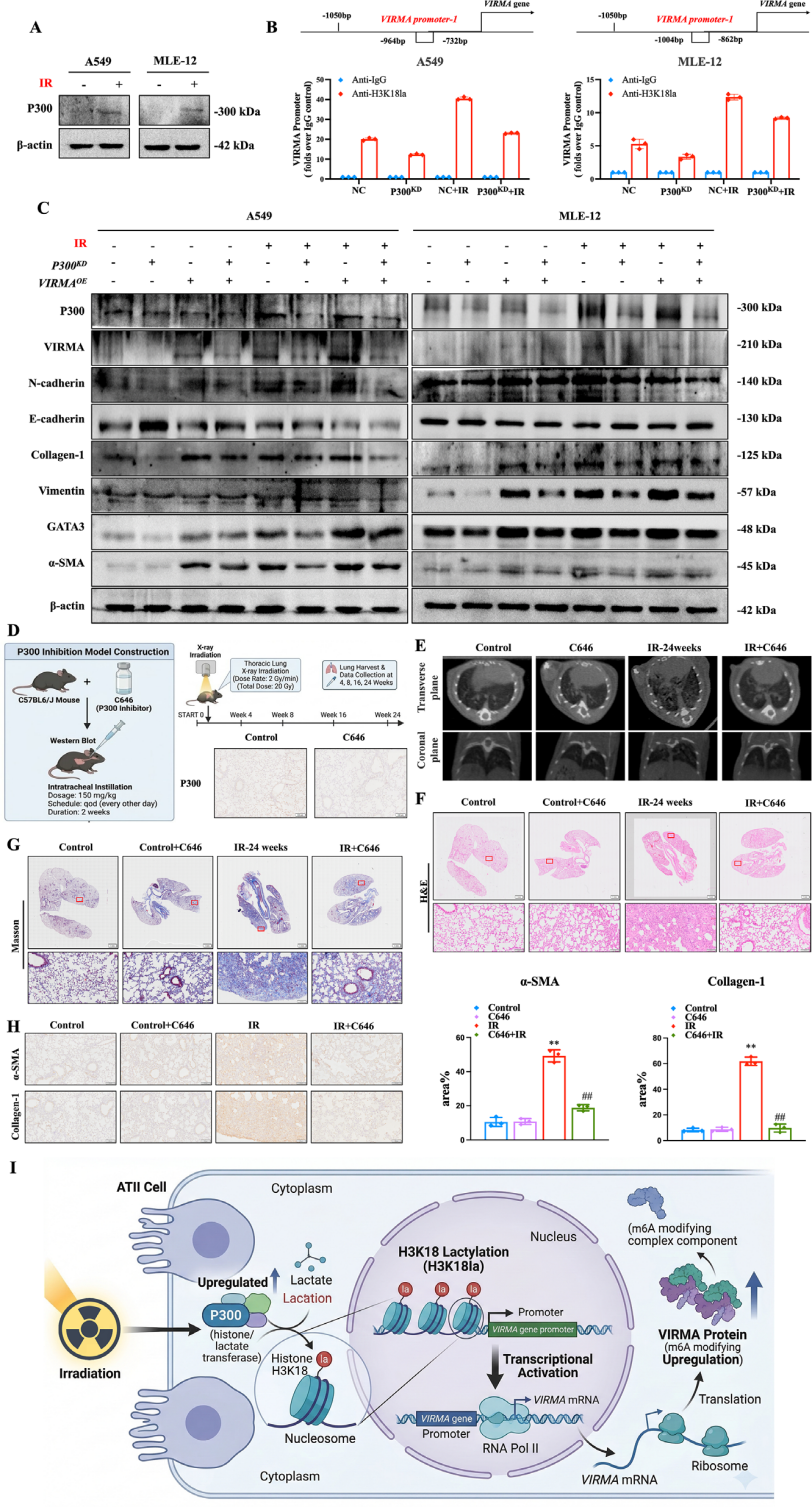
P300 promotes the H3K18la‐mediated regulation of the RIPF process. (A) The protein expression of P300 in RIPF cell model by western blotting. (B) ChIP‐qPCR was performed in *P300* knockdown cell using IgG and H3K18la antibodies, followed by qRT‐PCR with primers of the *VIRMA* promoter region specific to H3K18la. (C) The protein expression EMT and ECM of after *P300* knockdown or *VIRMA* overexpression. (D) Flowchart of the process for generating a P300‐inhibited RIPF mice lung tissue model using the P300 inhibitor C646. (E) CT scan of lung tissue from a P300‐inhibited RIPF mice model. (F) H&E staining of lung tissue from a P300‐inhibited RIPF mice model. (G) Masson staining of lung tissue in a P300‐inhibited RIPF mice model. (H) P300 inhibits the in situ expression of α‐SMA and collagen‐1 in lung tissue from RIPF mice. (I) Schematic diagram illustrating the mechanism by which P300 regulates VIRMA expression through the promotion of H3K18a during the RIPF process. The data are presented as the mean ± SD (*n* = 3), ^*^ *P* < 0.05 vs. IgG, ^**^ *P* < 0.01 vs. IgG.

## 3 Discussion

The pathogenesis of radiation‐induced pulmonary fibrosis (RIPF) has long been framed through the lens of acute DNA damage and subsequent cytokine cascades [25, 26]. However, this classical paradigm fails to explain the chronic, self‐perpetuating nature of fibrogenesis that persists long after radiation exposure. Here, we delineate a novel “metabolic‐epigenetic‐epitranscriptomic” axis that functions as a molecular “memory” switch, locking alveolar type II epithelial cells (AECIIs) into a persistent pro‐fibrotic state. By integrating glycolytic reprogramming, histone lactylation, and m6A‐mediated mRNA stabilization, our findings offer a multi‐layered regulatory framework that redefines the molecular landscape of RIPF.

Our investigation into this epitranscriptomic layer was rooted in our previous findings. We previously identified the transcription factor GATA3 as a critical driver of RIPF via the GATA3/NRP1 pathway; however, the precise mechanism sustaining its aberrant expression following radiation remained elusive. To address this, we first investigated global epitranscriptomic shifts and observed significantly elevated m6A levels in total RNA within our RIPF models. Integrating high‐throughput MeRIP‐seq with RNA‐seq pinpointed significant m6A enrichment specifically within the 3′ untranslated region (3′UTR) of *GATA3*. To identify the catalytic driver of this modification, we employed a robust bioinformatic screening strategy: intersecting differentially expressed genes from two independent GEO fibrosis datasets with known m6A “writers.” This, coupled with a targeted correlation analysis, identified VIRMA (KIAA1429) as the key enzyme responsible for the radiation‐induced m6A landscape.

Subsequent mechanistic dissection revealed that VIRMA recruits the “reader” YTHDF1 to form a functional complex that co‐localizes at a conserved m6A motif within the GATA3 3′UTR. Unlike traditional m6A‐mediated decay [27, 28], this modification functions as a post‐transcriptional stabilizer, significantly prolonging GATA3 mRNA half‐life. This finding provides the missing link in our understanding of RIPF, demonstrating that while radiation initiates GATA3 transcription, the VIRMA/YTHDF1‐mediated epitranscriptomic stabilization is what “locks” its expression, thereby driving the persistent EMT program in AECIIs.

Notably, the VIRMA/GATA3 axis exhibits a degree of organ‐ and cell‐type specificity. Although the components of the m6A machinery are ubiquitously expressed, the synchronized activation of this specific cascade appears unique to the post‐radiation pulmonary microenvironment, a phenomenon notably absent in liver or intestinal tissues. This suggests that AECIIs possess a unique “epigenetic plasticity”.

A pivotal question arising from these observations was the upstream mechanism driving VIRMA upregulation. This led us to identify p300‐mediated histone lactylation (H3K18la) as the epigenetic bridge connecting radiation‐induced metabolic shifts to epitranscriptomic activation. While the “Warburg‐like” glycolytic shift is a known feature of various fibrotic diseases [29, 30], our findings provide the first indication that radiation‐induced lactate buildup is not a metabolic waste, but rather a strong signaling substrate. Specifically, we demonstrate that p300 exhibits lactyltransferase activity under radiation stress, catalyzing H3K18la at the VIRMA promoter. This site‐specific modification functions as a “metabolic‐epigenetic sensor,” directly coupling the cell's bioenergetic state to the activation of the m6A machinery. The efficacy of the p300 inhibitor C646 in attenuating fibrotic progression in vivo further underscores the therapeutic potential of targeting this metabolic‐to‐epitranscriptomic relay.

Despite these insights, several questions warrant further investigation. While we focused on H3K18la, the potential contribution of other lactylation sites (e.g., H3K9la or H3K14la) to the broader fibrotic landscape remains plausible. Distinct organs display differential radiosensitivity, and equivalent radiation doses may induce fibrotic lesions of varying severity in different tissues, which constitutes an inherent limitation of the present work. In subsequent studies, combined TGF‐β treatment and irradiation will be applied to multiple organ tissues to further clarify the regulatory effects of the P300/H3K18la/VIRMA/GATA3 axis on fibrotic progression across diverse organs.

In summary, our study delineates a multi‐layered regulatory cascade that drives the irreversible progression of RIPF. We demonstrate that ionizing radiation triggers a glycolytic shift in pulmonary epithelial cells, leading to pathological lactate accumulation. This metabolic surge is durably encoded into the epigenome via p300‐mediated H3K18la, which modulates chromatin accessibility to facilitate VIRMA transcription. Once upregulated, VIRMA recruits the reader YTHDF1 to form an epitranscriptomic complex that targets the *GATA3* 3′UTR. By orchestrating site‐specific m6A modifications, this complex functions as a post‐transcriptional stabilizer, sustaining *GATA3* overexpression and the subsequent EMT program. Collectively, our findings define this p300/H3K18la/VIRMA/GATA3 relay as a key regulator in fibrogenesis, offering a robust molecular framework and a promising therapeutic rationale for the treatment of RIPF.

## 4 Materials and Methods

### 4.1 Animals and Treatment

The Jilin University Experimental Animal Center provided male C57BL/6 mice that were specific pathogen‐free (SPF) and six weeks old. Mice were kept under controlled circumstances (22°C–25°C, 50% humidity), with a 12‐h light/dark cycle and free access to food and drink. All animal procedures were approved by the Ethics Committee of Jilin University and conducted in accordance with the *Rules for the Management of Laboratory Animals*.

To establish the radiation‐induced pulmonary fibrosis (RIPF) model, mice were anesthetized and subjected to a single dose of 20 Gy thoracic x‐ray irradiation (2.0 Gy/min) using an X‐RAD320 irradiator. Extrathoracic regions were shielded with lead plates to minimize collateral damage. For pharmacological studies, mice were treated with the p300 inhibitor C646 (dosage:150 mg/kg, route: intratracheal instillation).

To create AECII‐specific Virma knockout mice, *Virma^fl/fl^* mice were crossed with Sftpc‐CreERT2 transgenic mice. Gene deletion was induced by intraperitoneal injection of tamoxifen (dose) for five consecutive days prior to irradiation. Body weight and health status were monitored regularly. Mice were euthanized at indicated time points (up to 24 weeks) for lung tissue collection.

### 4.2 Cell Culture and Irradiation

The mouse lung epithelial cell line MLE‐12 and human alveolar epithelial cell line A549 were purchased from Shanghai FH Biotechnology Co., Ltd. Cells were screened to ensure they were mycoplasma‐free. MLE‐12 cells were cultivated in DMEM, whereas A549 cells were cultured in RPMI‐1640 media. Both were supplemented with 10% FBS and incubated at 37°C with 5% CO2. For in vitro irradiation, A549 cells received 10 Gy (1.0 Gy/min) and MLE‐12 cells received 6 Gy (0.75 Gy/min) using the X‐RAD320 irradiator at room temperature.

### 4.3 Nuclear and Cytoplasmic Protein Extraction

Cytoplasmic and nuclear proteins were extracted from cultured cells using Beyotime Nuclear‐Cytoplasmic Separation Kit (Cat. No. P0027, Beyotime Biotechnology, Shanghai, China) in strict accordance with the manufacturer's instructions. Briefly, cells were washed twice with pre‐cooled PBS, scraped and pelleted by centrifugation at 500 × g for 5 min at 4°C. Cell pellets were resuspended in ice‐cold cytoplasmic extraction reagent supplemented with 1 mm PMSF and protease inhibitor cocktail, followed by 10–15 min incubation on ice with intermittent gentle vortexing. After centrifugation at 12 000 × g for 5 min at 4°C, the supernatant was collected as cytoplasmic protein fraction. The residual nuclear pellet was washed once with cold cytoplasmic extraction buffer to remove cytoplasmic impurities, resuspended in ice‐cold nuclear extraction reagent containing fresh PMSF and protease inhibitors, and incubated on ice for 30 min with vigorous vortexing every 5 min. The mixture was then centrifuged at 12 000 × g for 10 min at 4°C, and the supernatant was harvested as purified nuclear proteins. Protein concentrations of both fractions were quantified via BCA assay, and all protein samples were denatured with SDS loading buffer at 100°C for 10 min before western blot analysis.

### 4.4 Histology and Immunohistochemistry (IHC)

Hematoxylin and Eosin (H&E) staining was used to evaluate general morphology after lung tissues were fixed in 4% paraformaldehyde, embedded in paraffin, and sectioned at 5 µm (Solarbio, Beijing, China). Collagen deposition was evaluated using a Masson's Trichrome Stain Kit (Jiancheng, Nanjing, China). For IHC, sections were incubated with primary antibodies against Collagen‐I (Bioworld Tech, BS1530) and α‐SMA (Bioworld Tech, BS70000) using a streptavidin‐peroxidase kit (MXB, Fuzhou, China) according to the manufacturer's instructions.

### 4.5 Hydroxyproline (HYP) Content Assay

To determine total collagen content, the moist weight of lung tissues was measured, followed by alkaline hydrolysis with a Hydroxyproline Assay Kit (Jiancheng, Nanjing, China) according to the manufacturer's instructions. Absorbance was measured at 550 nm.

### 4.6 RNA Extraction and RT‐qPCR

Total RNA was extracted from tissues and cells using TRIzol reagent (Invitrogen). cDNA was synthesized using a reverse transcription kit, and quantitative real‐time PCR (qRT‐PCR) was performed to analyze gene expression levels.

### 4.7 Global m6A Quantification

Total m6A levels in mRNA were quantified using the EpiQuik m6A RNA Methylation Quantification Kit (Epigentek, NY, USA). Briefly, 200 ng of isolated RNA per sample was bound to assay wells. The m6A content was detected colorimetrically by measuring the absorbance at 450 nm, following the manufacturer's instructions.

### 4.8 m6A‐seq (MeRIP‐seq)

Total RNA was fragmented into approximately 100‐nt pieces. A subset of the fragmented RNA was stored as “input.” The remaining RNA was immunoprecipitated using an anti‐m6A antibody. The enriched RNA fragments were recovered via phenol‐chloroform extraction and used to construct libraries for ultralow input RNA methylation sequencing. Library quality and size distribution were assessed using a Bioptic Qsep100 Analyzer. High‐throughput sequencing was performed on the Illumina NovaSeq PE150 platform.

### 4.9 Immunofluorescence (IF)

Cells or slices were fixed in 4% paraformaldehyde, then permeabilized in 0.5% Triton X‐100 in PBS and blocked with 5% goat serum. Samples were incubated with an anti‐VIRMA antibody (Novus Biologicals) overnight at 4°C, followed by incubation with fluorophore‐conjugated secondary antibodies. Nuclei were counterstained with DAPI.

### 4.10 Luciferase Reporter Assay

The 3' UTR of the *GATA3* transcript containing the wild‐type (WT) m6A consensus sequence was cloned into the pmirGLO vector (pmirGLO‐GATA3‐WT). A mutant version (pmirGLO‐GATA3‐MUT) was generated by substituting adenosine (A) with cytosine (C) at the m6A site. HEK293T cells were co‐transfected with these reporter plasmids and a VIRMA expression vector or empty control using Lipofectamine 2000. Firefly and Renilla luciferase activities were measured 48 h post‐transfection using a Synergy Mx Multi‐Mode Microplate Reader (BioTek). Relative luciferase activity was calculated as the ratio of Firefly to Renilla activity.

### 4.11 RNA Immunoprecipitation (RIP) Assay

The RNA immunoprecipitation kit (Geneseed, Guangzhou, China) was used in accordance with the protocol to identify the connection between VIRMA and GATA3. In a nutshell, 1 × 10^7^ cells were lysed, and the supernatant was collected. IgG (Cell Signaling Technology, Beverly, MA, USA), VIRMA (NOVUS Biologicals, USA), or YTHDF1 (NOVUS Biologicals, USA) antibodies were then ligated on magnetic beads and mixed with the supernatant for antibody capture. Ultimately, purified RNA was obtained and detected by universal primers.

### 4.12 MeRIP‐qPCR

The TRIzol technique was used to isolate and purify total RNA. Using the Zymo RNA clean and concentrator‐5 kit, 20ug of total RNA from the collected samples was purified and recovered from the fragmented RNA after being interrupted at high temperature and processed to create the initial digested DNA. The m6A modification site on the RNA was reacted with using the m6A antibody(or IgG) by immunoprecipitation, and the RNA was then extracted once more using phenol chloroform lysate. Following reverse transcription into cDNA, qPCR tests were conducted. qPCR was used to determine the m6A enrichment, standardized by input, in each sample. Normalization of the relative enrichment was done to the input as: %Input = Fd × 2^Ct [IP or IgG] – Ct [input]^ × 100%. Fd is Input dilution factor.

### 4.13 Chromatin Immunoprecipitation (ChIP)‐qPCR

ChIP was performed using the Chromatin IP DNA Purification Kit (Active Motif, CA, USA). Lysates were immunoprecipitated with an anti‐H3K18la antibody to investigate its binding to the *VIRMA* promoter. The precipitated DNA was then analyzed by qPCR using site‐specific primers.

### 4.14 Bioinformatics and Dataset Analysis

Differential gene expression in lung fibrosis was analyzed using GEO datasets GSE24206 and GSE110147 (IPF), and GSE216376, GSE114142, and GSE222576 (kidney, intestinal, and liver fibrosis). Conserved lactylation sites on histone H3 were predicted using CPLM (https://cplm.biocuckoo.cn/) and UniProt databases (https://www.uniprot.org/). H3K18la enrichment patterns at the *VIRMA* promoter were visualized using ChIP‐seq datasets GSE171832 and GSE156675.

### 4.15 Statistical Analysis

Data are reported as mean ± SD. Statistical significance was established with a two‐tailed Student's *t*‐test for two‐group comparisons and a one‐way ANOVA for multiple groups, followed by post‐hoc tests. Analysis was performed using SPSS version 24.0 (SPSS Inc., Chicago, USA). *p* value < 0.05 was considered statistically significant.

## Author Contributions

Conceptualization: S.J. and W.W.; Methodology: J.Y. and M.W.; Validation, J.Y., M.W. and D.Y.; Formal analysis, W.X.; S.L. and M.Z.; Data curation, X.H.; Writing – original draft preparation, J.Y.; Writing – review and editing, J.Y. and W.W.; supervision, S.J.; funding acquisition, S.J. and W.W.

## Ethics Statement

All procedures in this study were approved by the Ethics Committee of Jilin University on 10 February 2022 (approval No. (2022‐02‐10)). Animal experiments methods were performed in accordance with the relevant guidelines and regulations.

## Conflicts of Interest

The authors declare no conflicts of interest.

## Supporting information




**Supporting File 1**: advs77328‐sup‐0001‐SuppMat.docx.

**Supporting File 2**: advs77328‐sup‐0002‐Figures.docx.

**Supporting File 3**: advs77328‐sup‐0003‐Tables.docx.

**Supporting File 4**: advs77328‐sup‐0004‐SuppMat.dna.

**Supporting File 5**: advs77328‐sup‐0005‐original‐data.pdf.

**Supporting File 6**: advs77328‐sup‐0006‐original‐data.xlsx.

## Data Availability

The data that support the findings of this study are available from the corresponding author upon reasonable request.
