# Supplementary material for: p300‐Mediated H3K18 Lactylation Drives Radiation‐Induced Pulmonary Fibrosis via VIRMA‐Dependent m6A Modification of GATA3
Source: Adv Sci (Weinh). 2026 Aug 24;13(63):e77328. doi: 10.1002/advs.77328 (PMC13502275; doi:10.1002/advs.77328)
Supplement: Supplementary file 1 [file ADVS-13-e77328-s004.pdf]

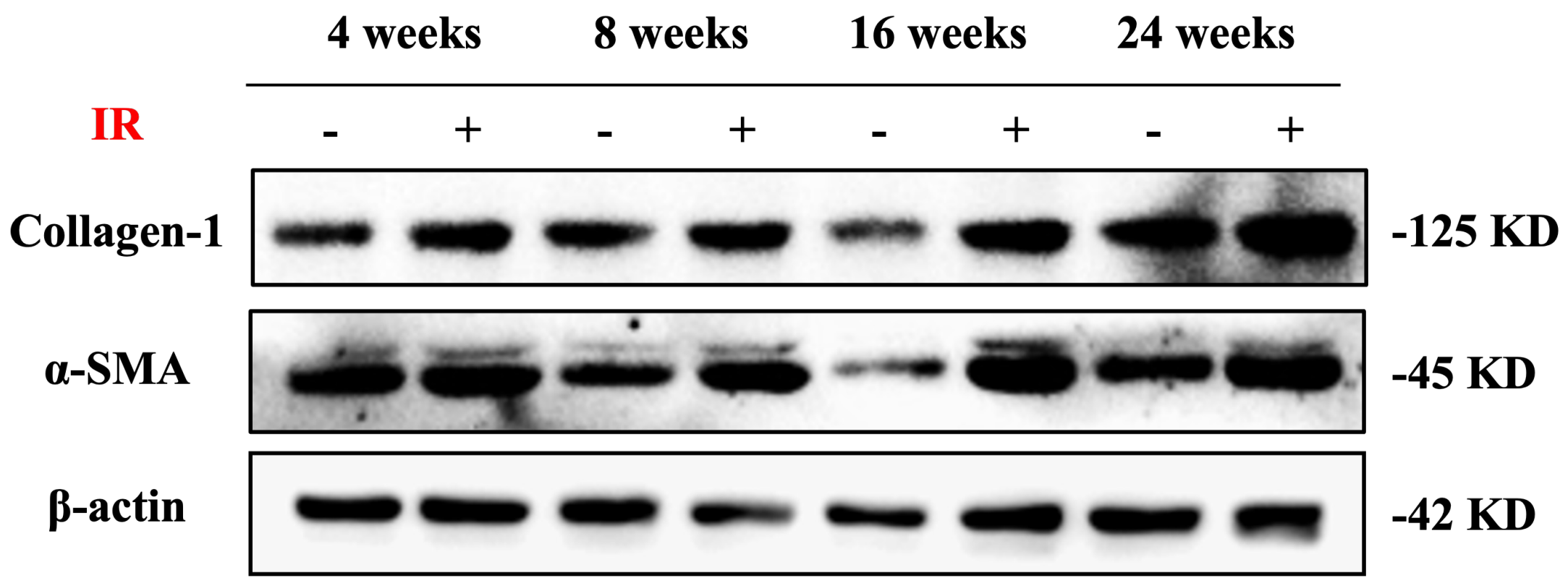

Collagen-1

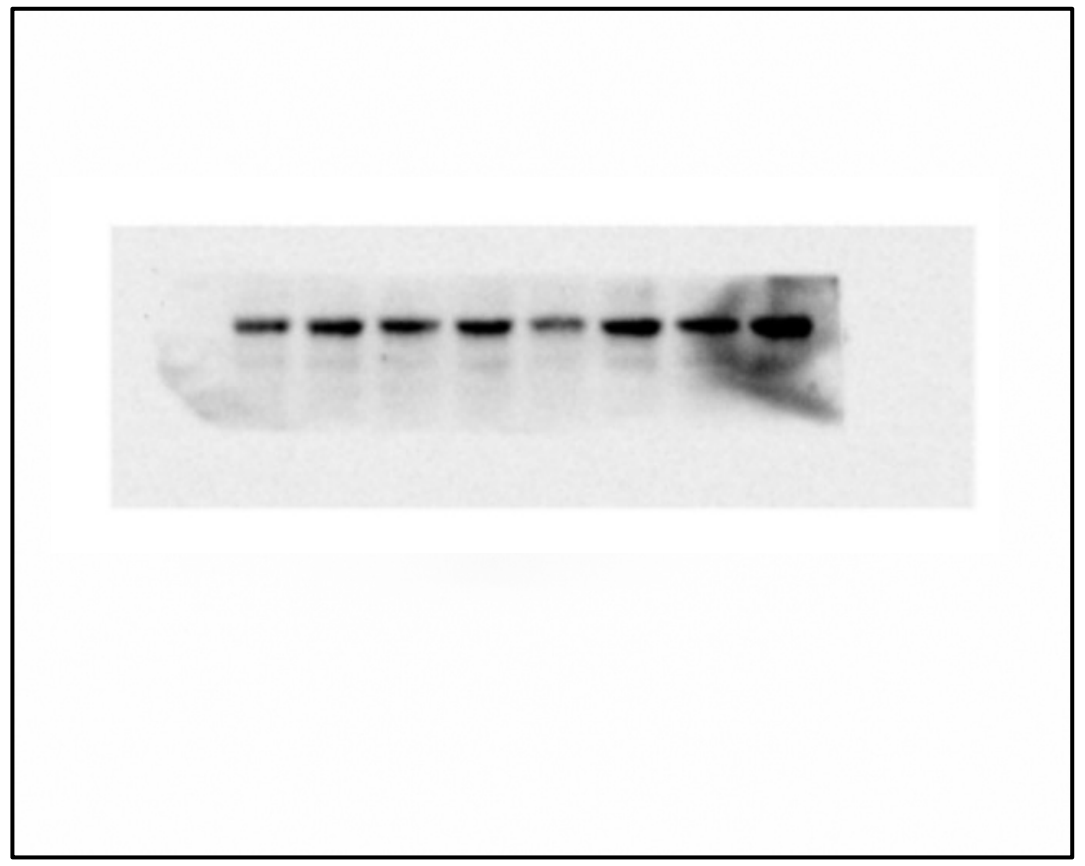

α-SMA

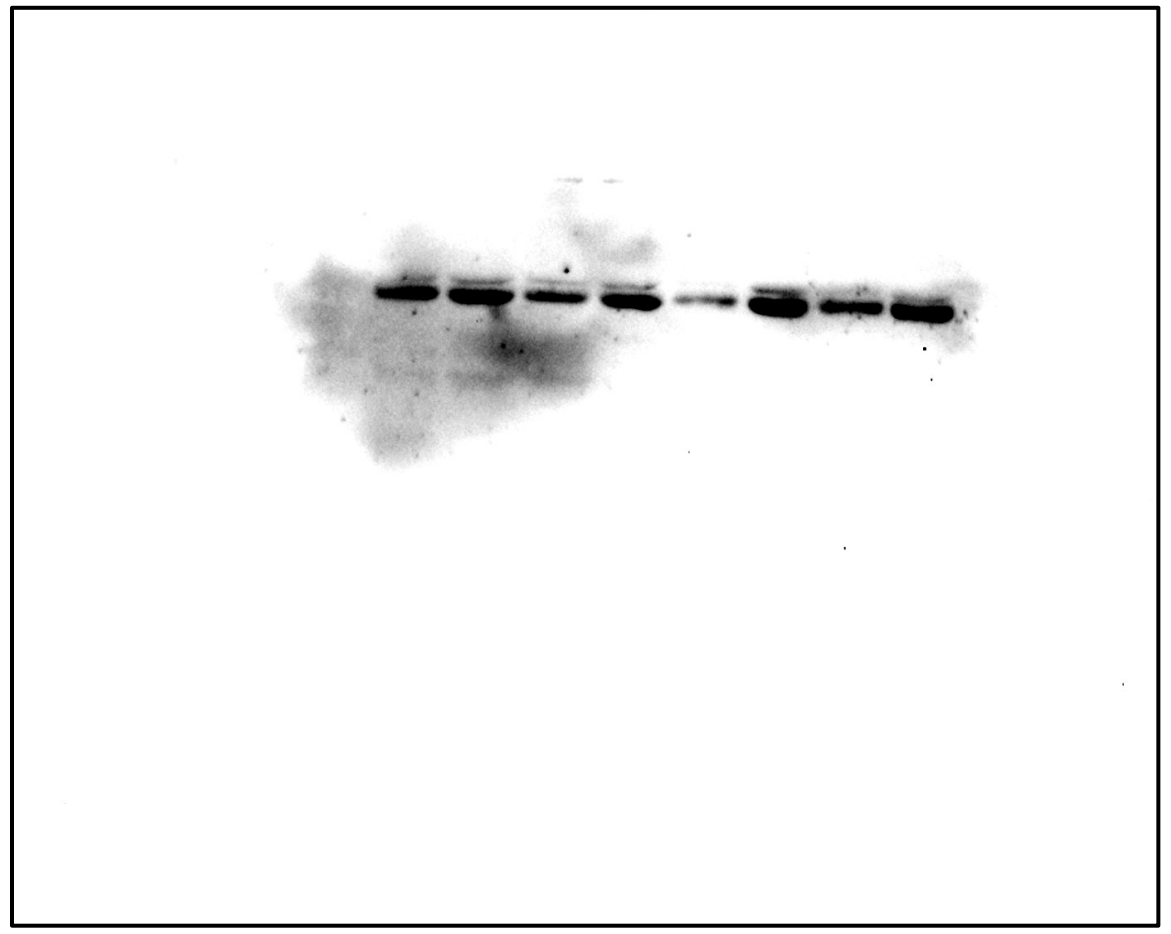

β-actin

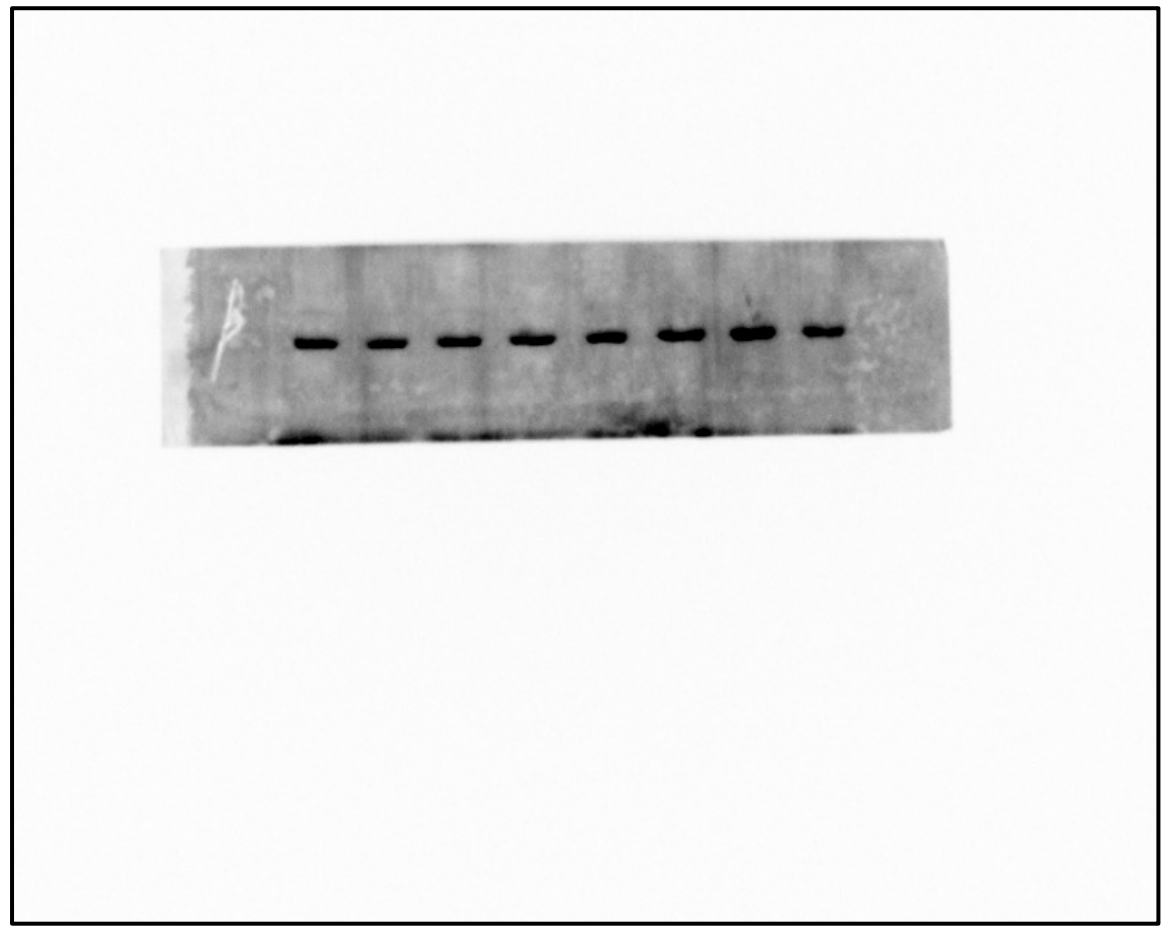

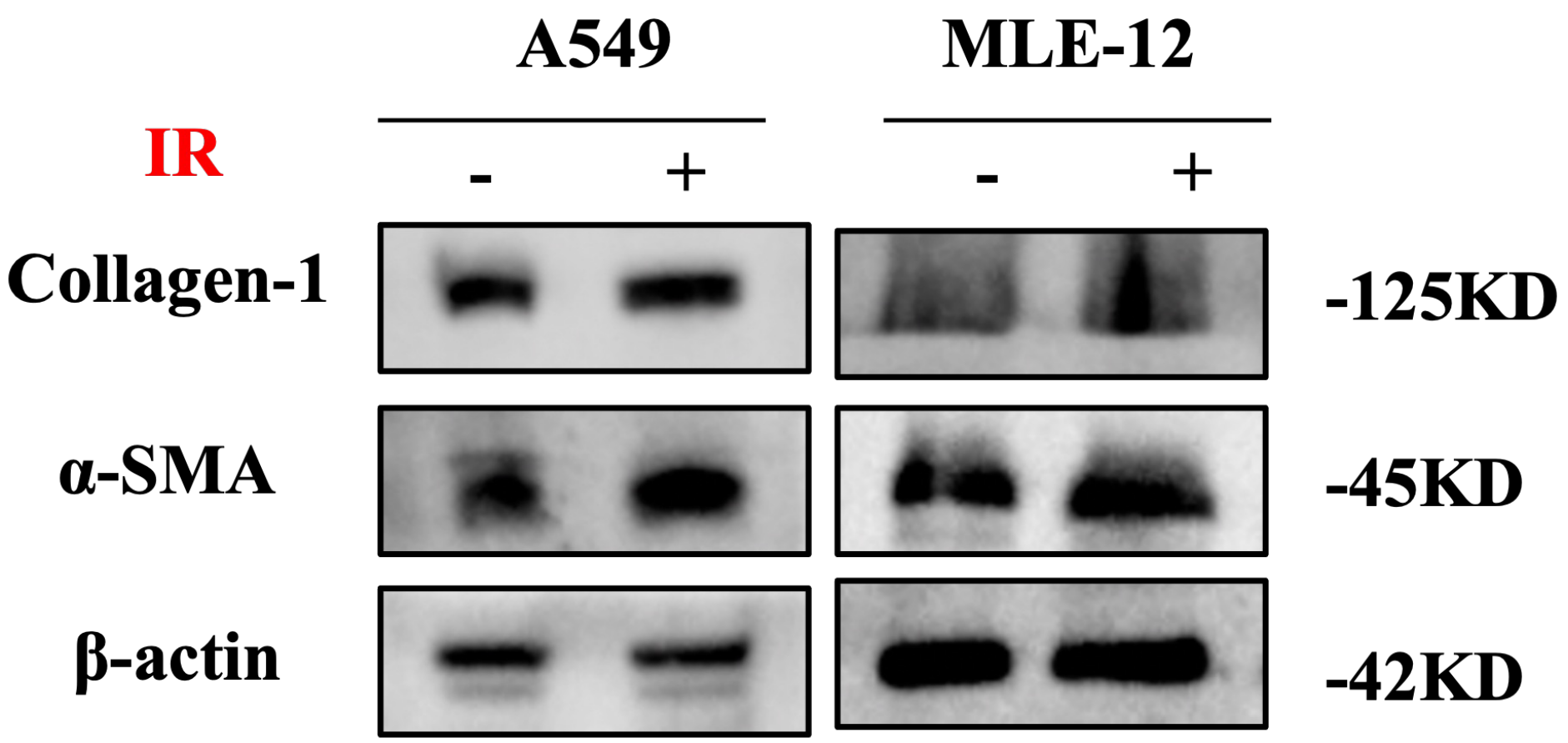
A549
MLE-12
IR
-
+
-
+
Collagen-1
α-SMA
β-actin
-125KD
-45KD
-42KD

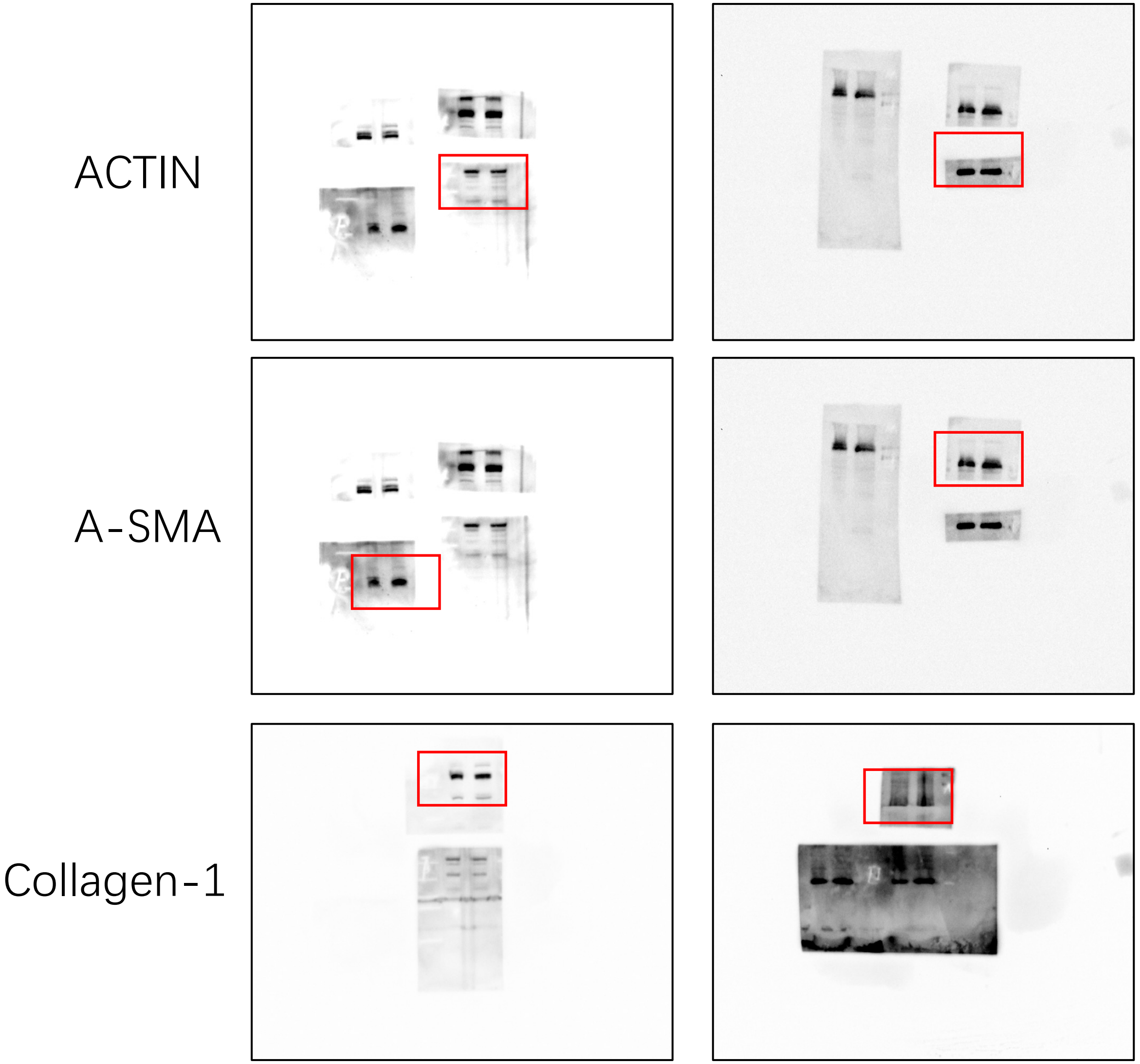
ACTIN
A-SMA
Collagen-1

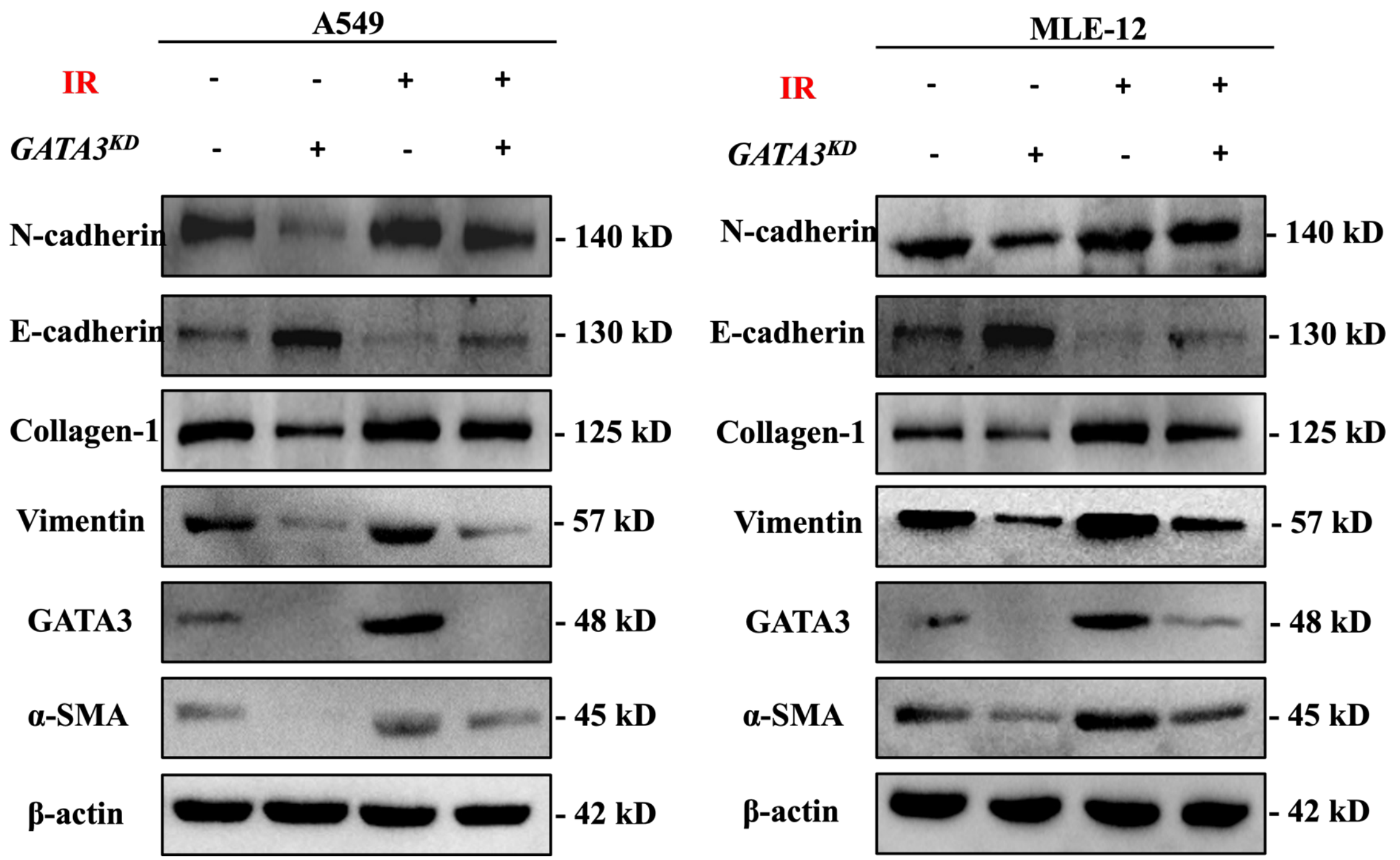
A549
MLE-12
IR - - + +
GATA3KD - + - +
N-cadherin - 140 kD
E-cadherin - 130 kD
Collagen-1 - 125 kD
Vimentin - 57 kD
GATA3 - 48 kD
α-SMA - 45 kD
β-actin - 42 kD

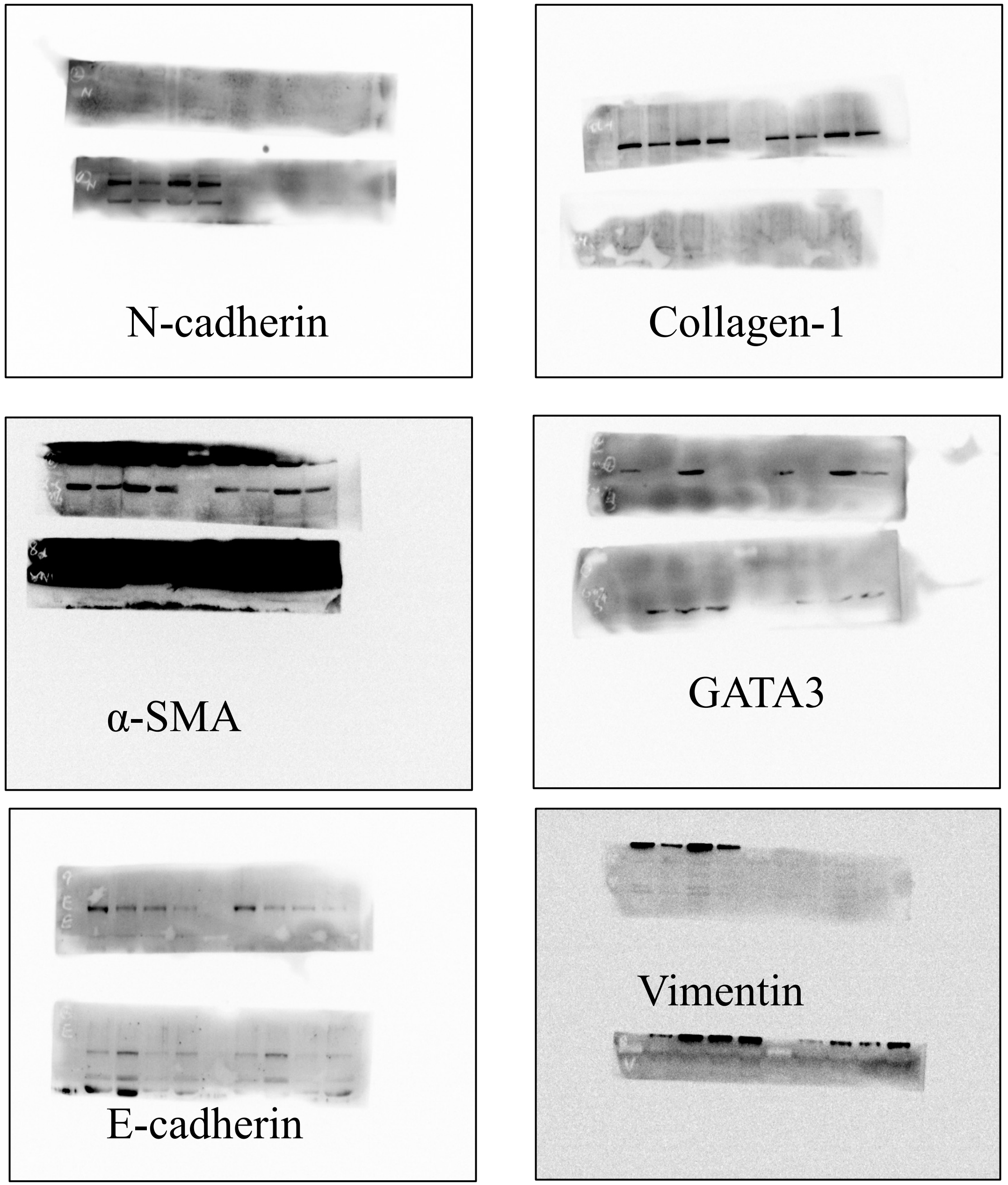
N-cadherin
Collagen-1
α-SMA
GATA3
E-cadherin
Vimentin

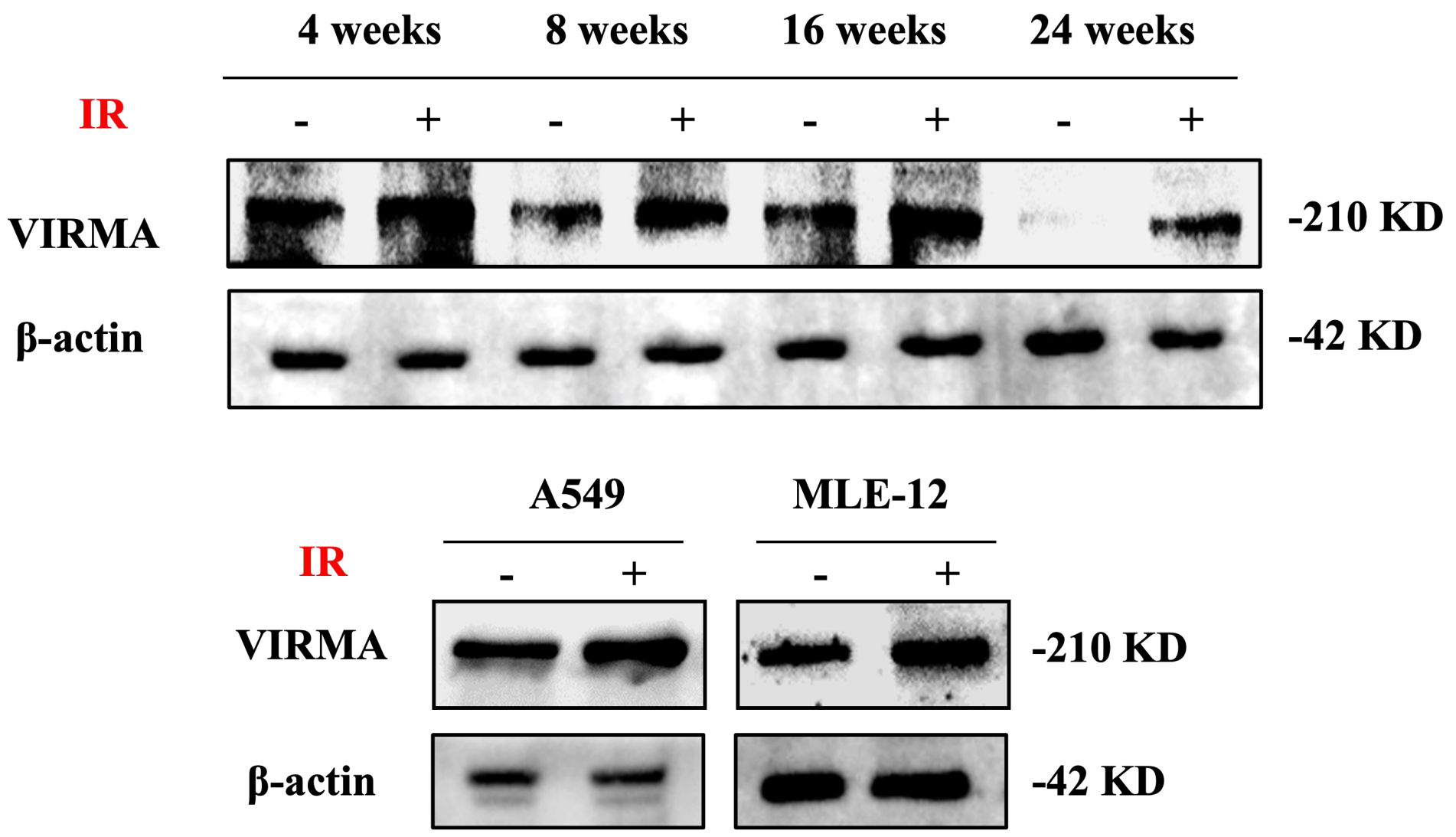
4 weeks
8 weeks
16 weeks
24 weeks
IR
- + - + - + - +
VIRMA
-210 KD
β-actin
-42 KD
A549
MLE-12
IR
- + - +
VIRMA
-210 KD
β-actin
-42 KD

ACTIN

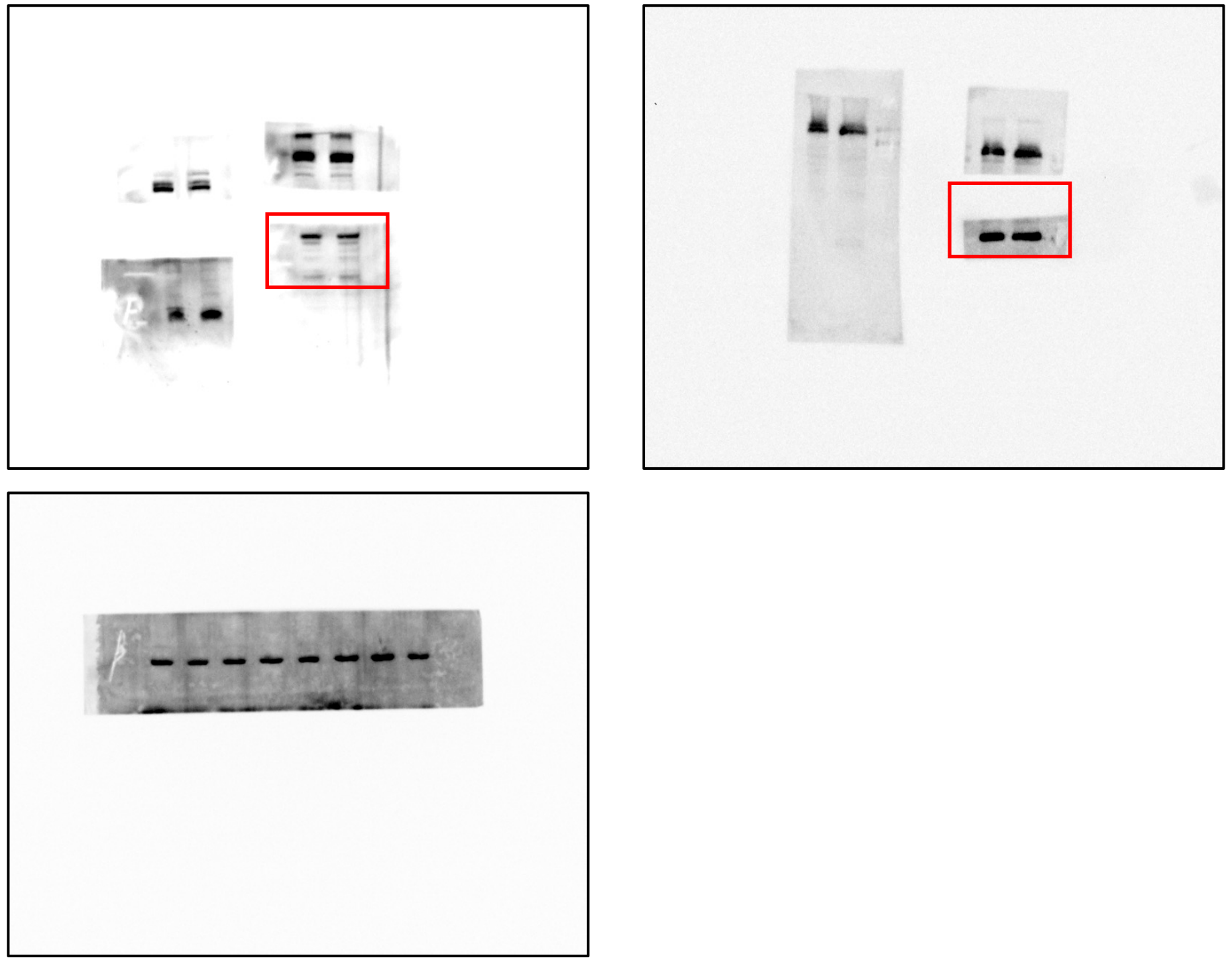

VIRMA

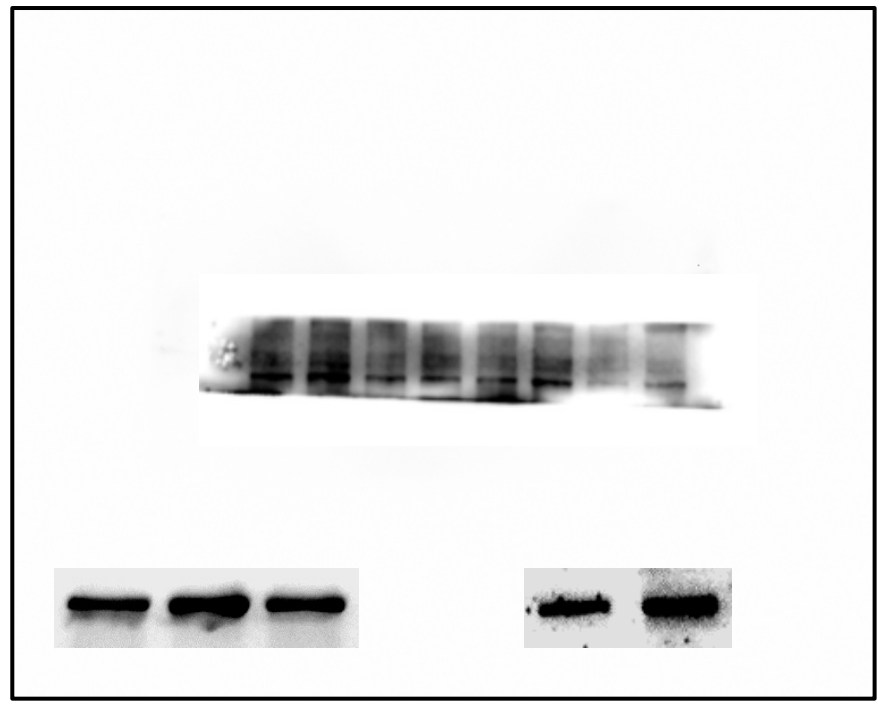

E

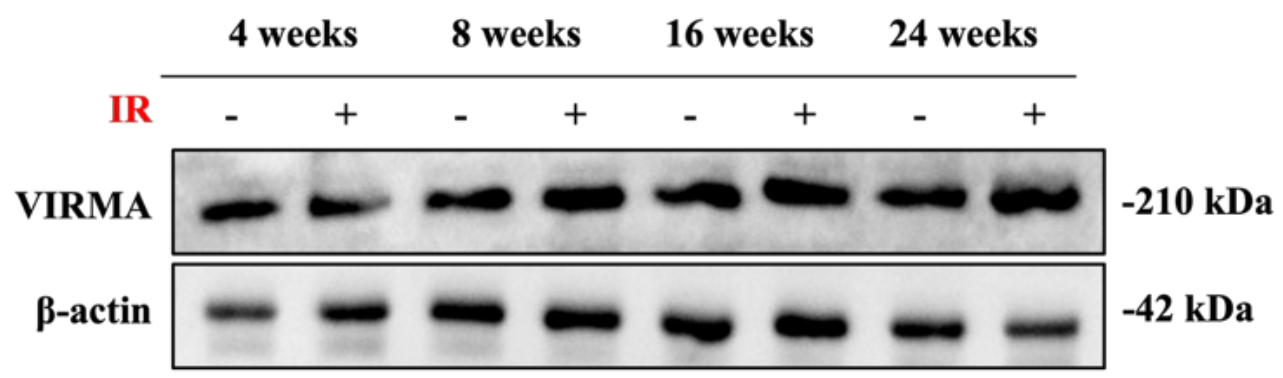

F

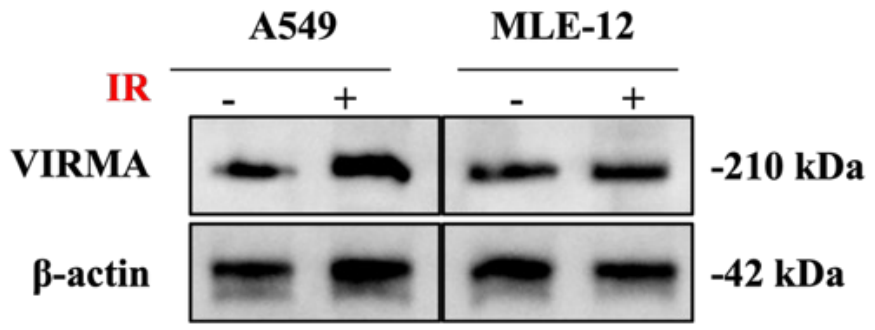

ACTIN

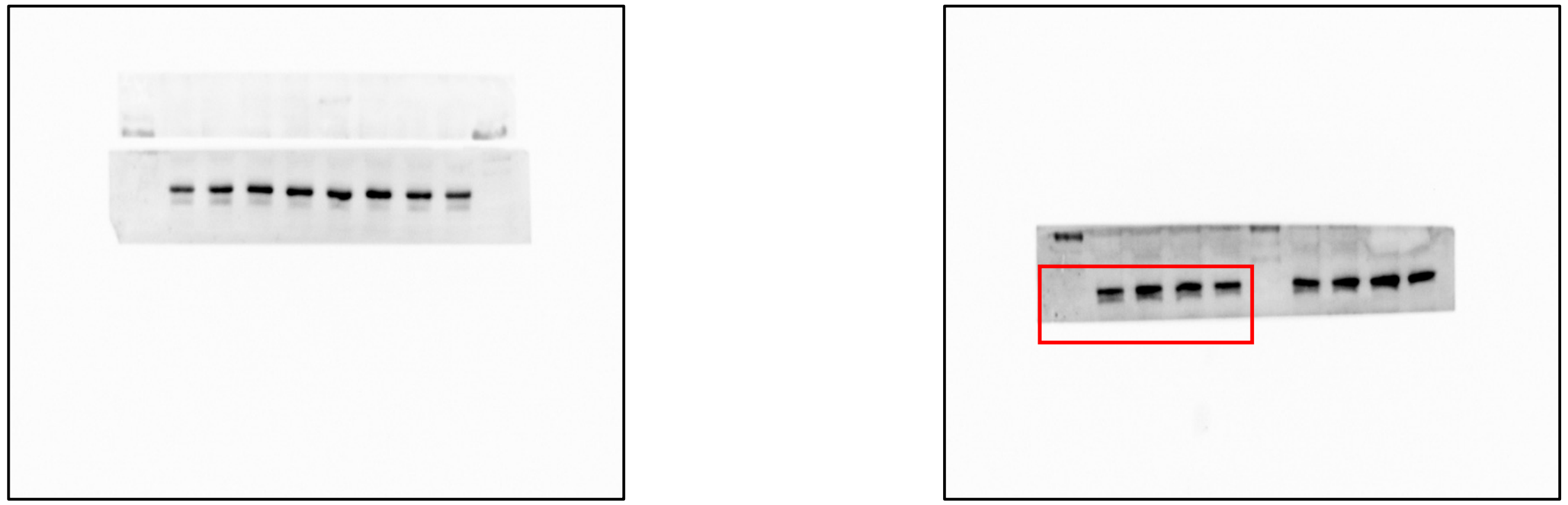

VIRMA

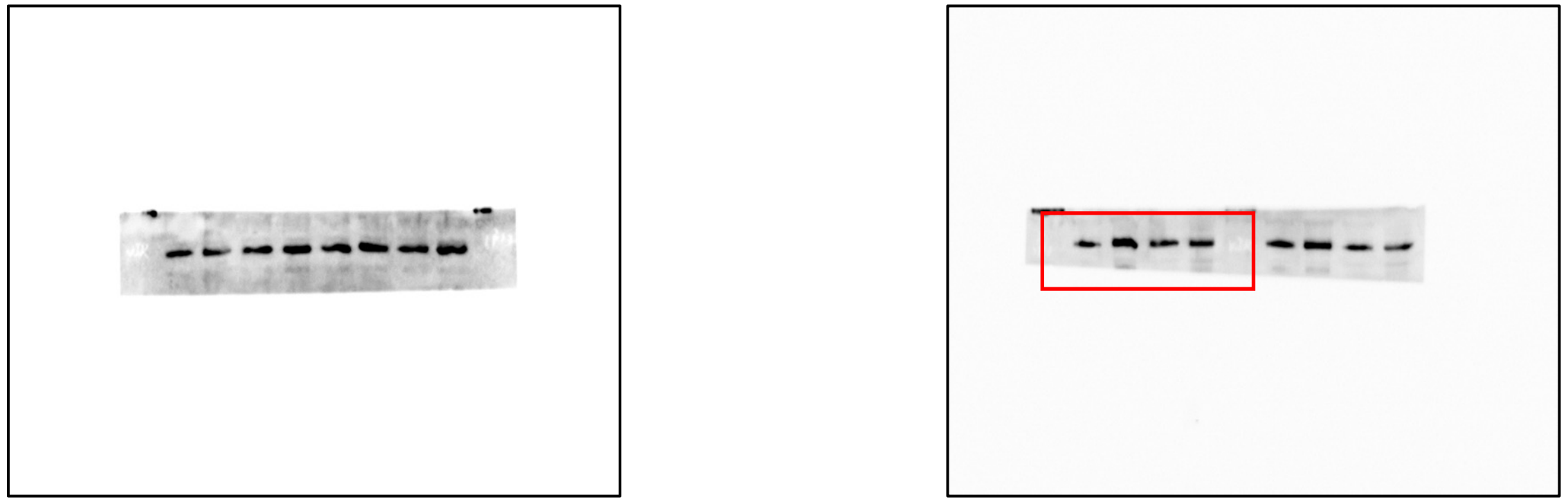

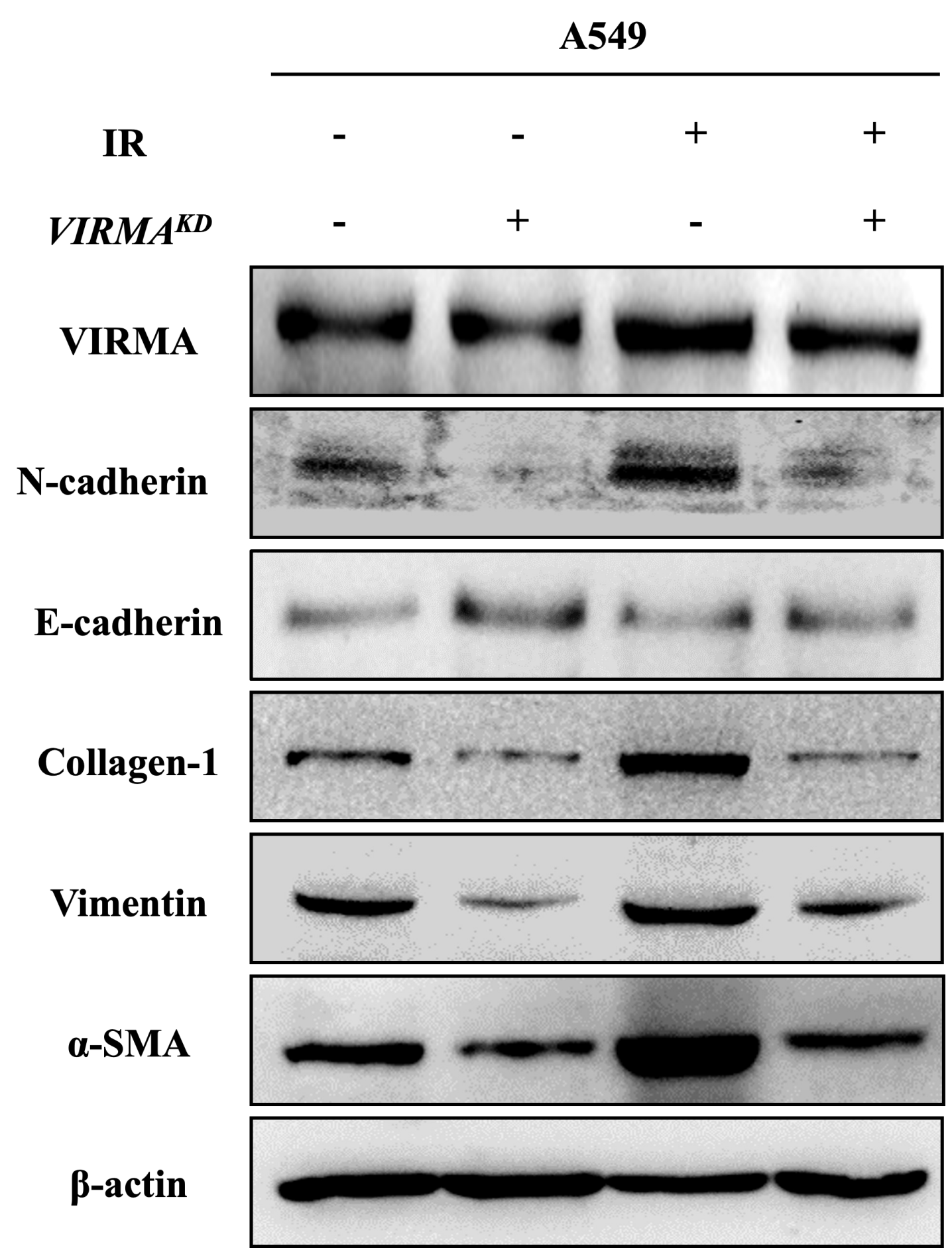
A549
IR - - + +
VIRMA^KD - + - +
VIRMA
N-cadherin
E-cadherin
Collagen-1
Vimentin
α-SMA
β-actin

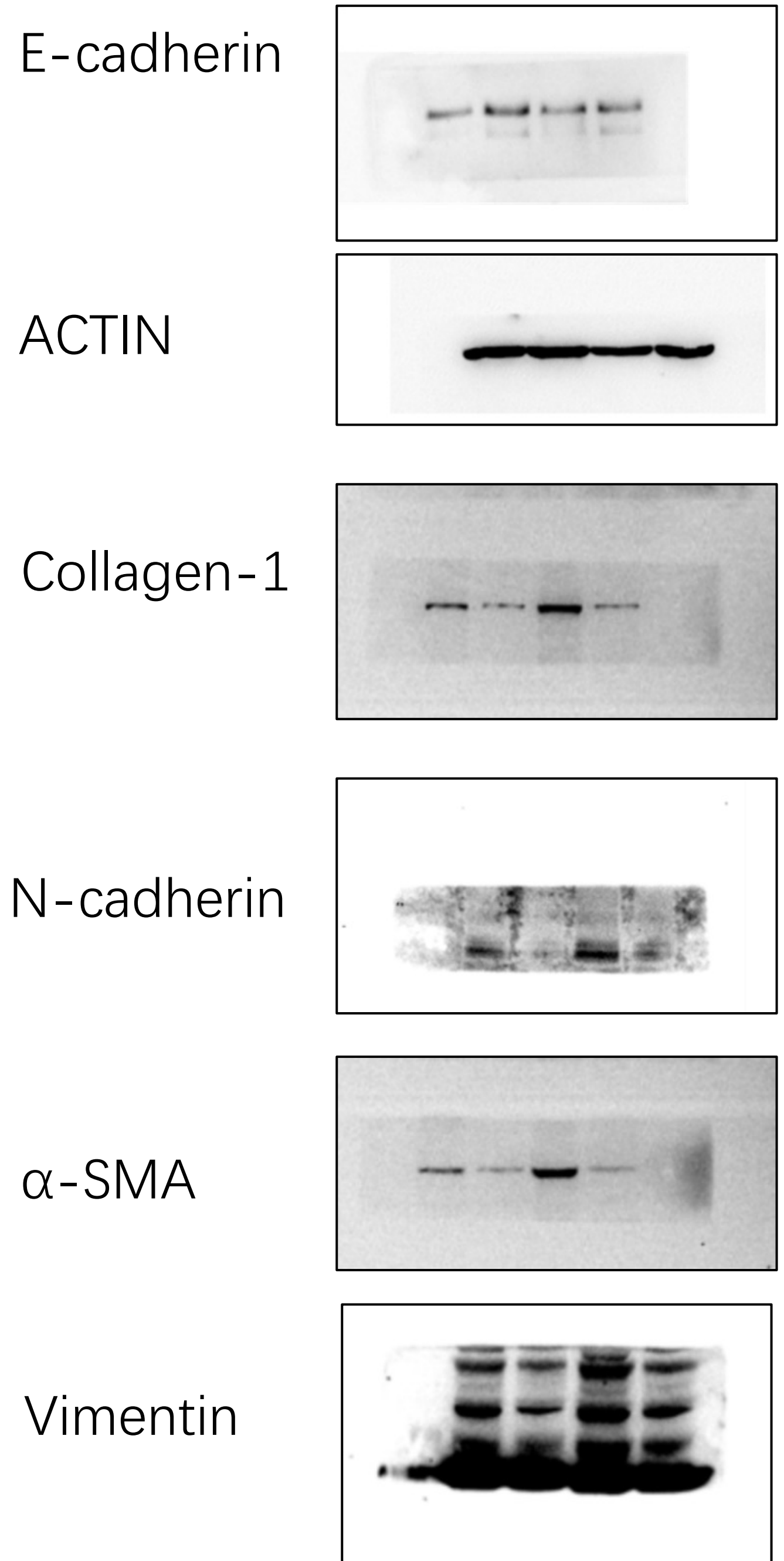
E-cadherin
ACTIN
Collagen-1
N-cadherin
α-SMA
Vimentin

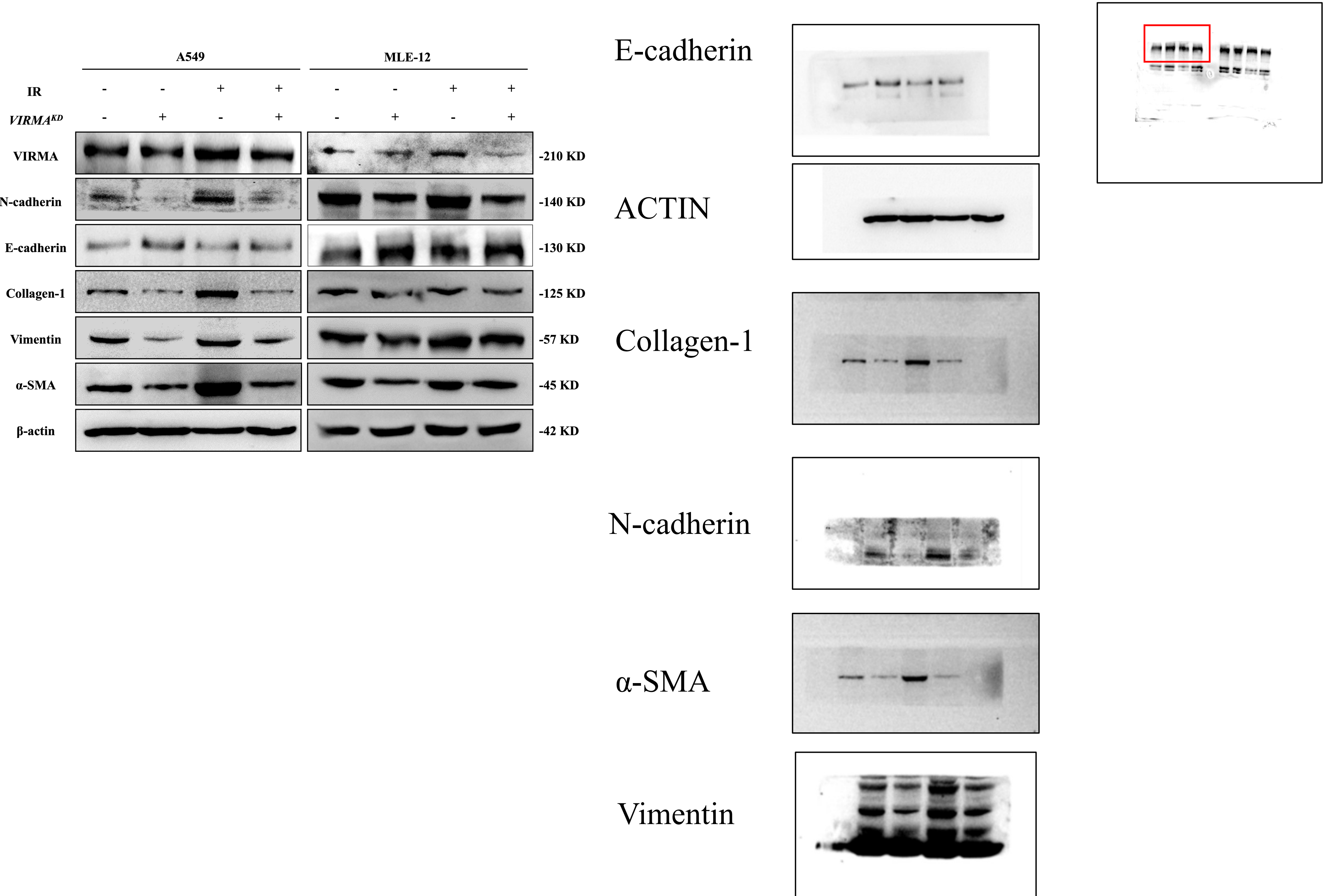
A549
MLE-12
IR
VIRMA^KD
VIRMA
N-cadherin
E-cadherin
Collagen-1
Vimentin
α-SMA
β-actin
-210 KD
-140 KD
-130 KD
-125 KD
-57 KD
-45 KD
-42 KD
E-cadherin
ACTIN
Collagen-1
N-cadherin
α-SMA
Vimentin

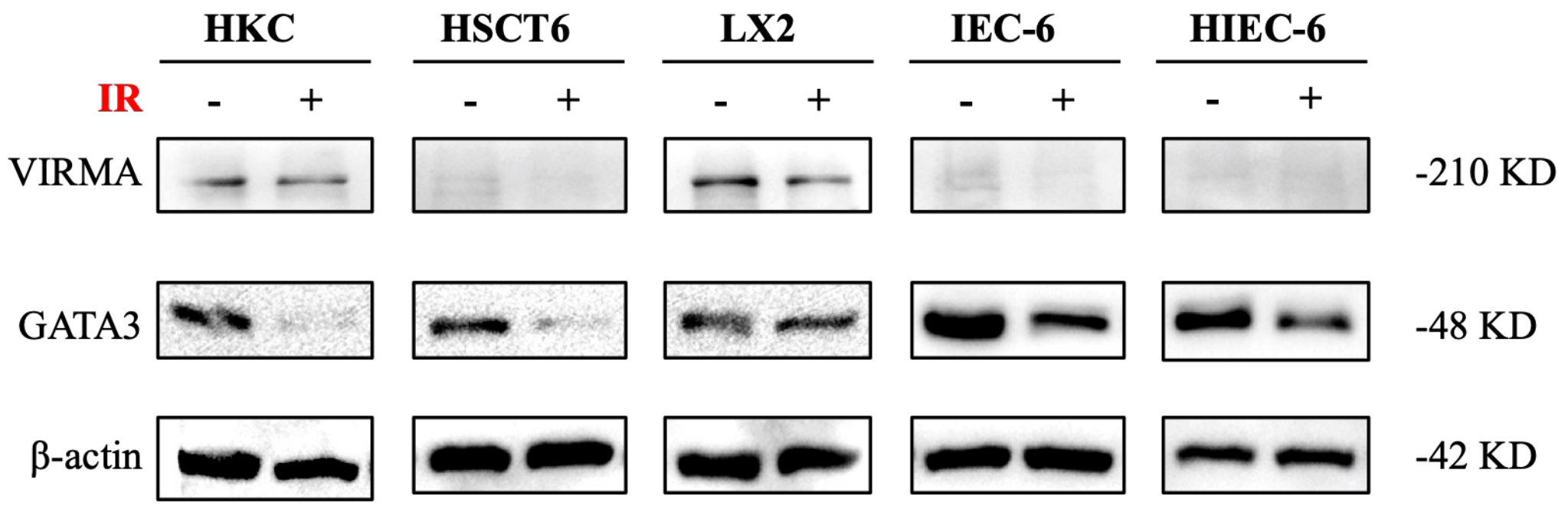
HKC
HSCT6
LX2
IEC-6
HIEC-6
IR - + - + - + - + - +
VIRMA
GATA3
β-actin
-210 KD
-48 KD
-42 KD

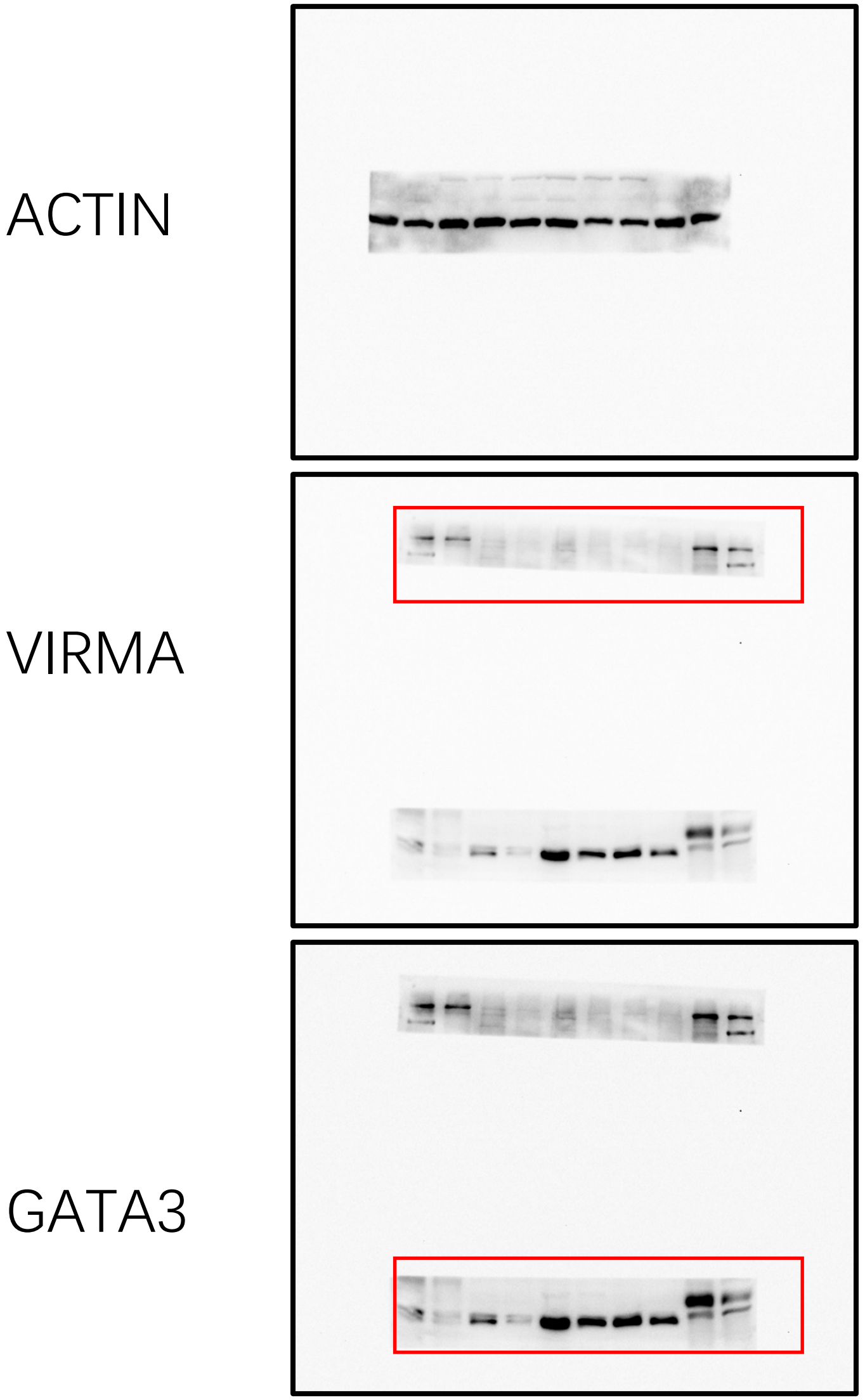
ACTIN
VIRMA
GATA3

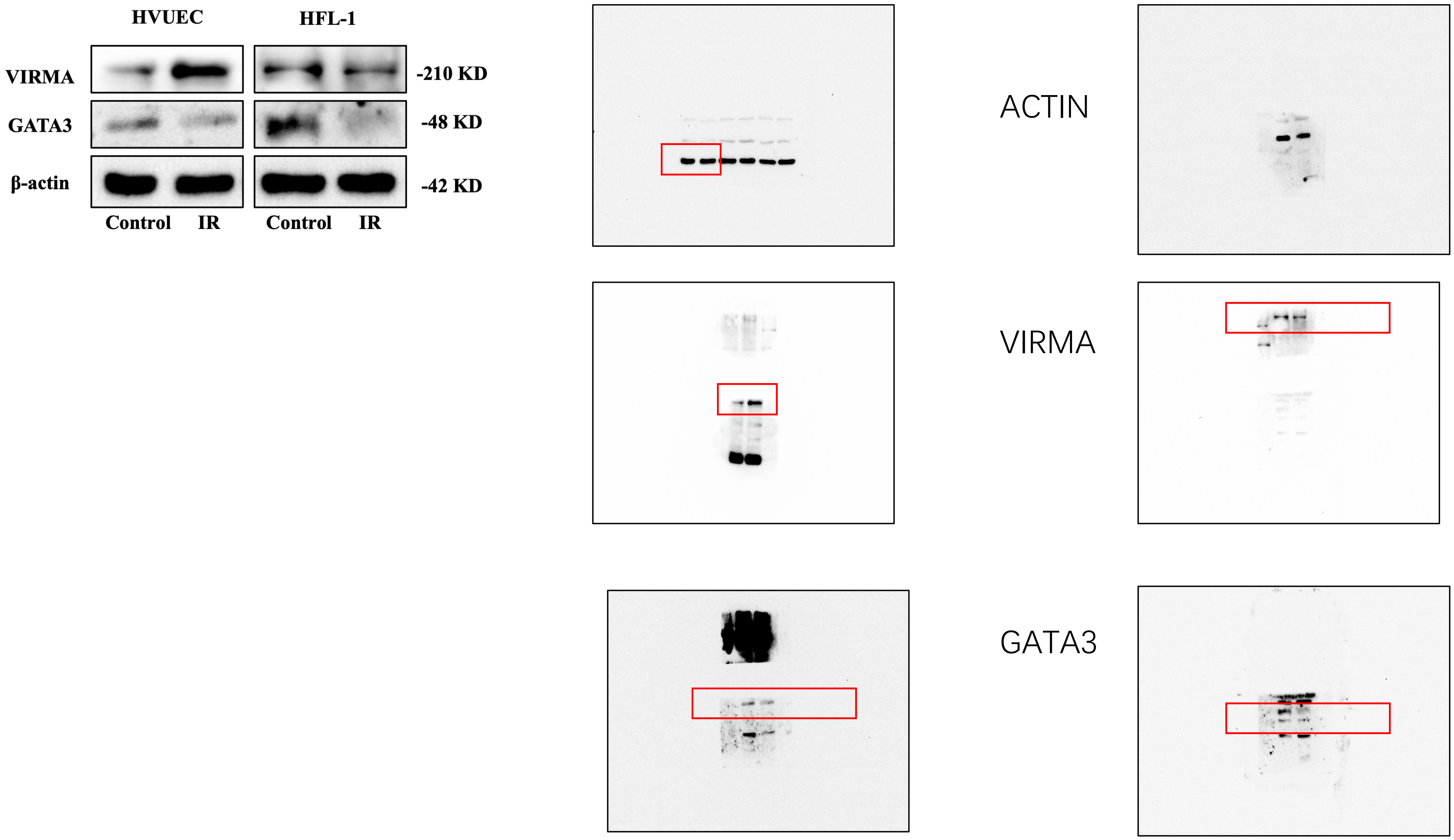
HVUEC
HFL-1
VIRMA
GATA3
β-actin
-210 KD
-48 KD
-42 KD
Control
IR
Control
IR
ACTIN
VIRMA
GATA3

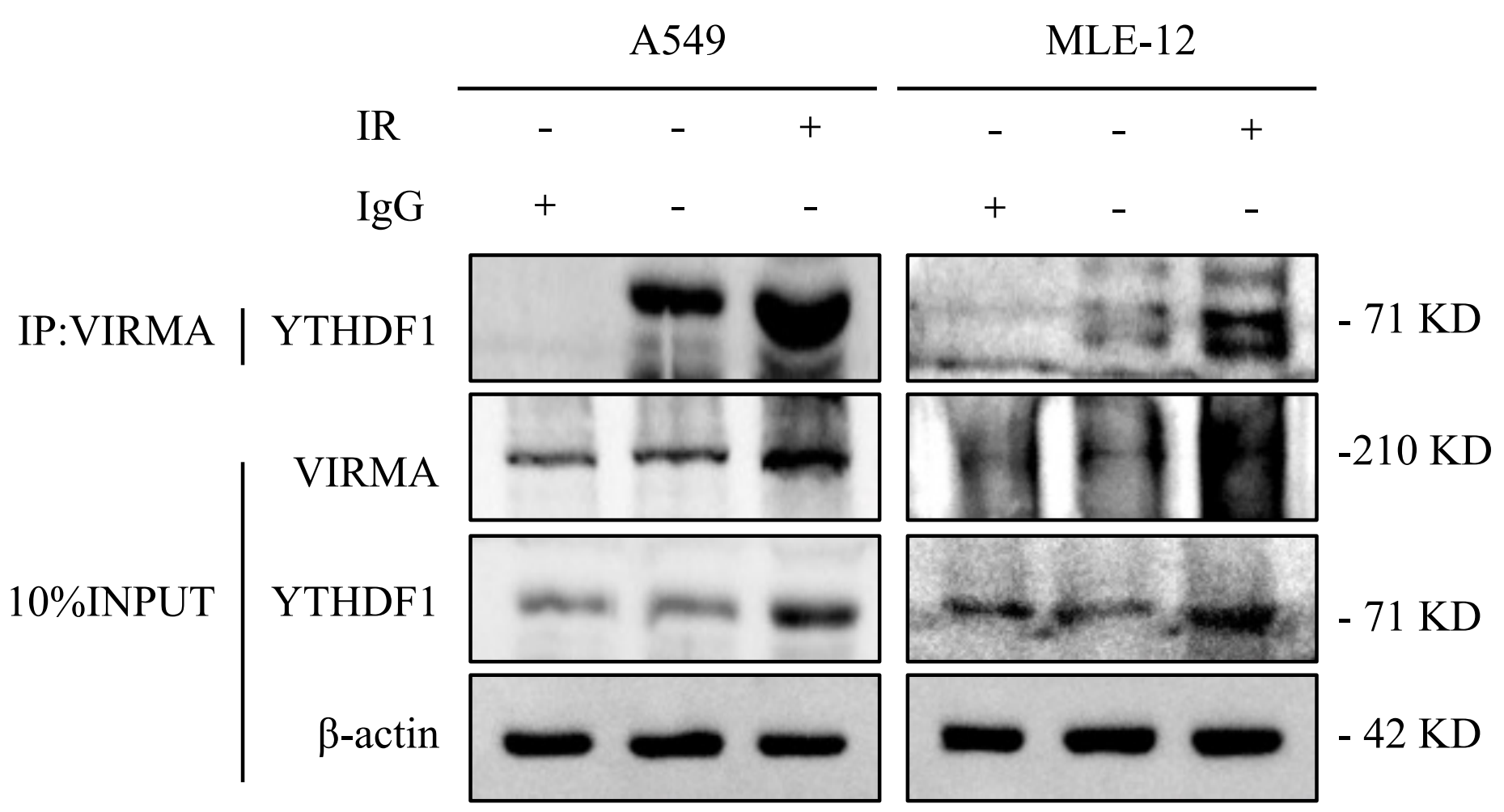

IP:YTHDF1

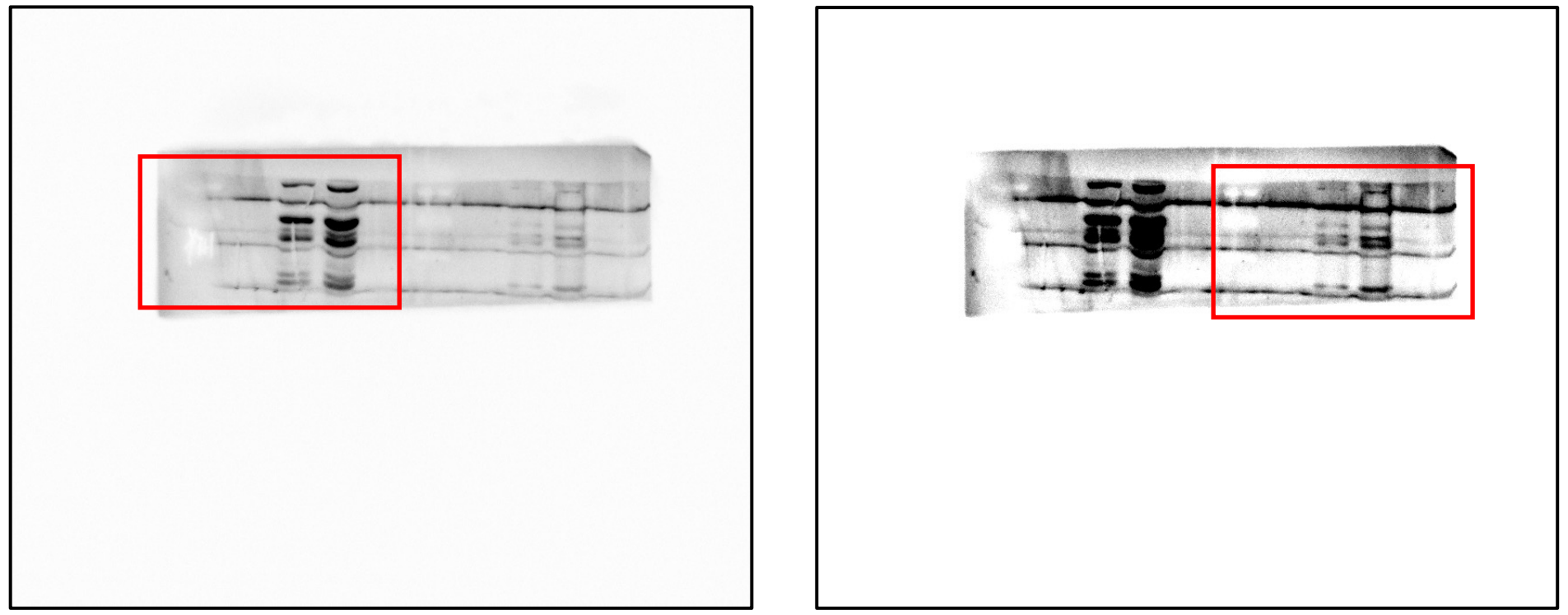

β-actin

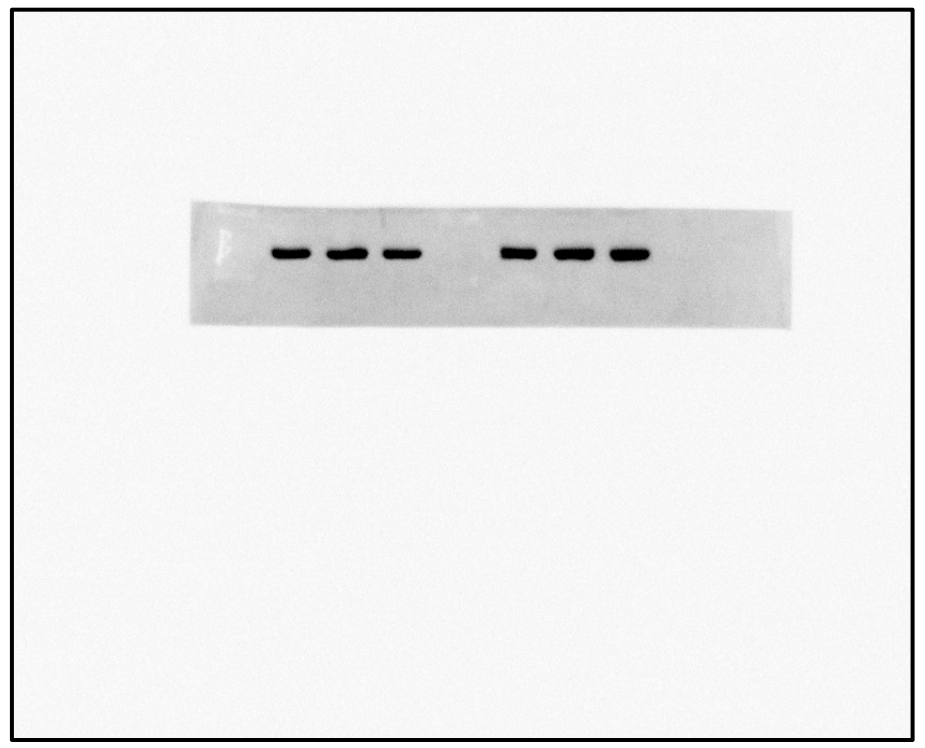

VIRMA

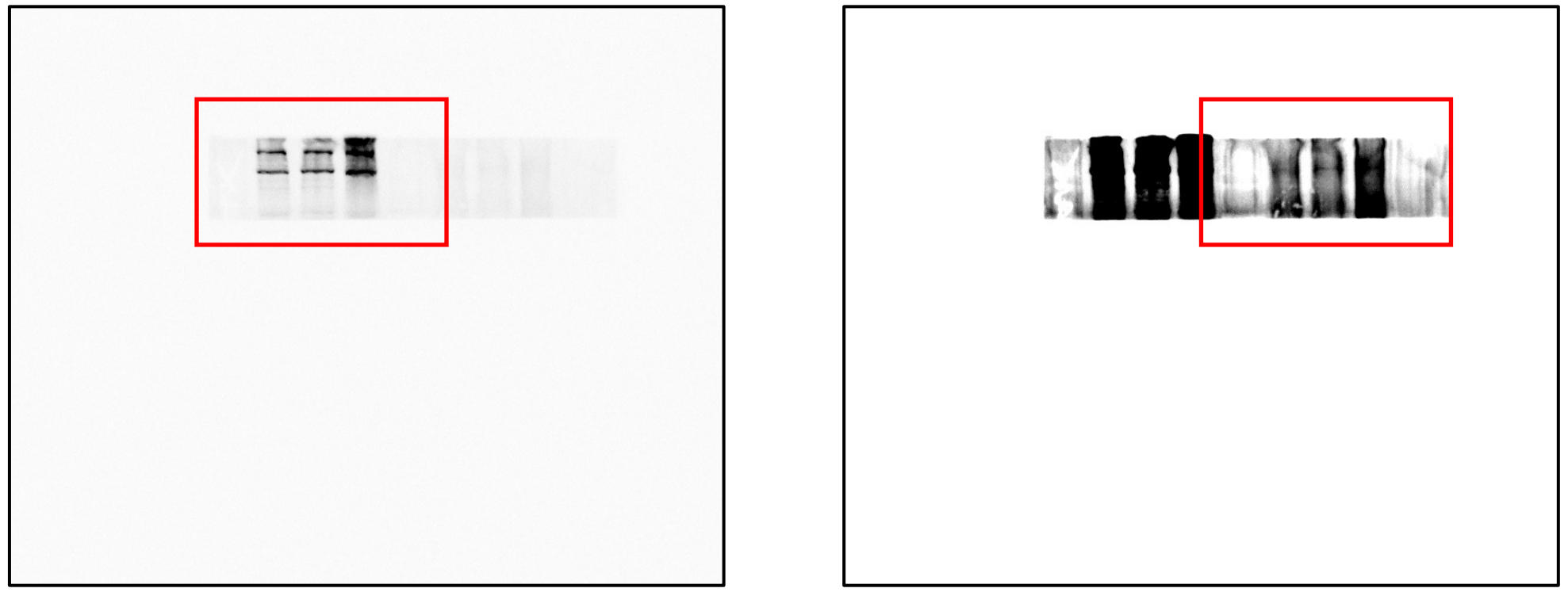

YTHDF1

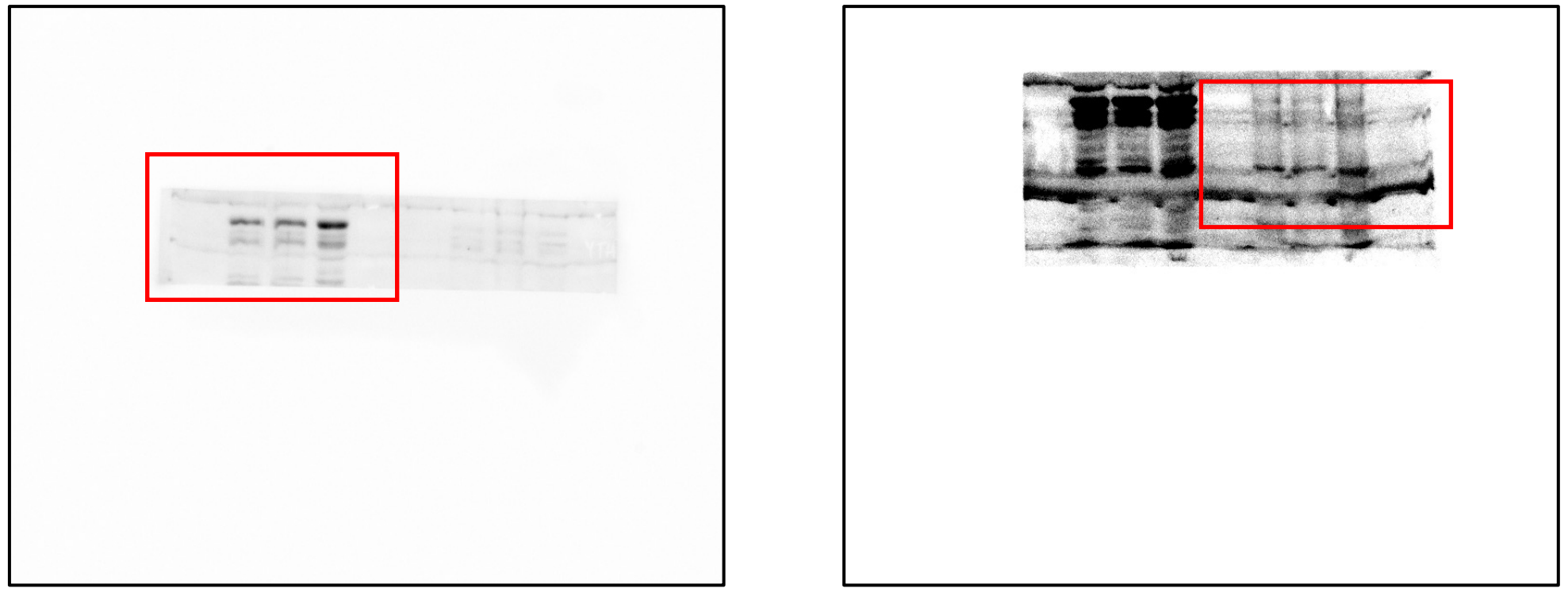

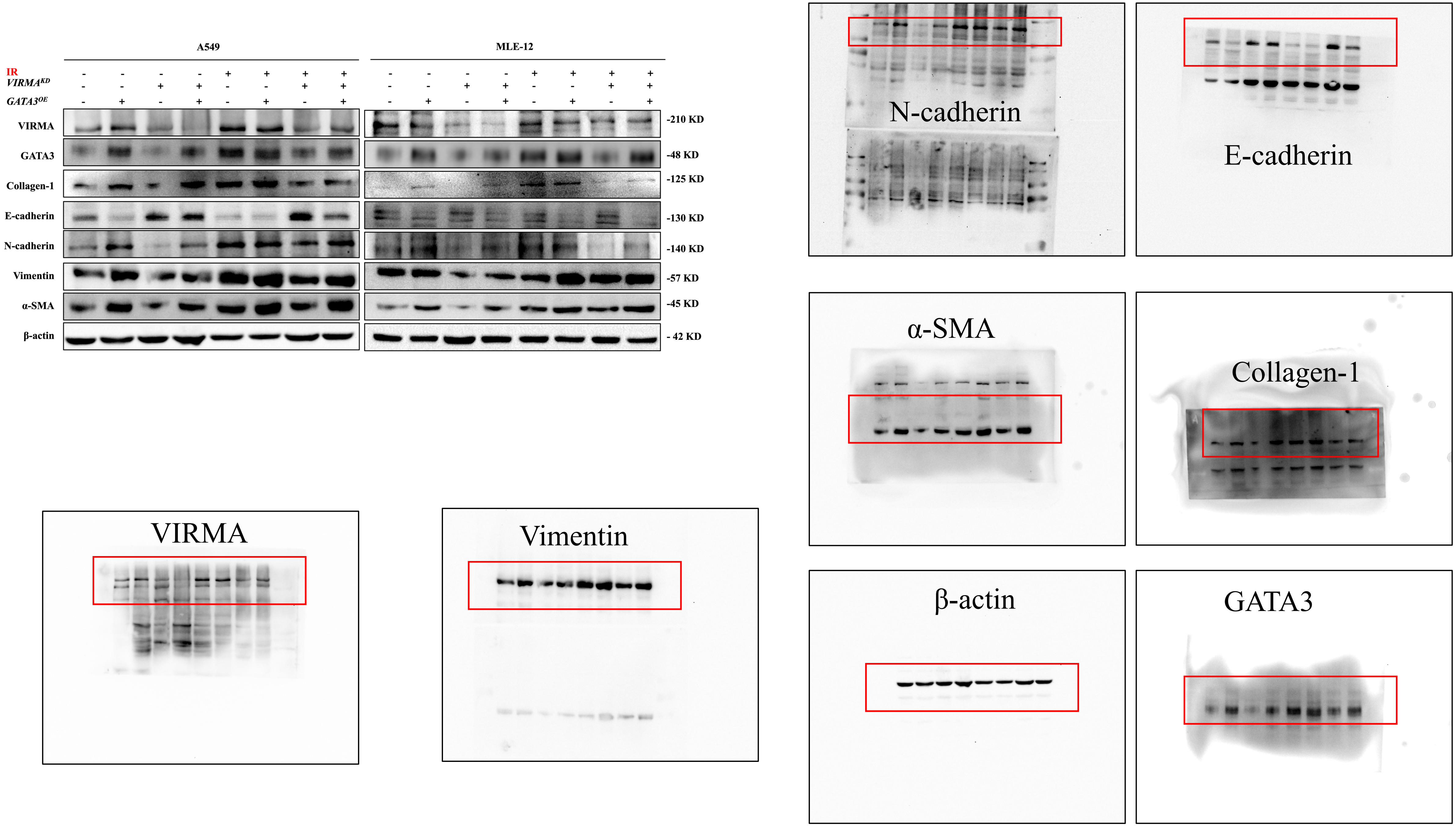
A549
MLE-12
IR
VIRMA^KD
GATA3^OE
VIRMA
GATA3
Collagen-1
E-cadherin
N-cadherin
Vimentin
α-SMA
β-actin
-210 KD
-48 KD
-125 KD
-130 KD
-140 KD
-57 KD
-45 KD
- 42 KD
N-cadherin
E-cadherin
α-SMA
Collagen-1
VIRMA
Vimentin
β-actin
GATA3

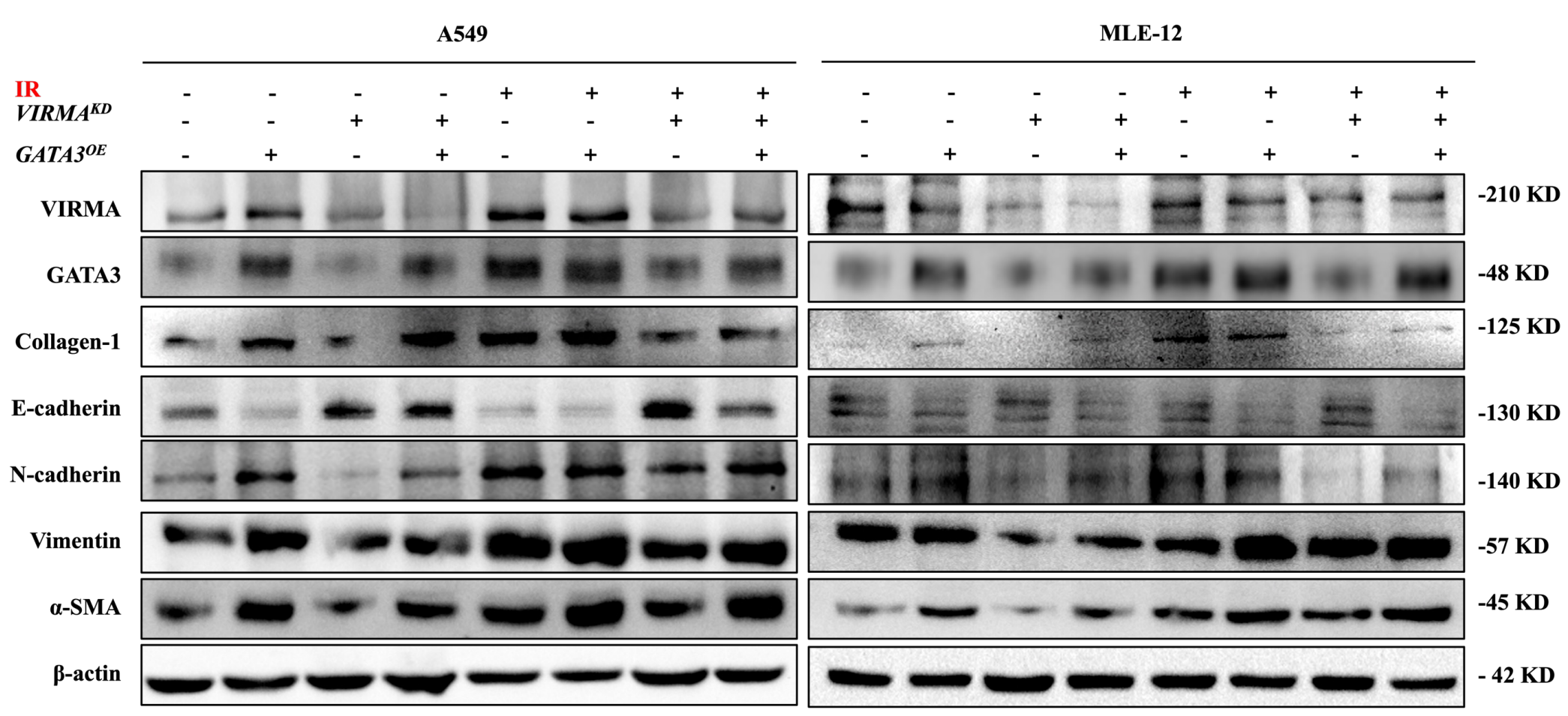
A549
MLE-12
IR
VIRMA^KD
GATA3^OE
VIRMA
GATA3
Collagen-1
E-cadherin
N-cadherin
Vimentin
α-SMA
β-actin
-210 KD
-48 KD
-125 KD
-130 KD
-140 KD
-57 KD
-45 KD
- 42 KD

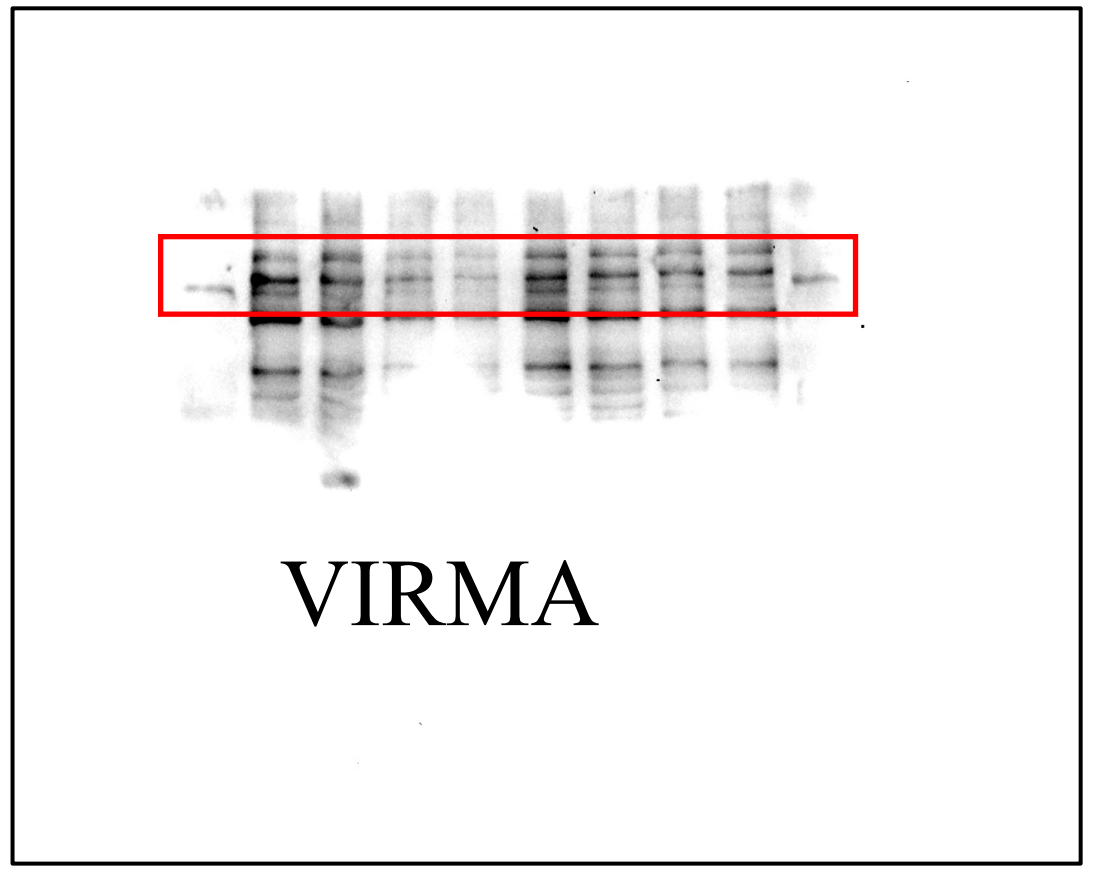
VIRMA

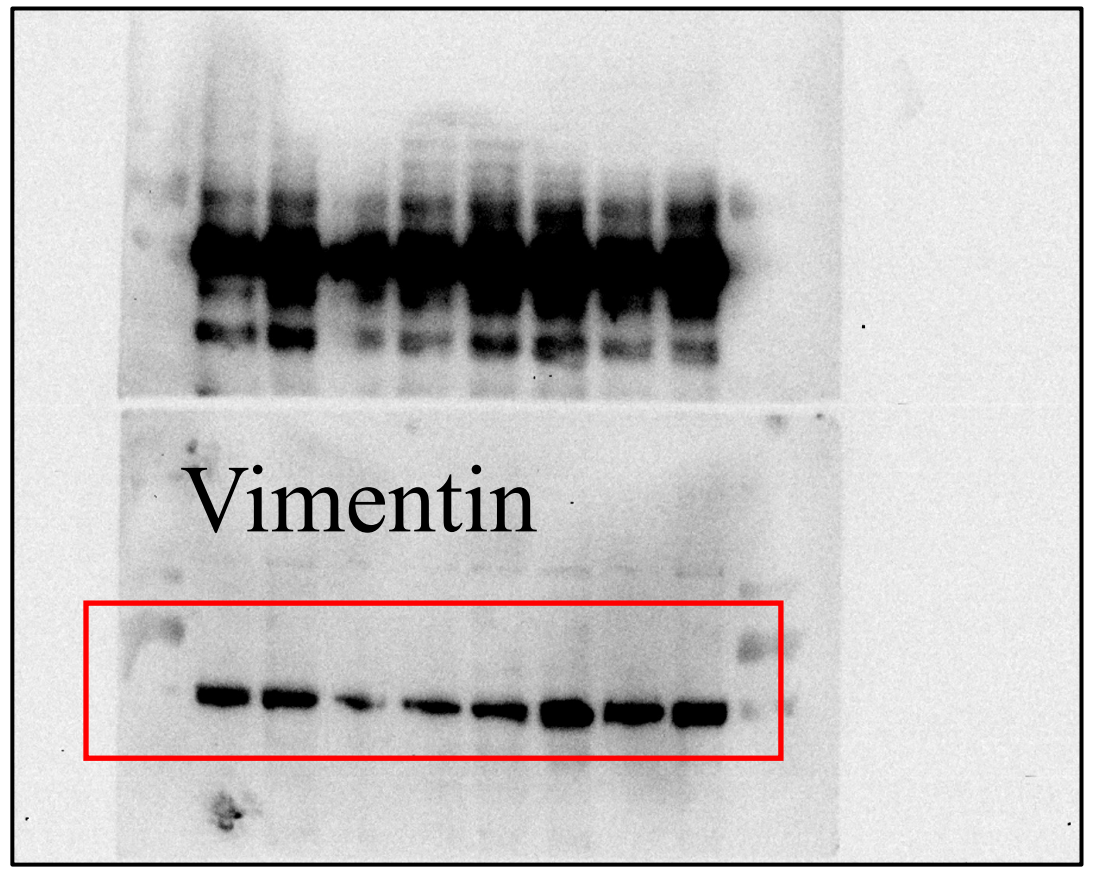
Vimentin

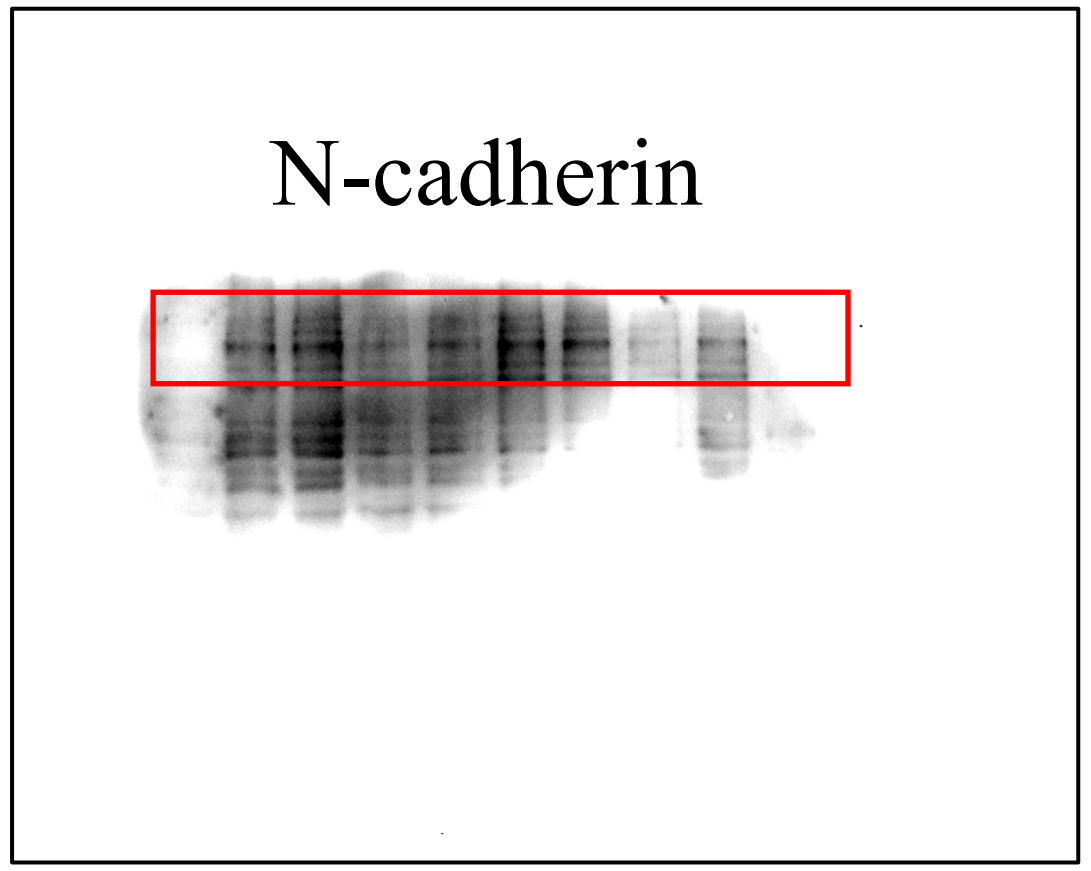
N-cadherin

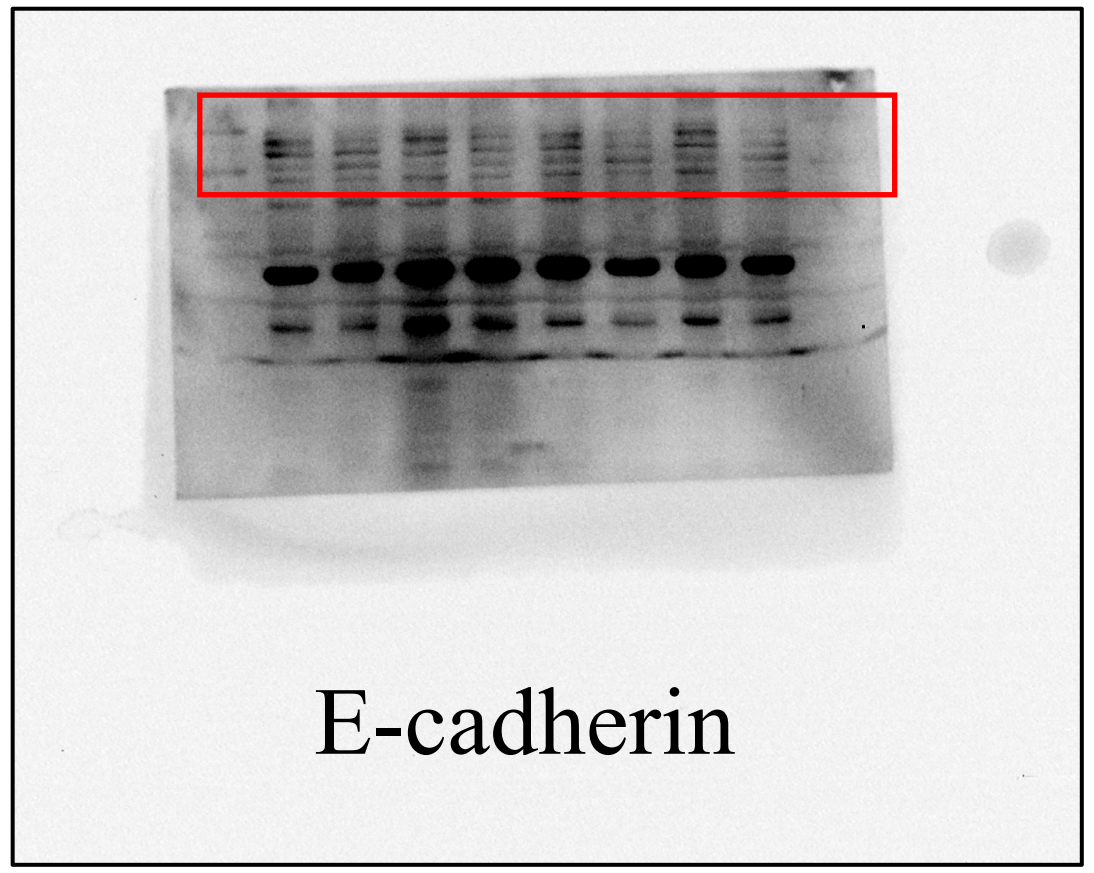
E-cadherin

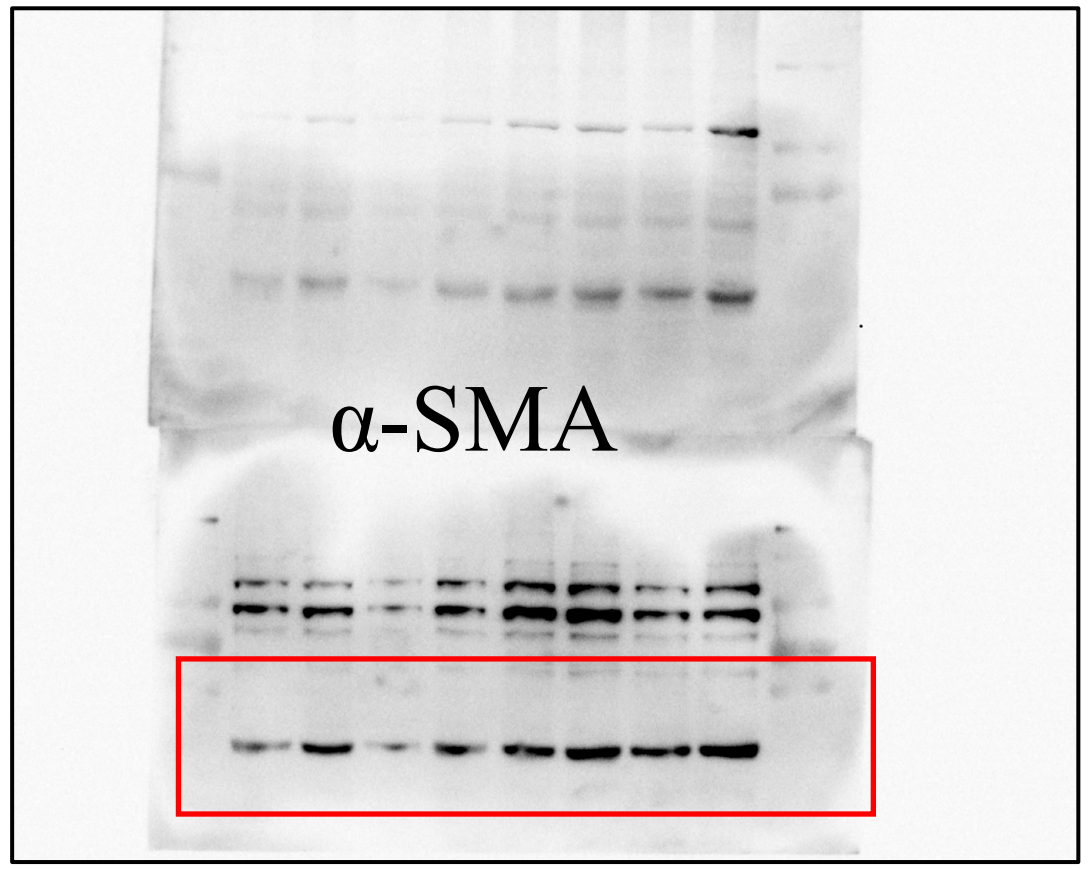
α-SMA

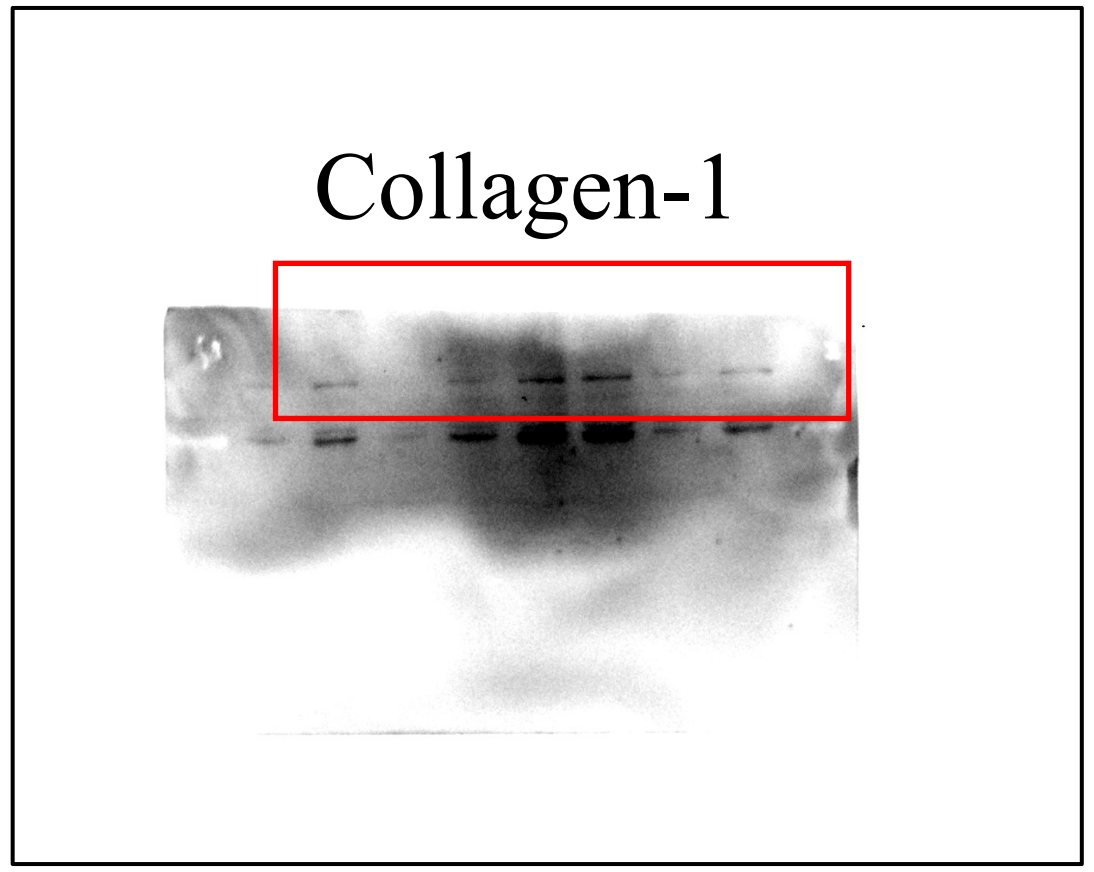
Collagen-1

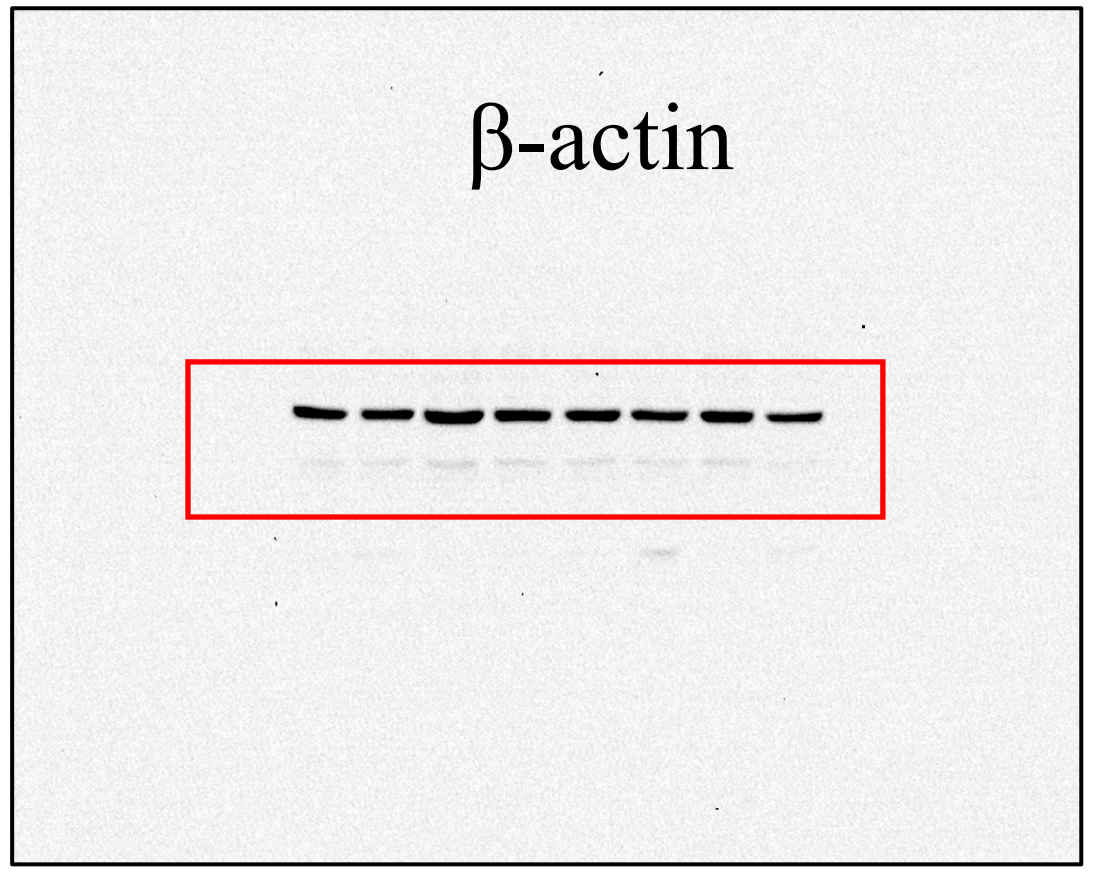
β-actin

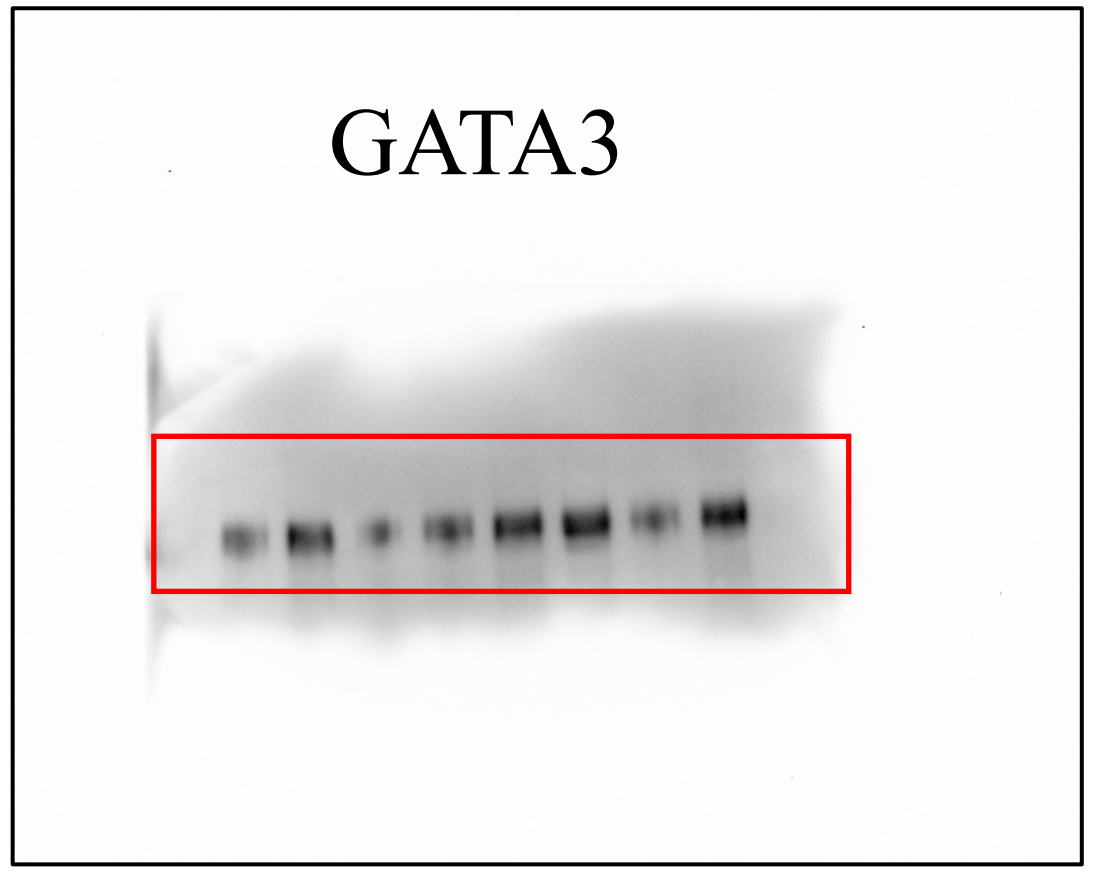
GATA3

E

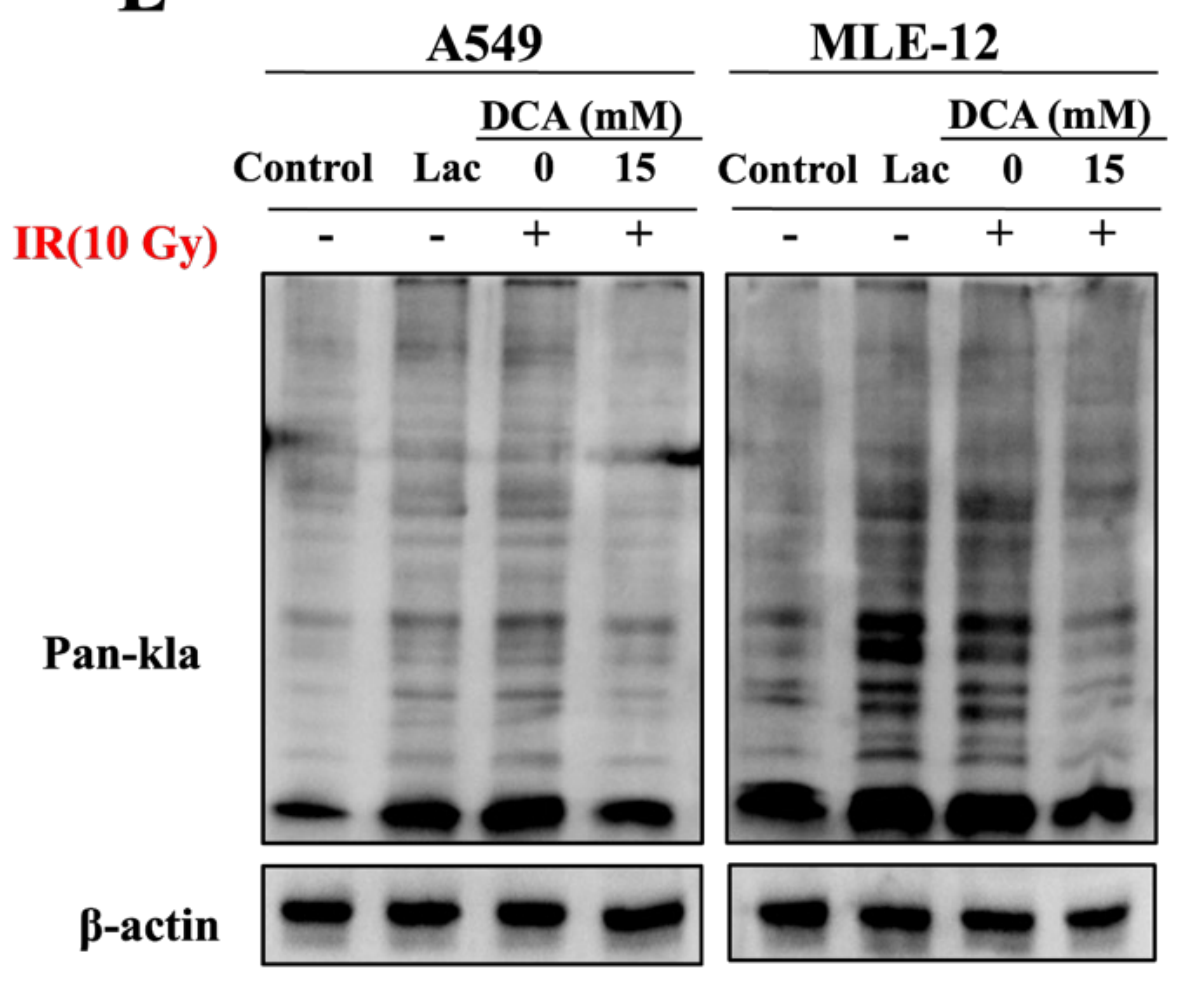

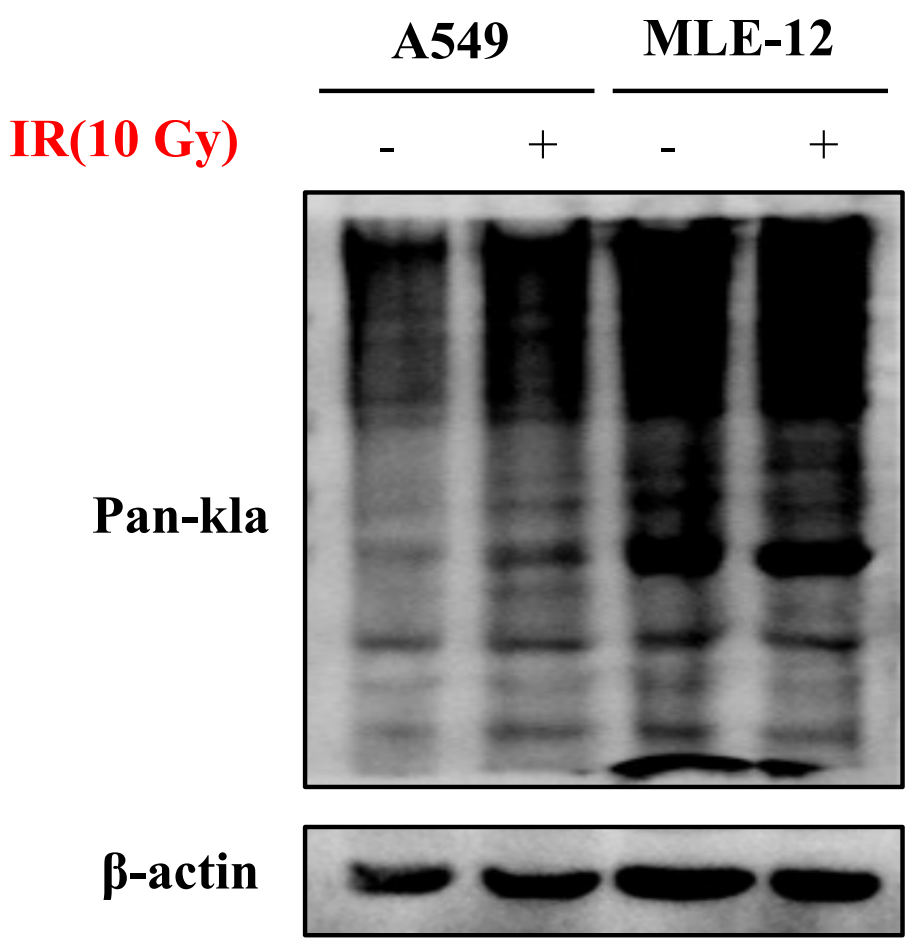

Pan-kla

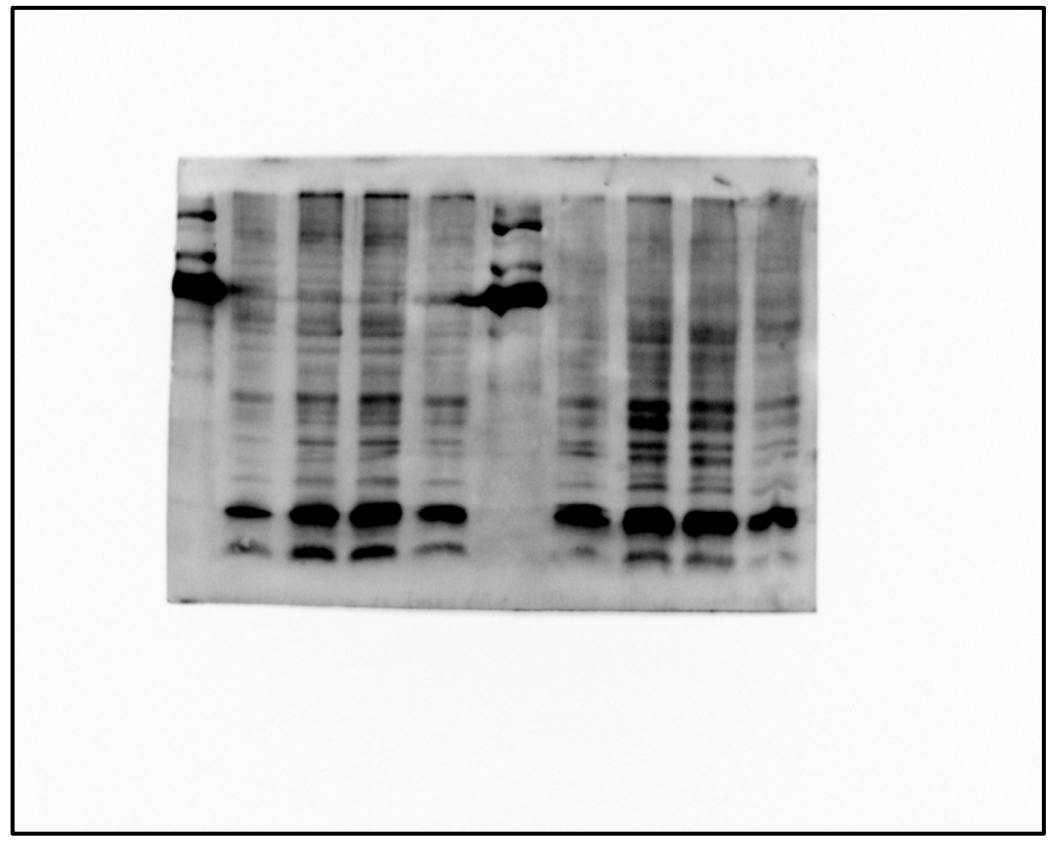

β-actin

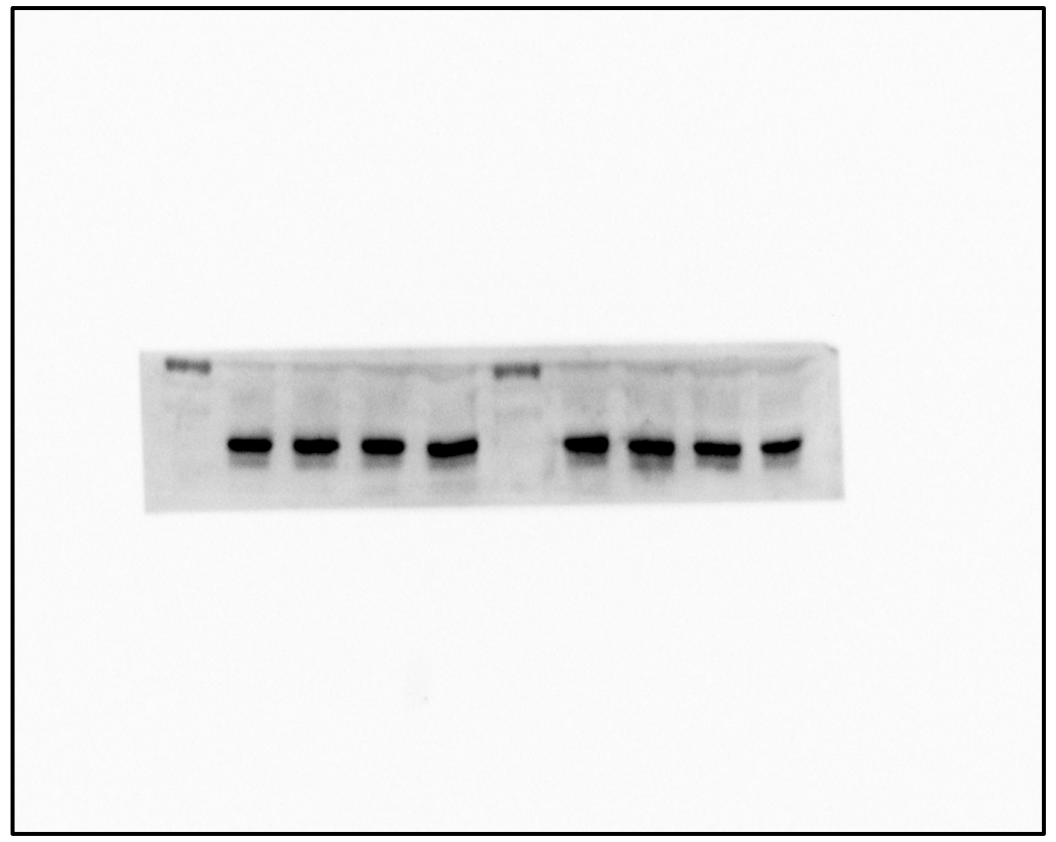

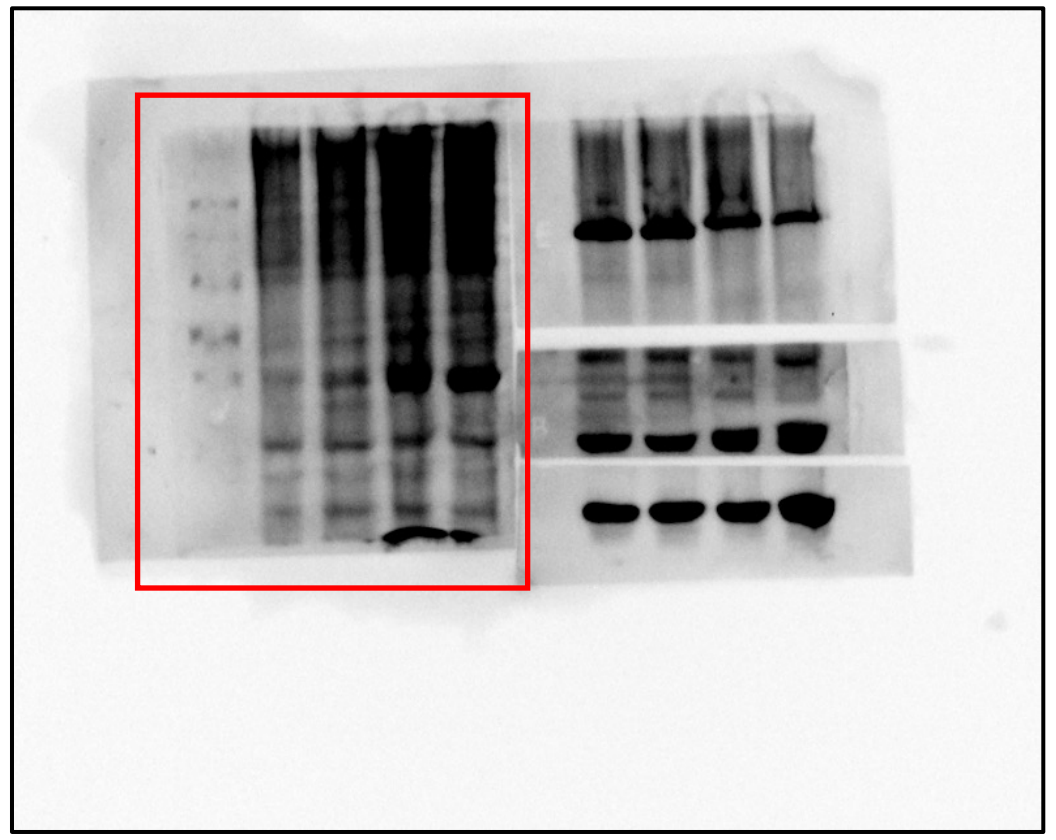

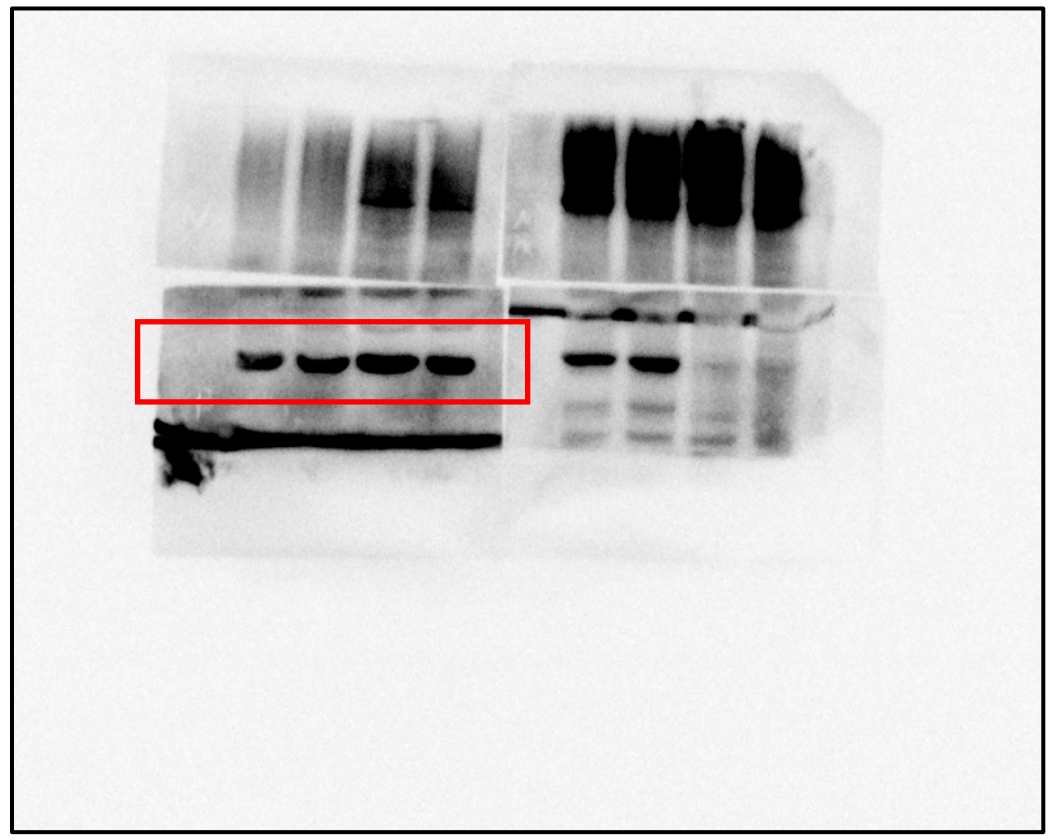

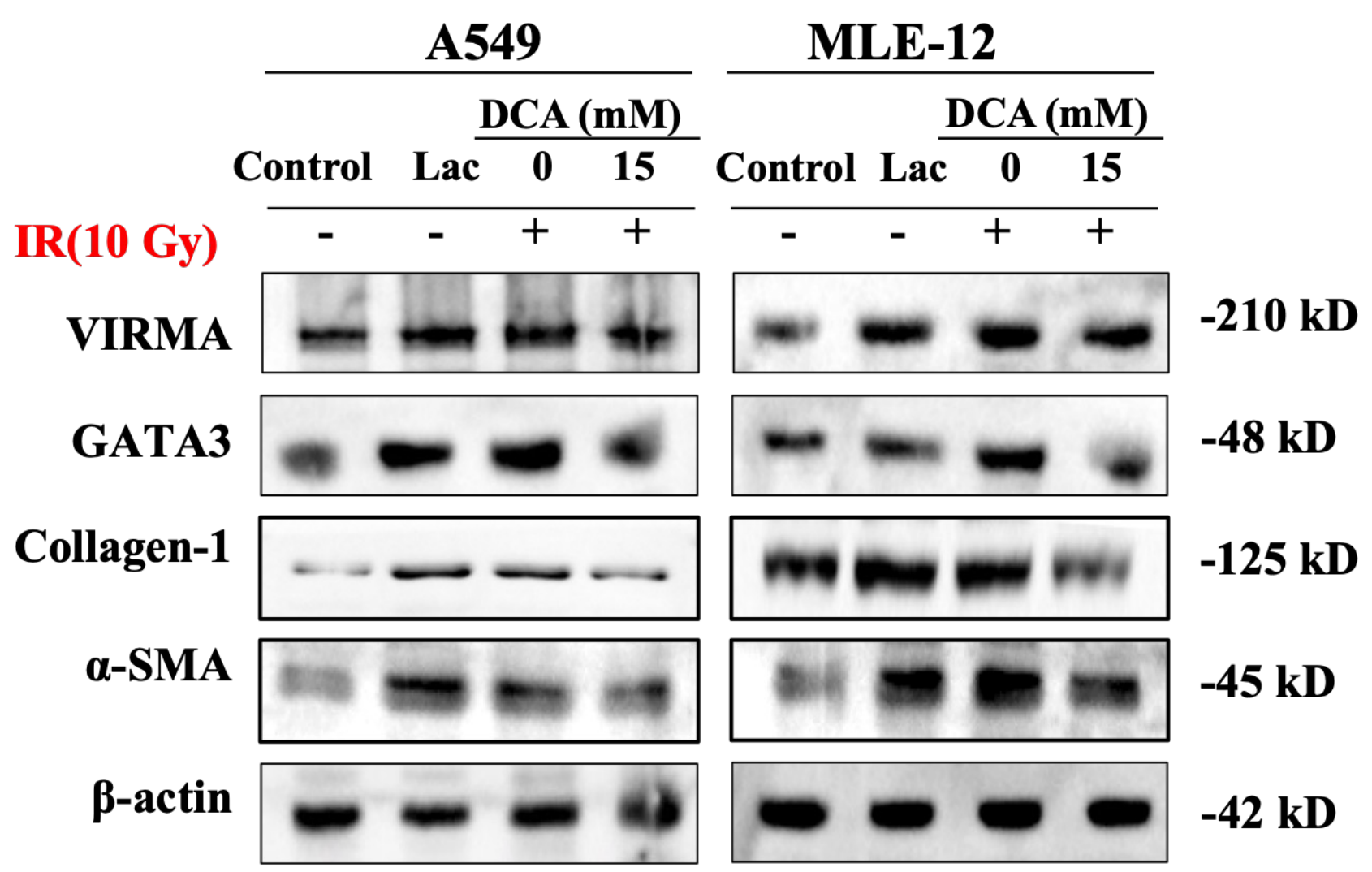

VIRMA

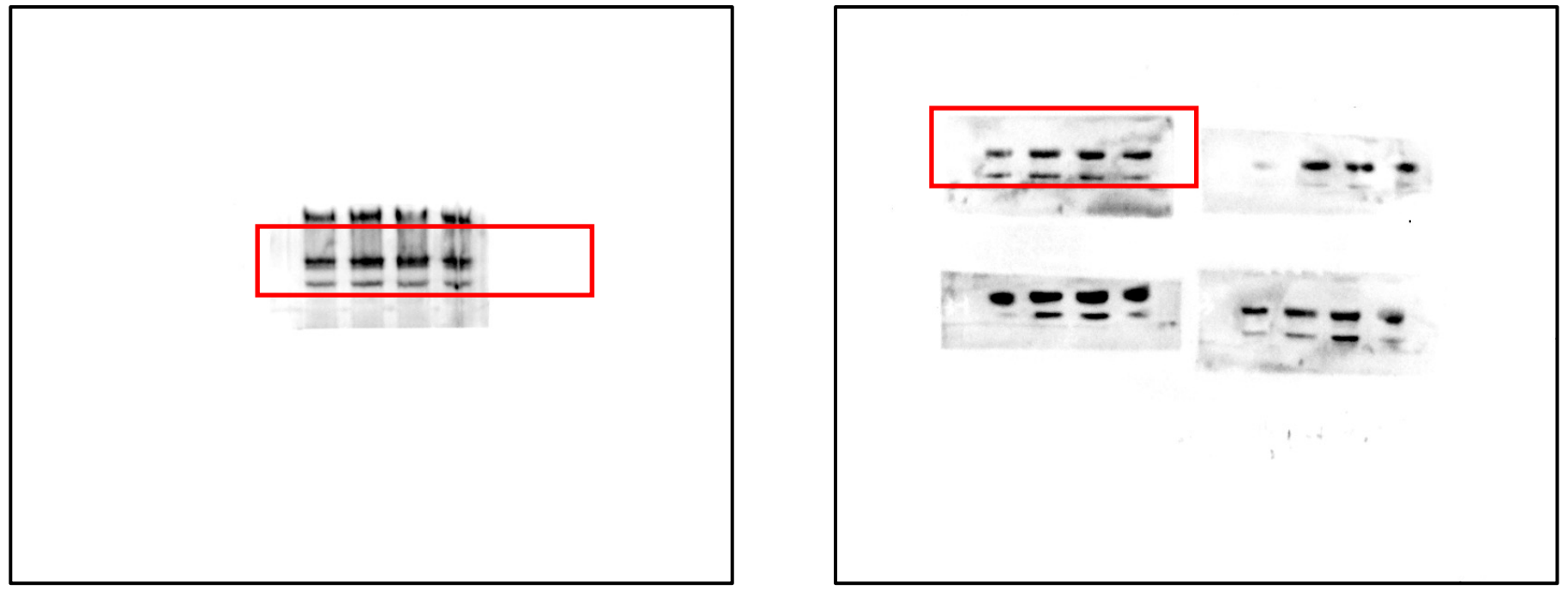

GATA3

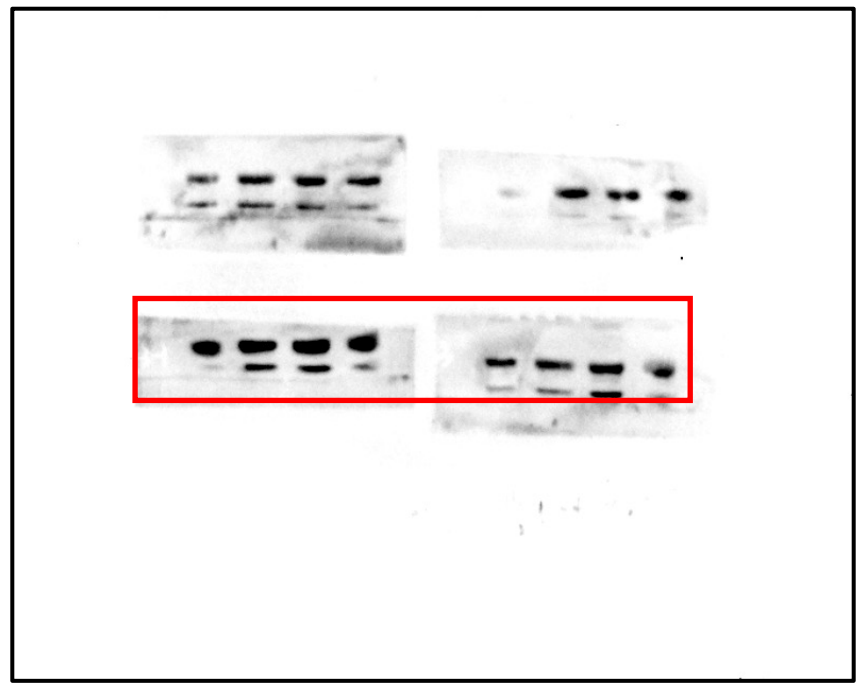

β-actin

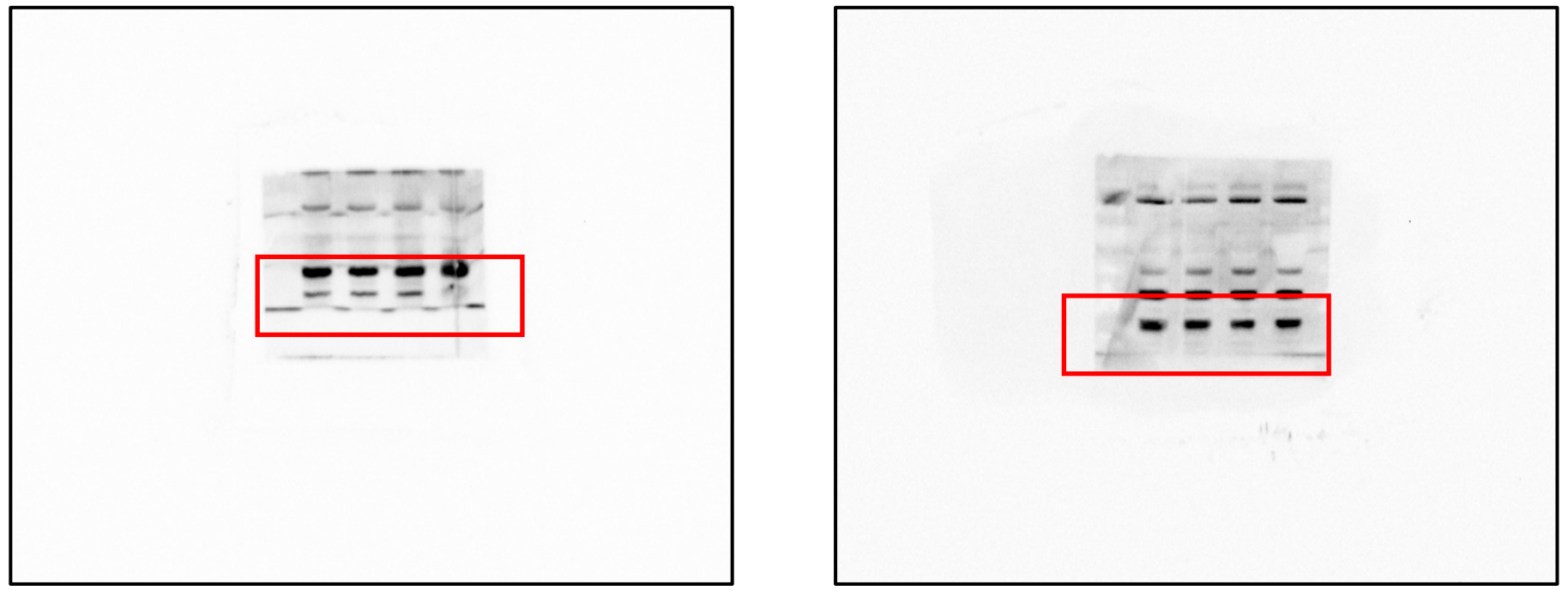

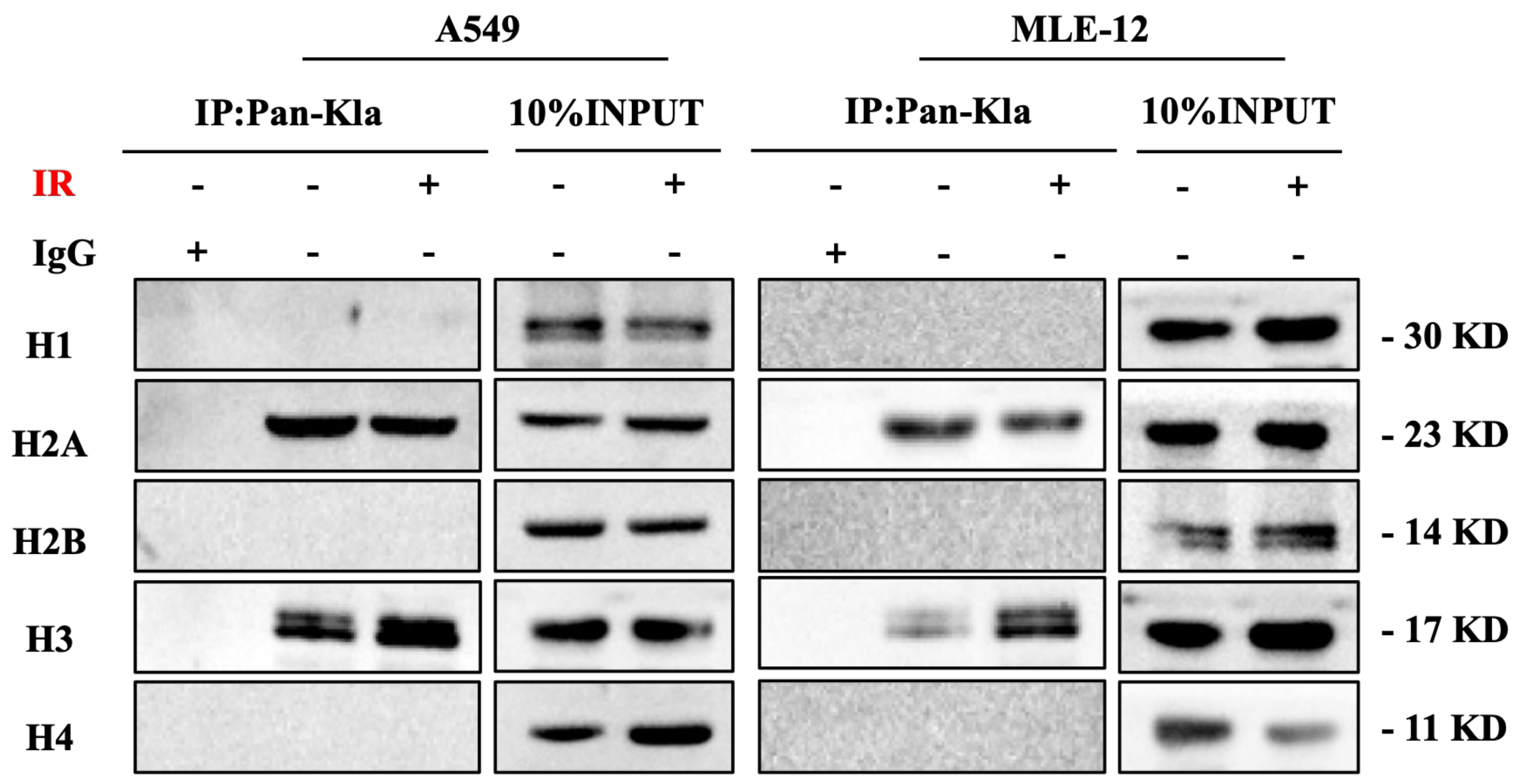
A549
MLE-12
IP:Pan-Kla
10%INPUT
IP:Pan-Kla
10%INPUT
IR
IgG
H1
H2A
H2B
H3
H4
- 30 KD
- 23 KD
- 14 KD
- 17 KD
- 11 KD

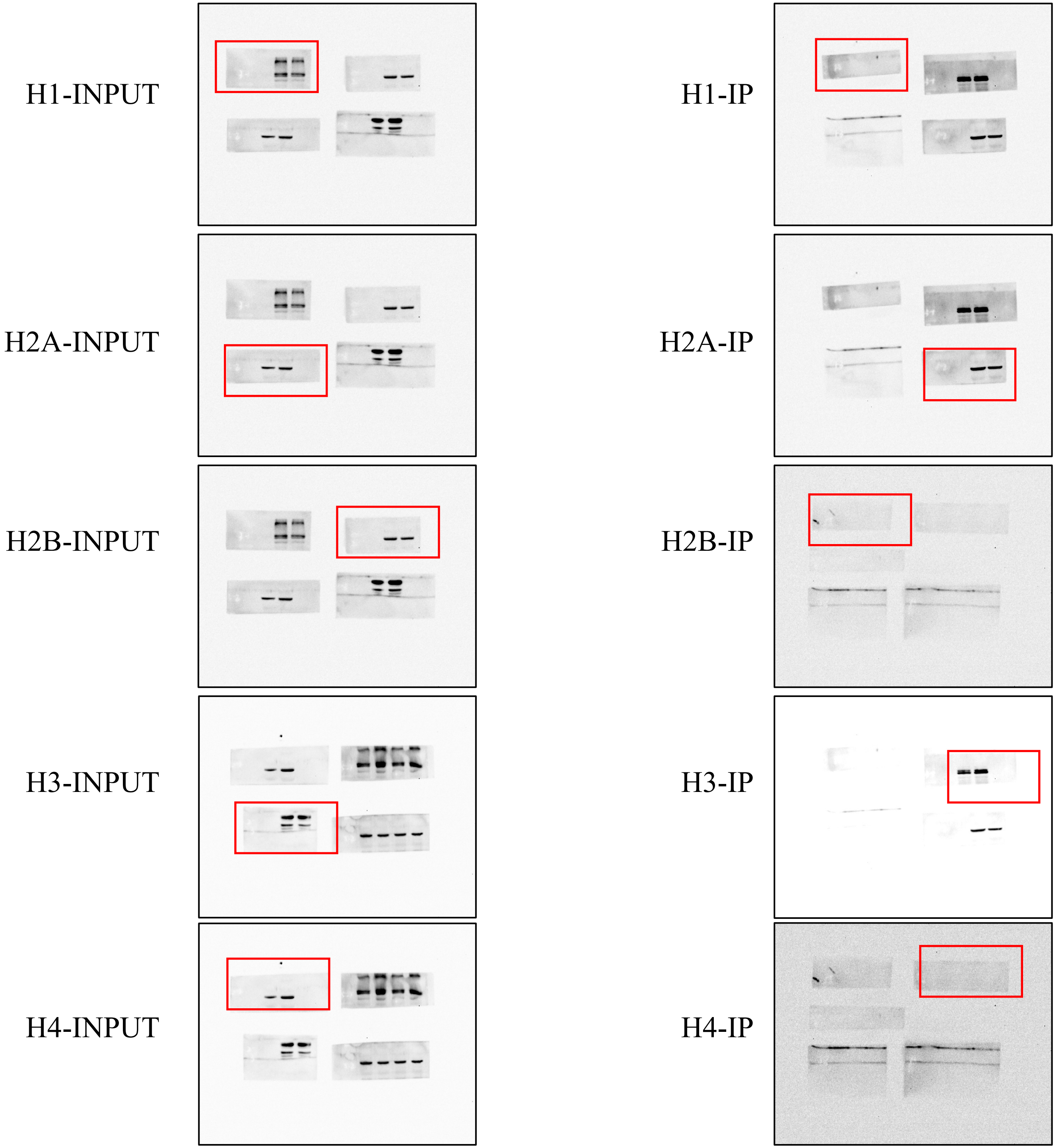
H1-INPUT
H2A-INPUT
H2B-INPUT
H3-INPUT
H4-INPUT
H1-IP
H2A-IP
H2B-IP
H3-IP
H4-IP

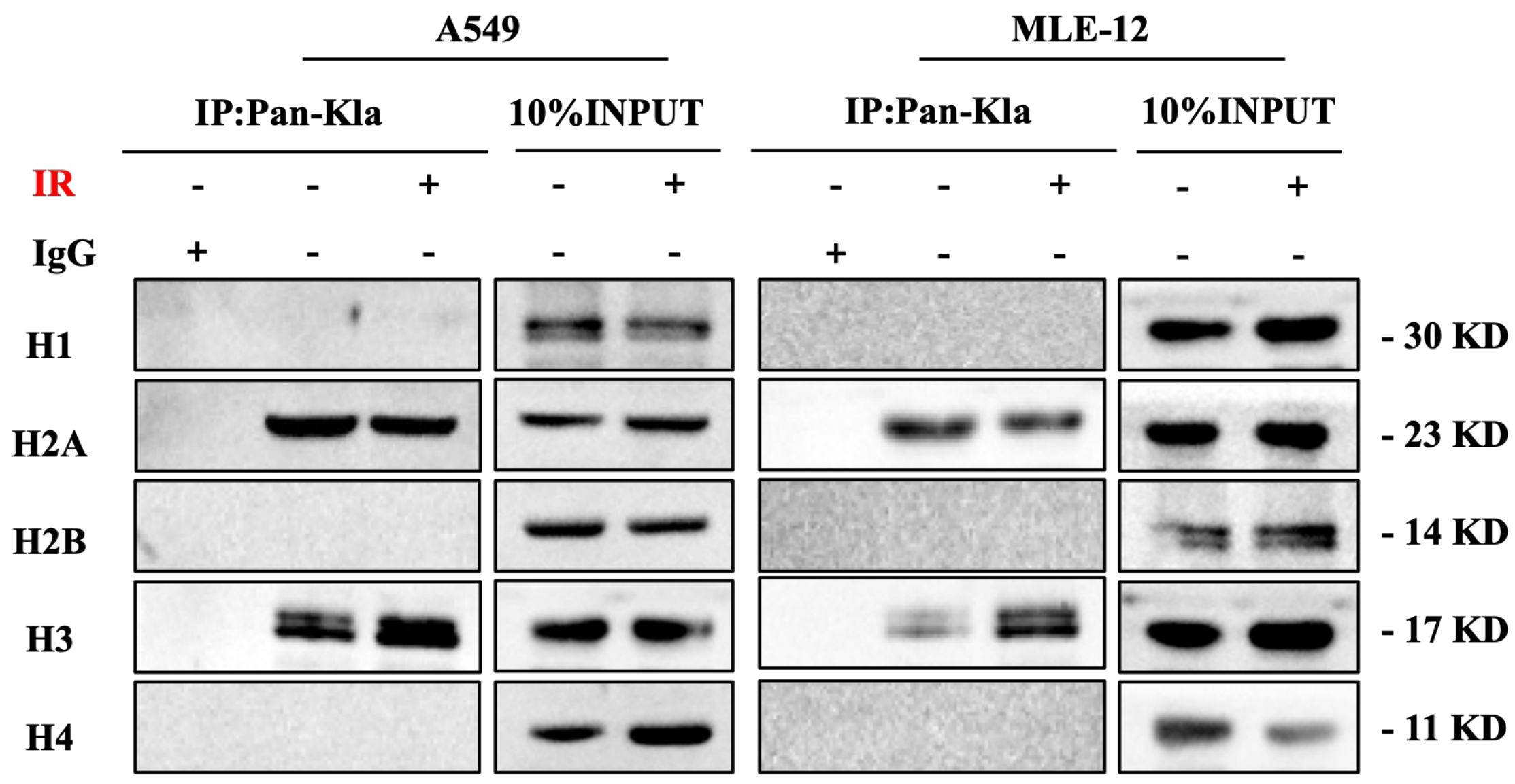
A549
MLE-12
IP:Pan-Kla
10%INPUT
IP:Pan-Kla
10%INPUT
IR
IgG
H1
H2A
H2B
H3
H4
- 30 KD
- 23 KD
- 14 KD
- 17 KD
- 11 KD

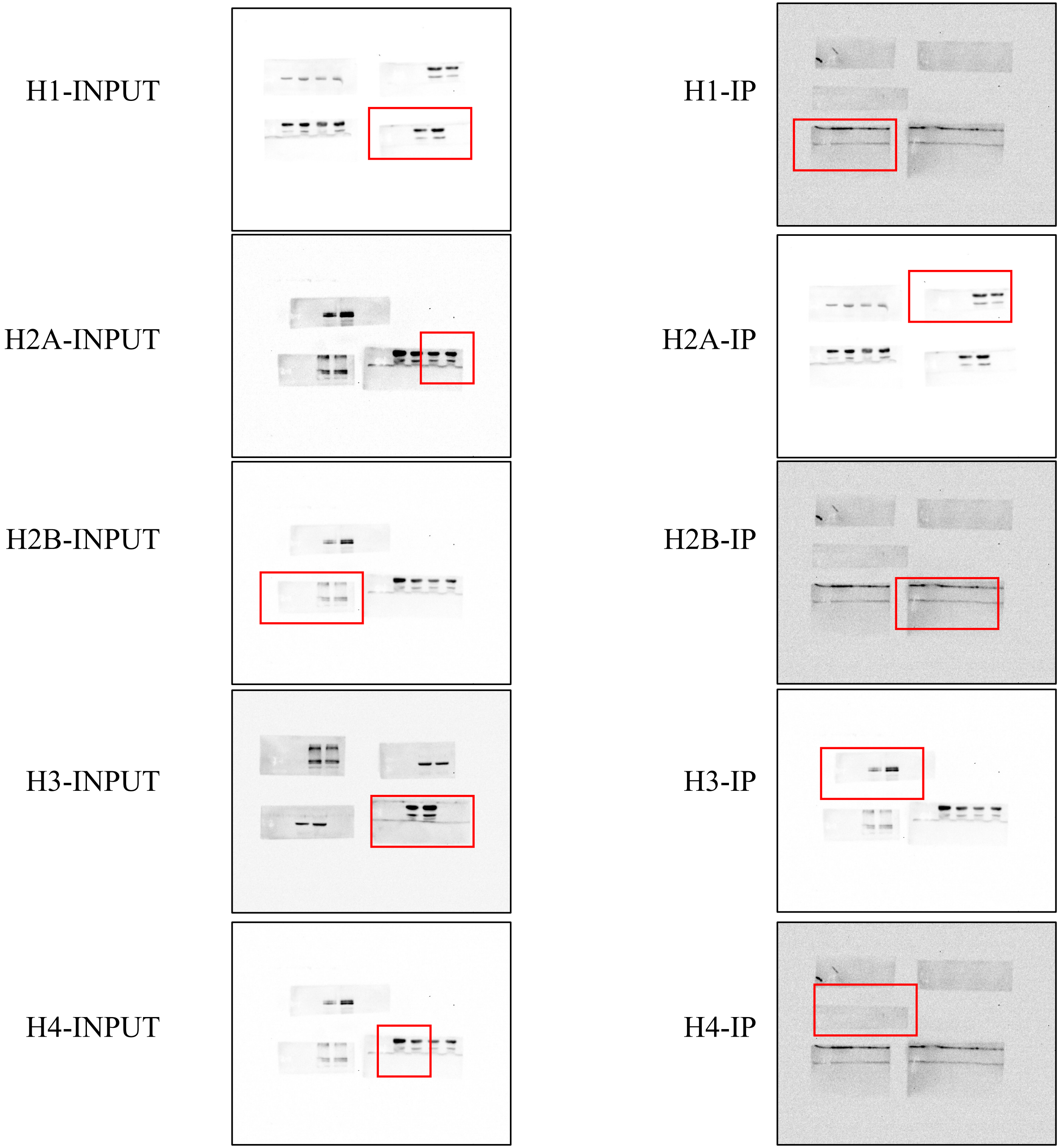
H1-INPUT
H1-IP
H2A-INPUT
H2A-IP
H2B-INPUT
H2B-IP
H3-INPUT
H3-IP
H4-INPUT
H4-IP

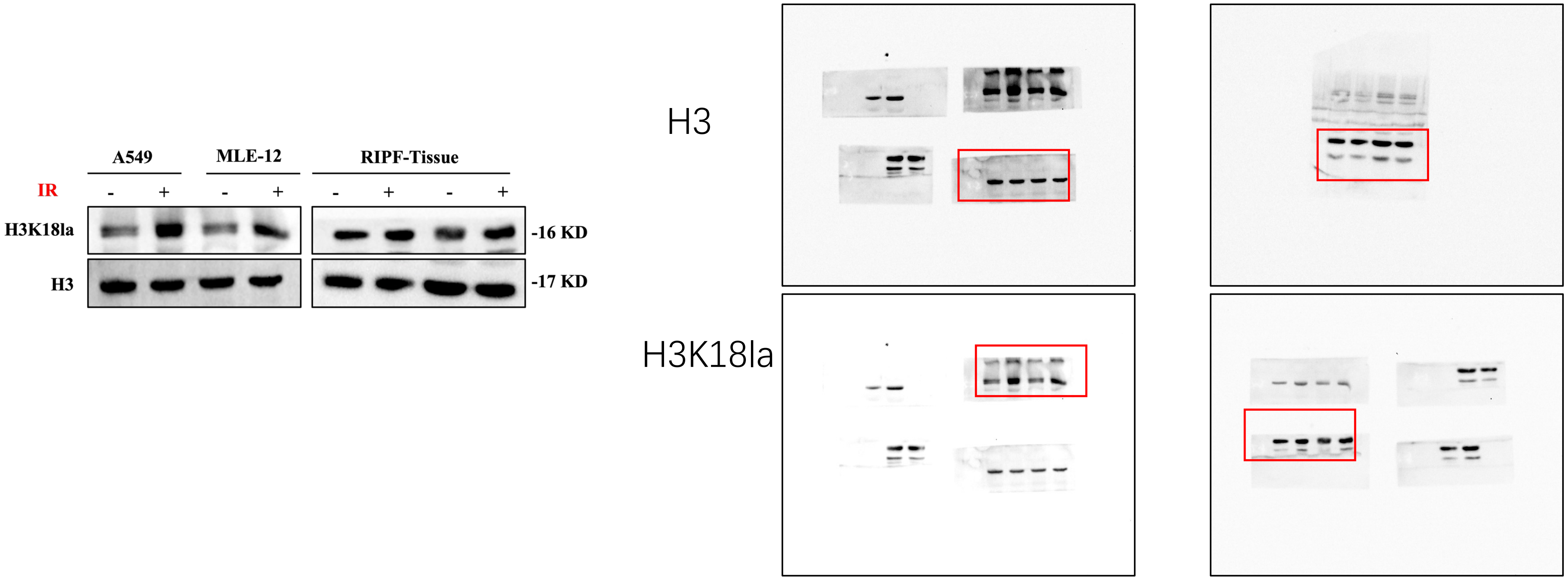
A549
MLE-12
RIPF-Tissue
IR
- + - + - + - +
H3K18la
H3
-16 KD
-17 KD
H3
H3K18la

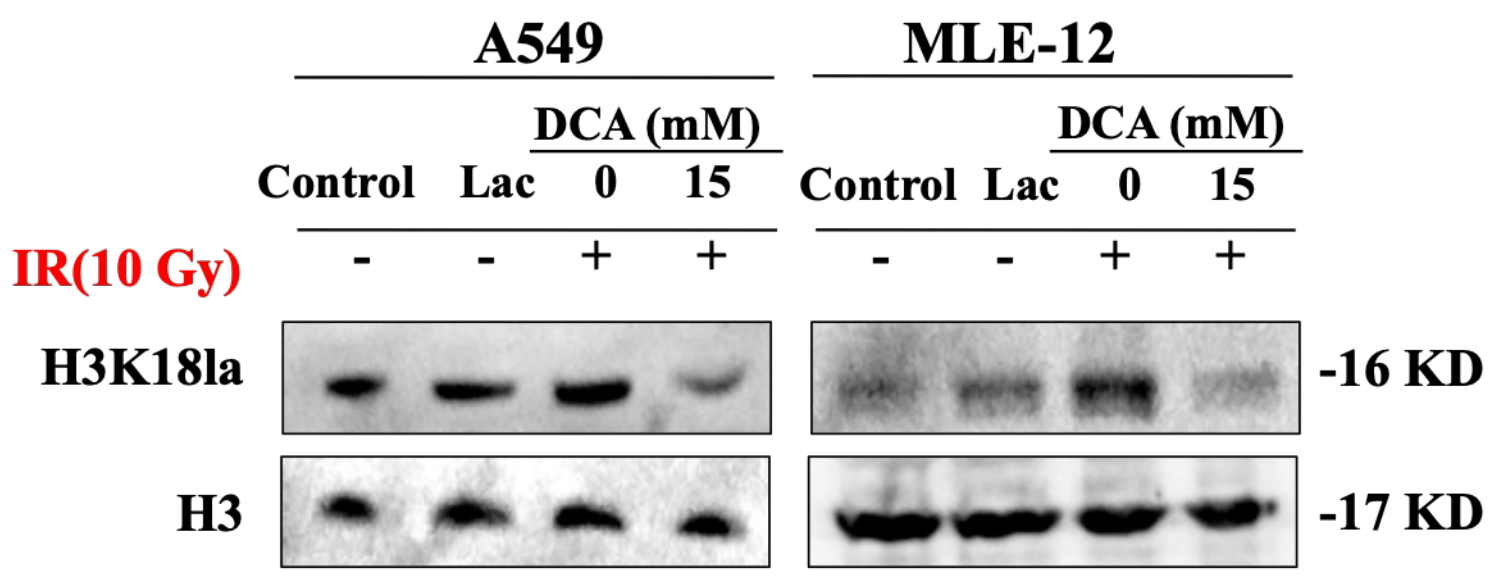
A549
MLE-12
DCA (mM)
Control
Lac
0
15
IR(10 Gy)
- - + +
H3K18la
H3
-16 KD
-17 KD

H3

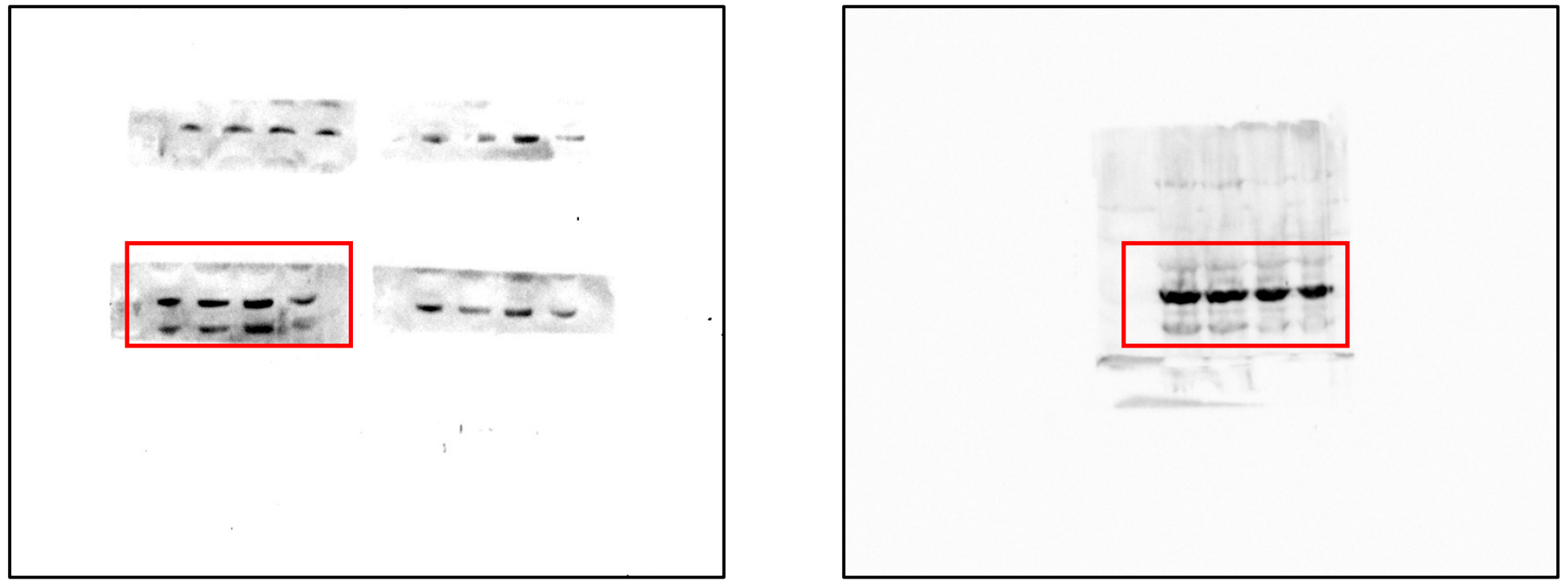

H3K18la

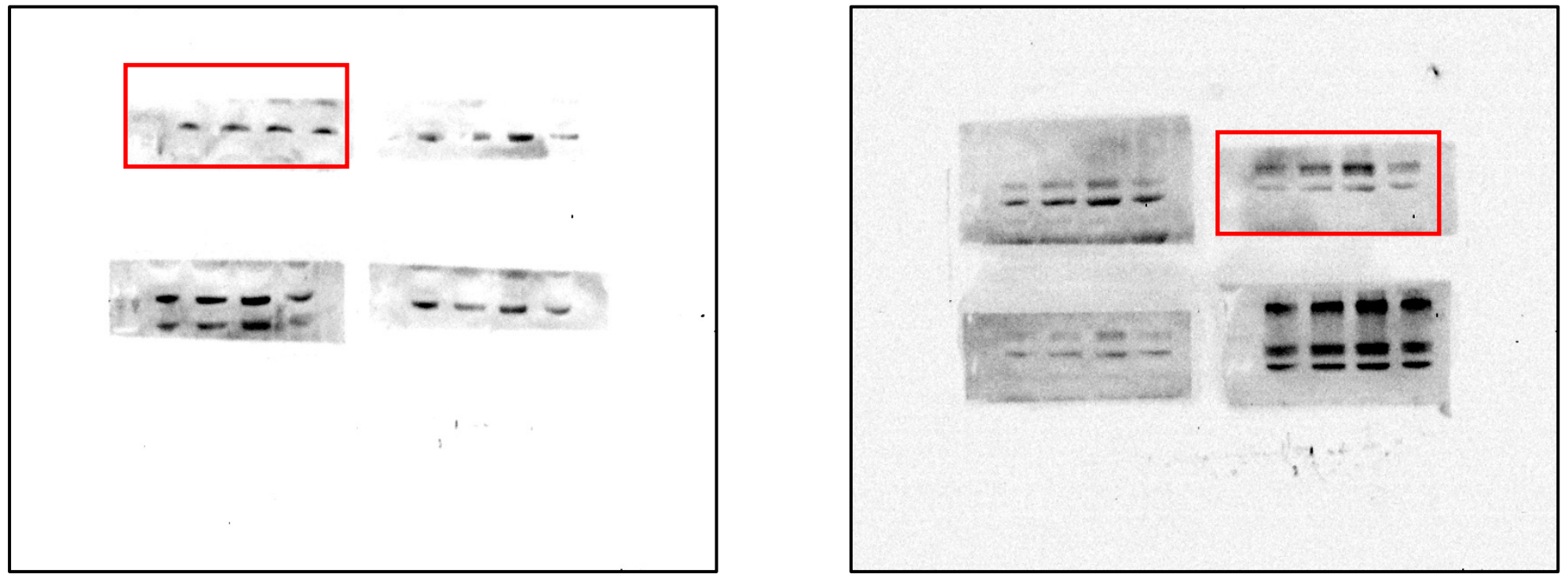

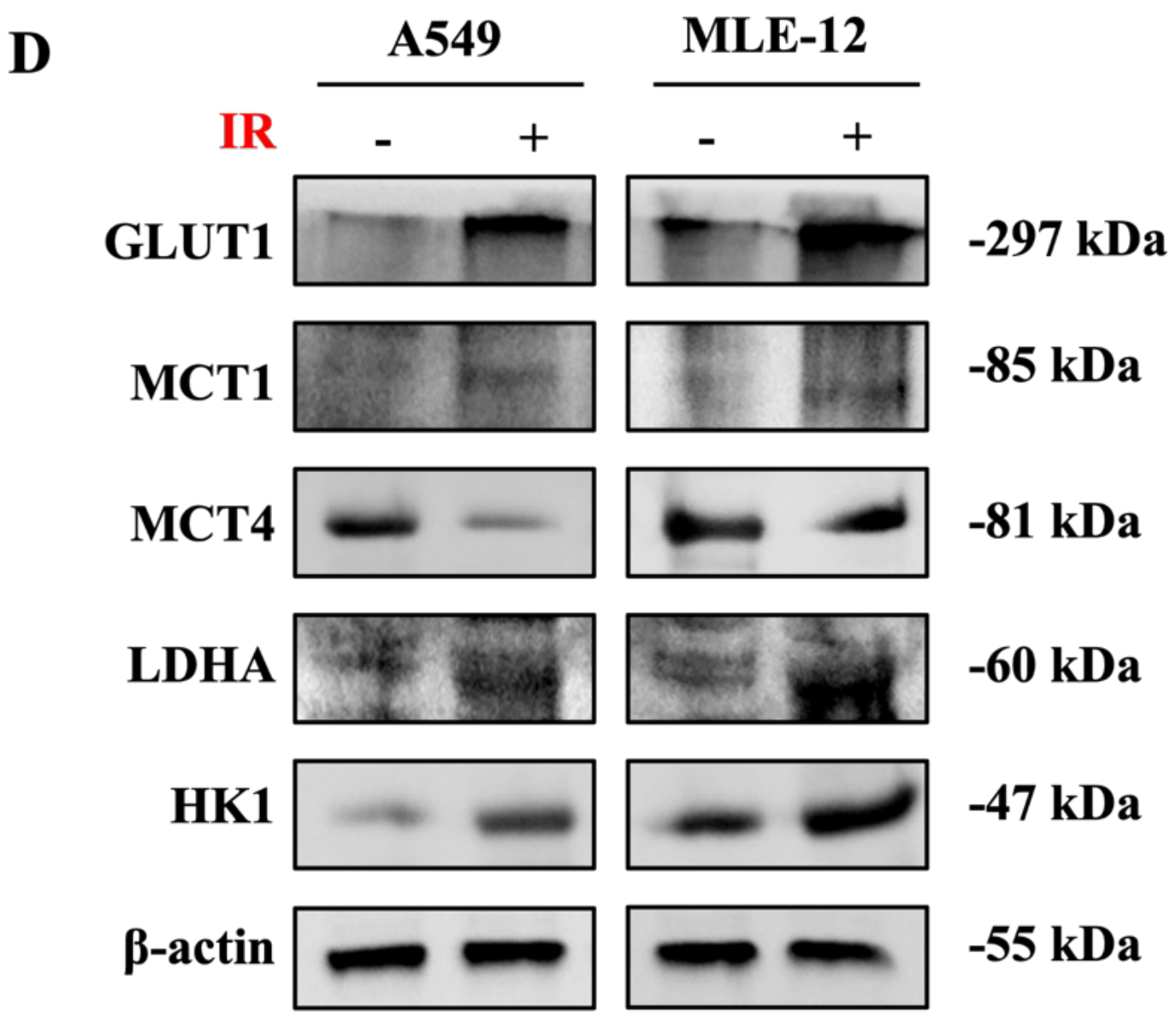

β-actin

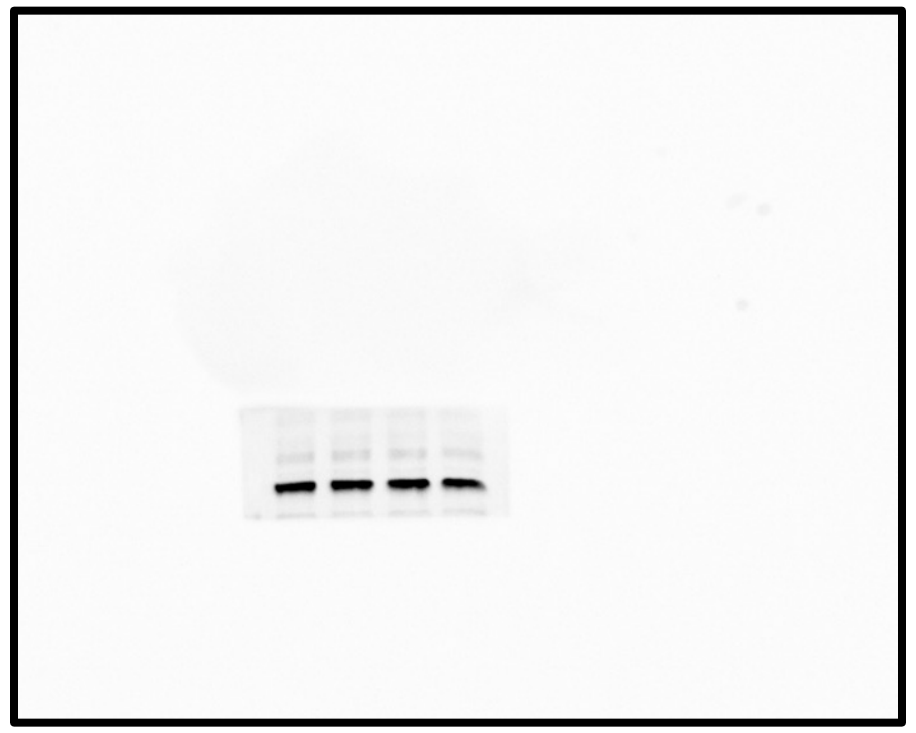

LDHA

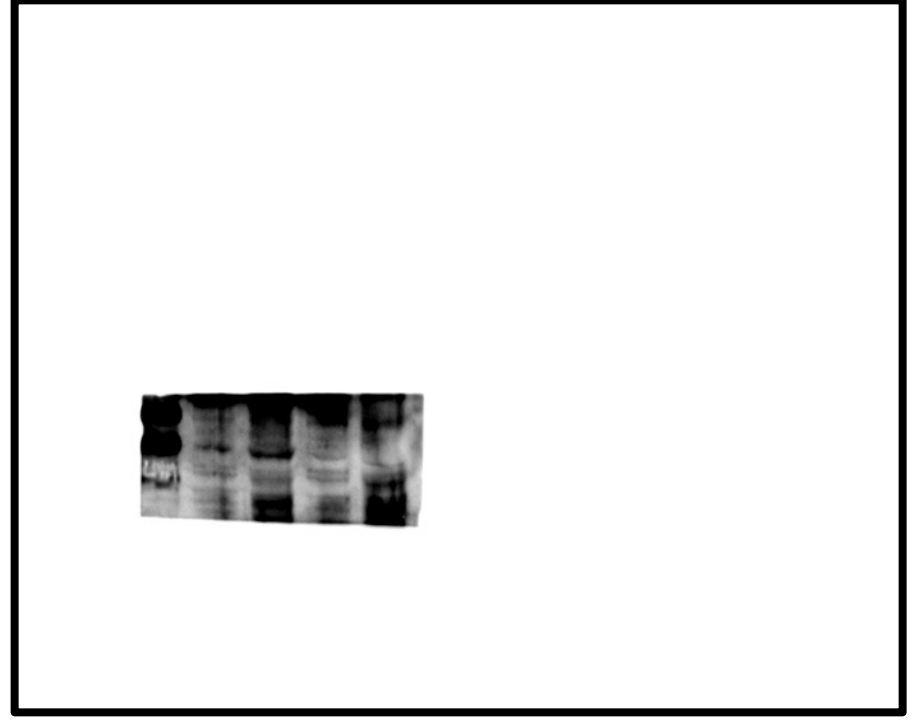

MCT1

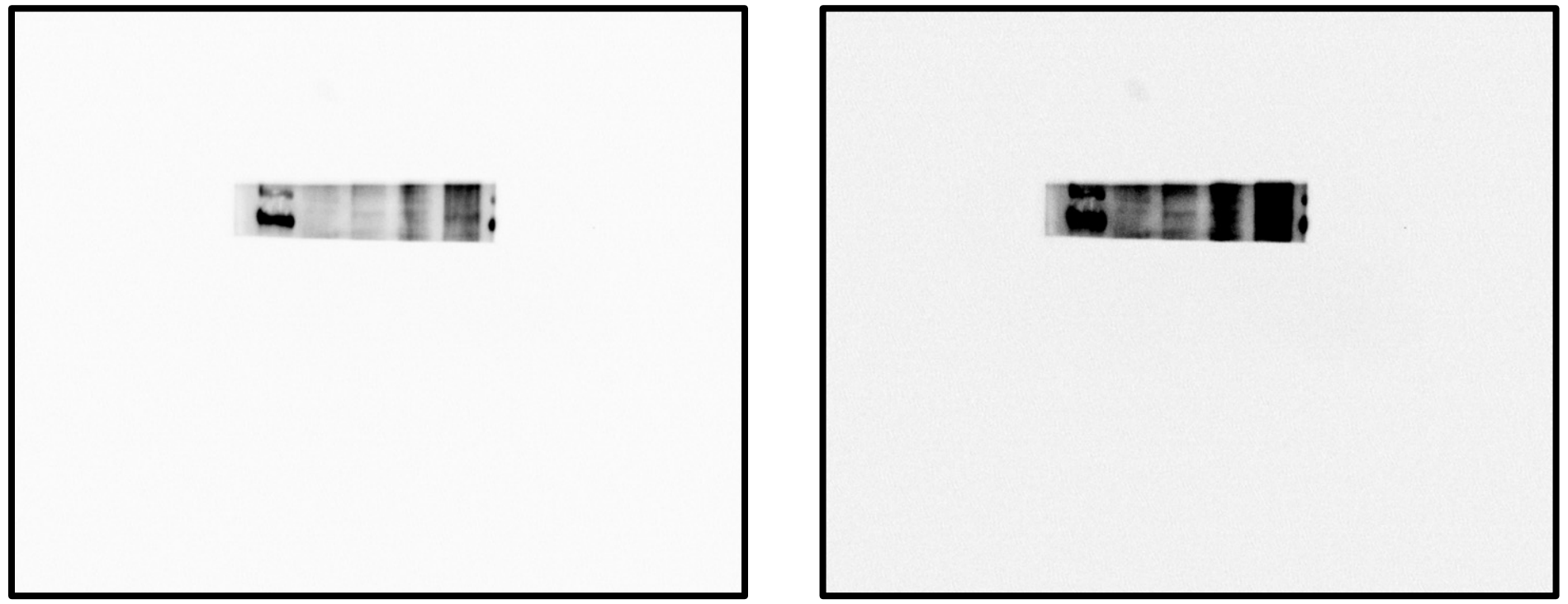

GLUT1

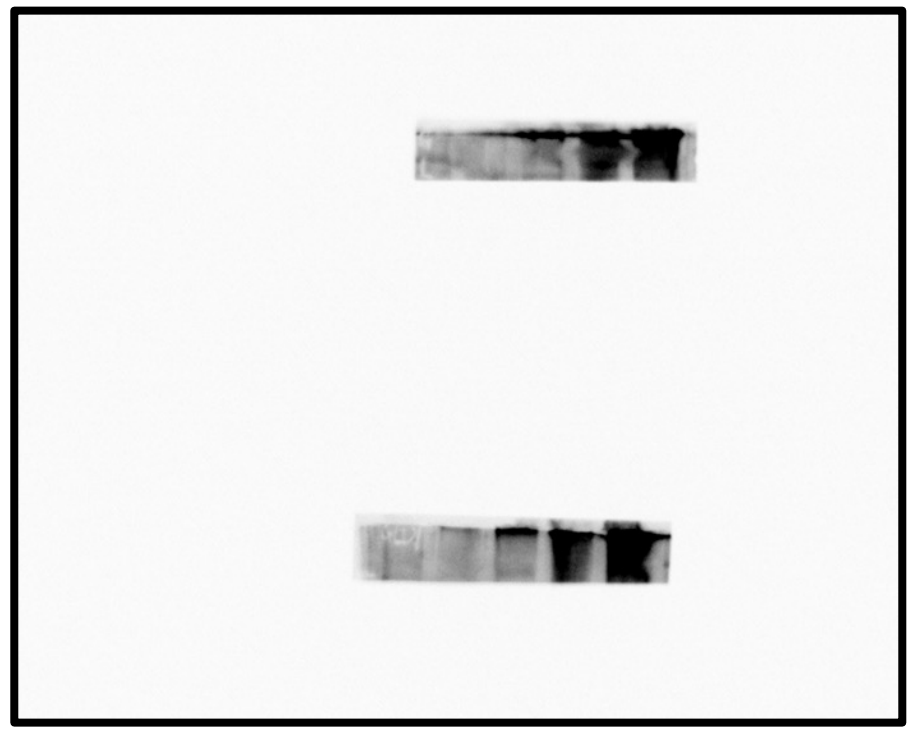

HK1

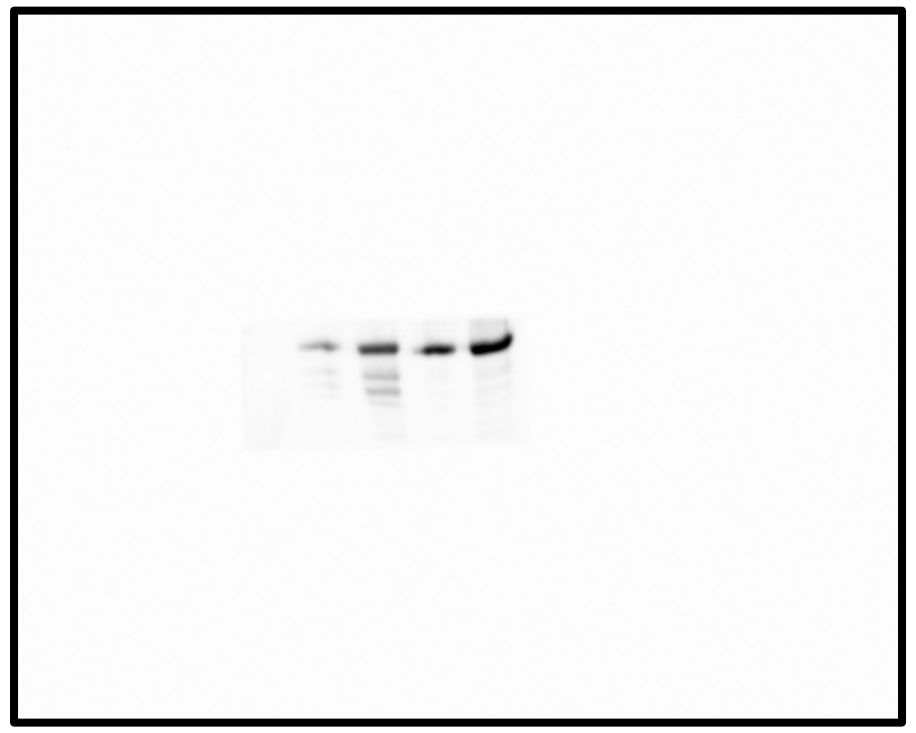

MCT4

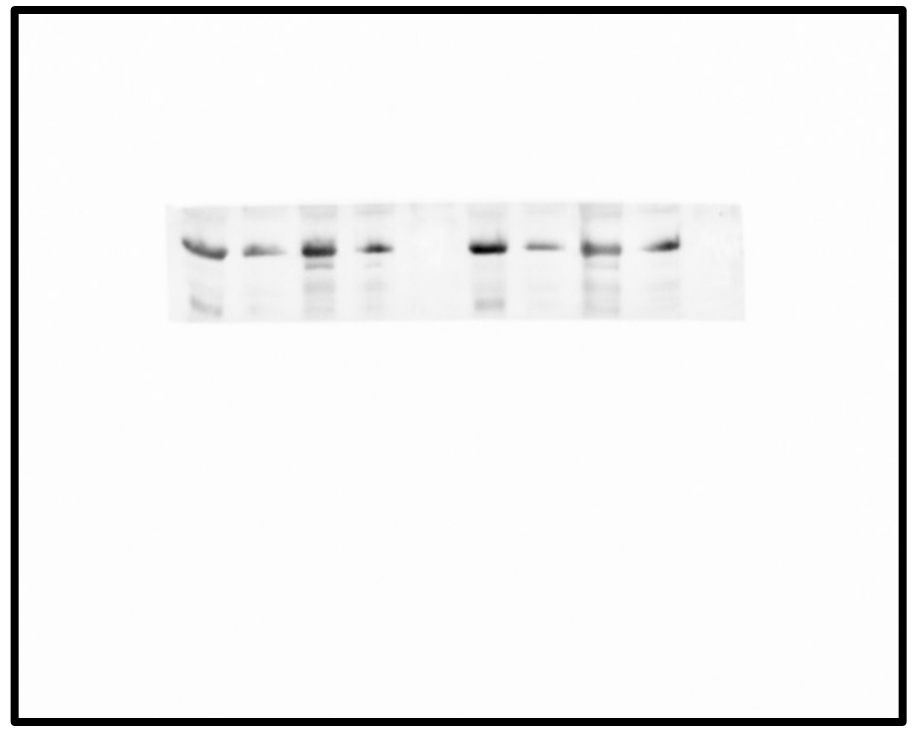

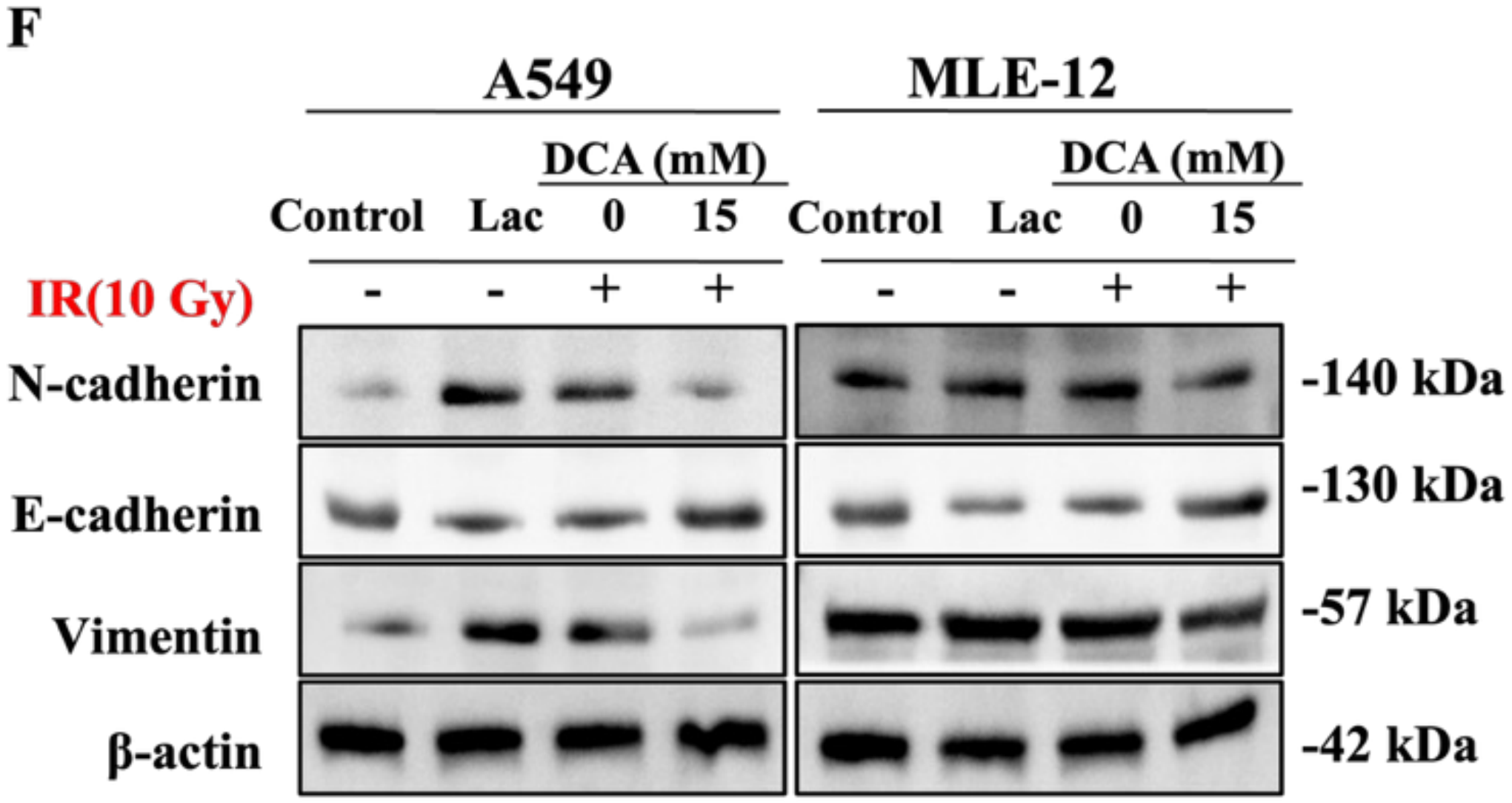

E-cadherin

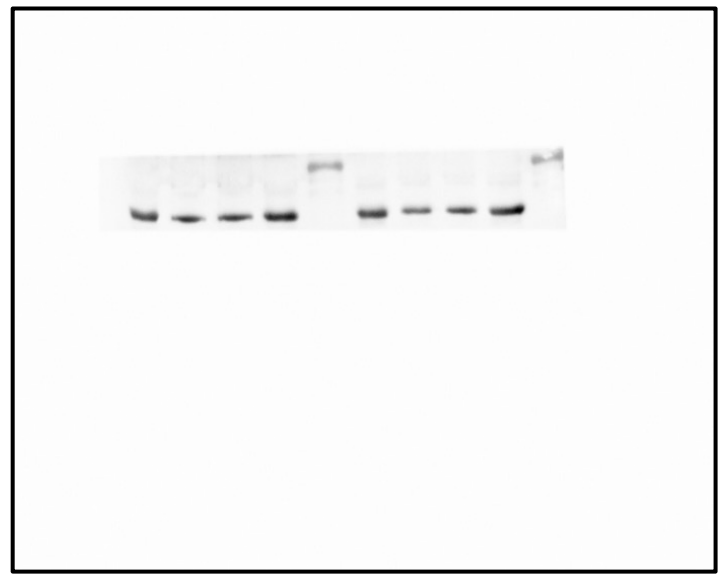

N-cadherin

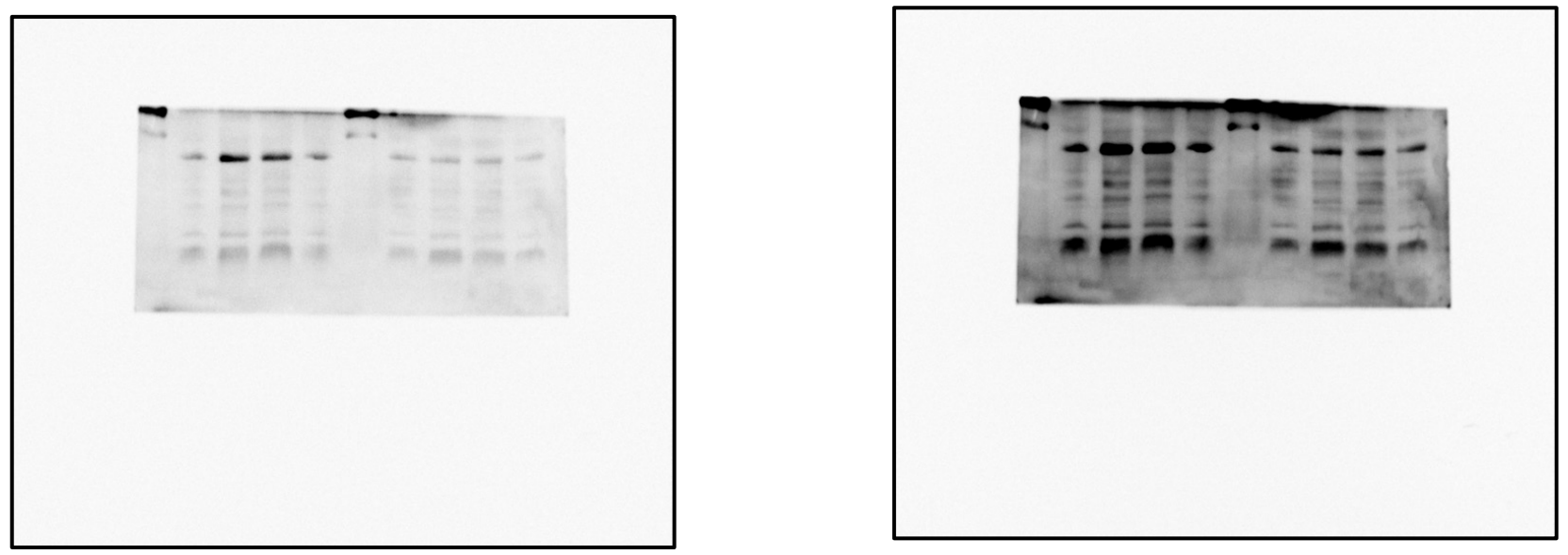

VIMENTIN

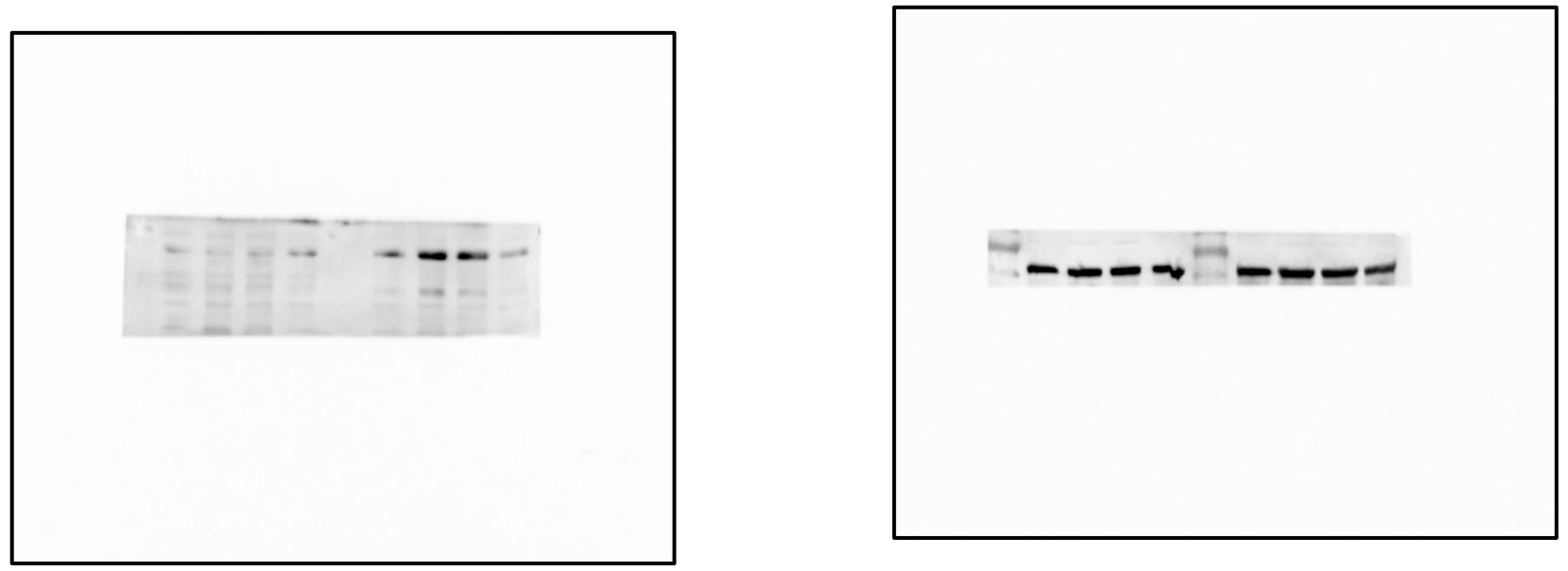

β-actin

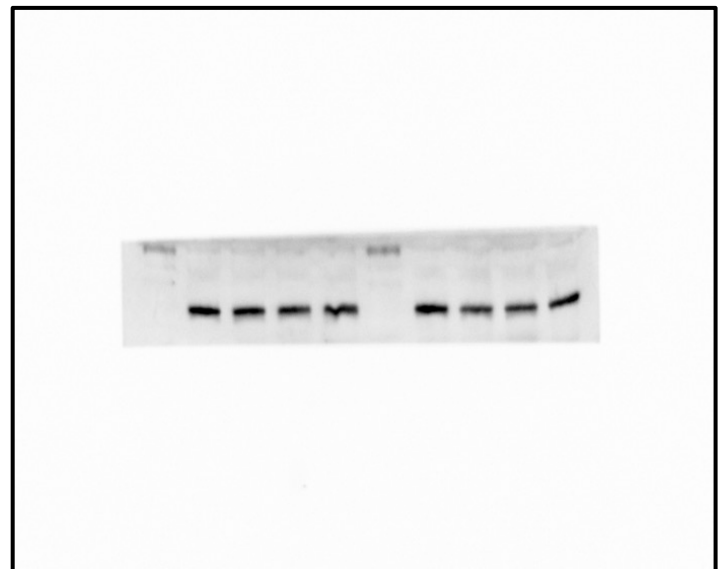

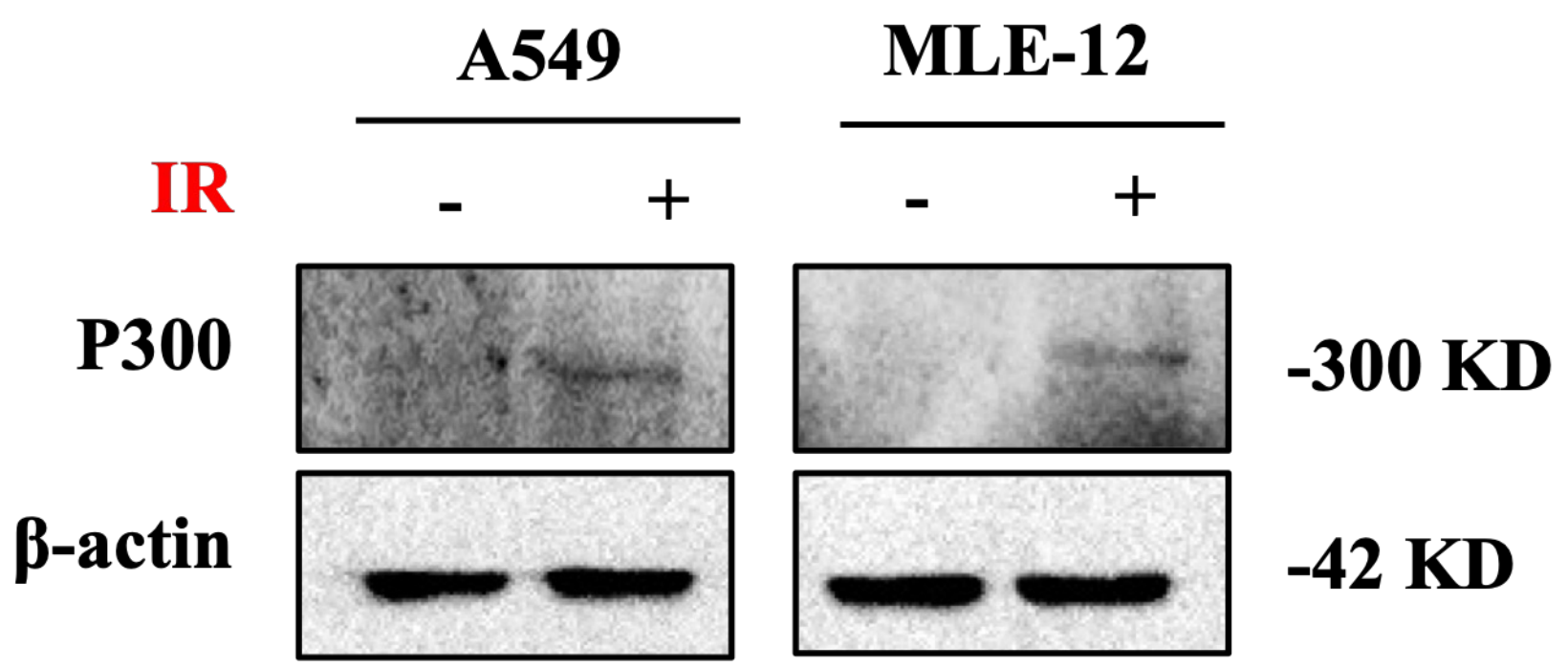

P300

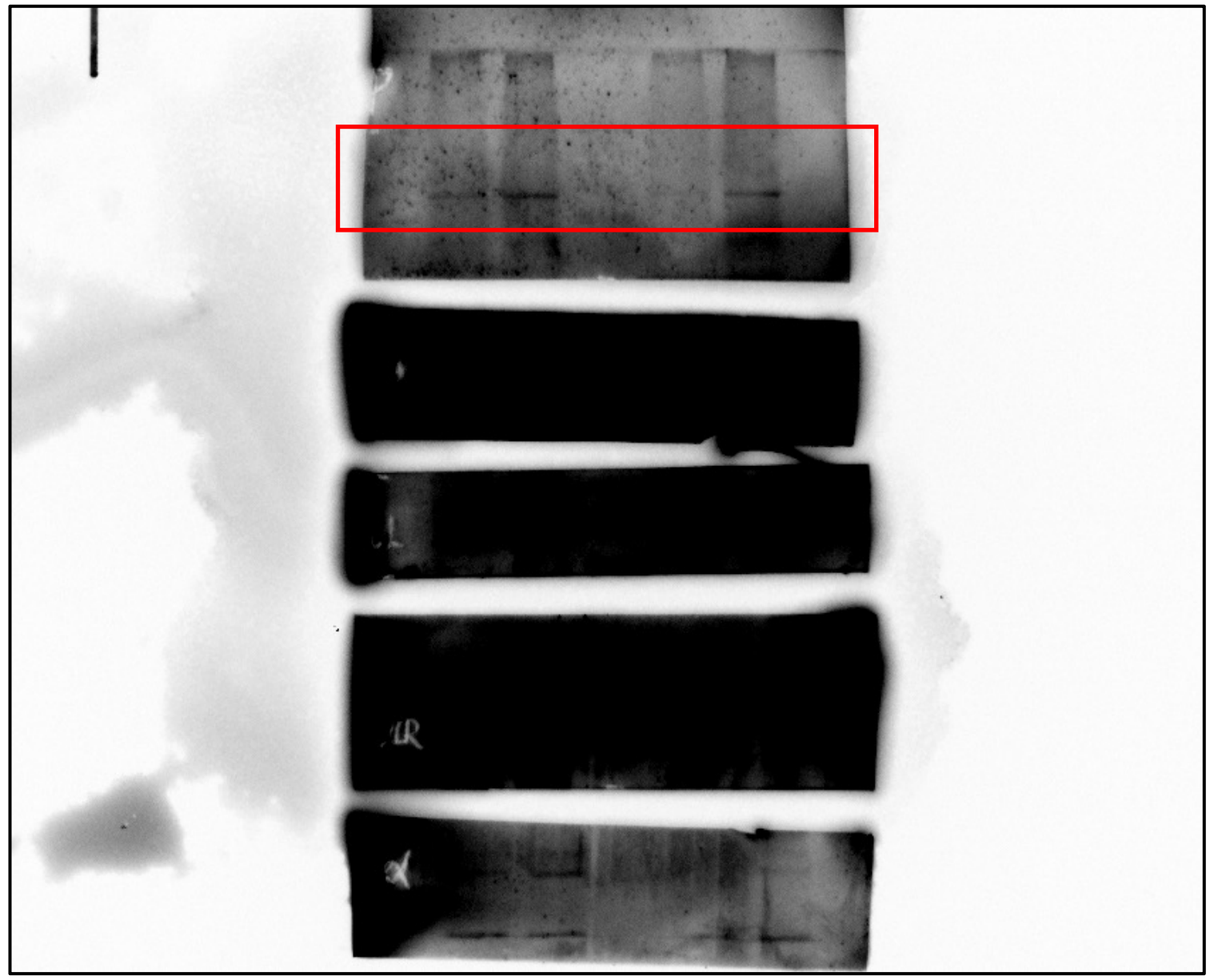

β-actin

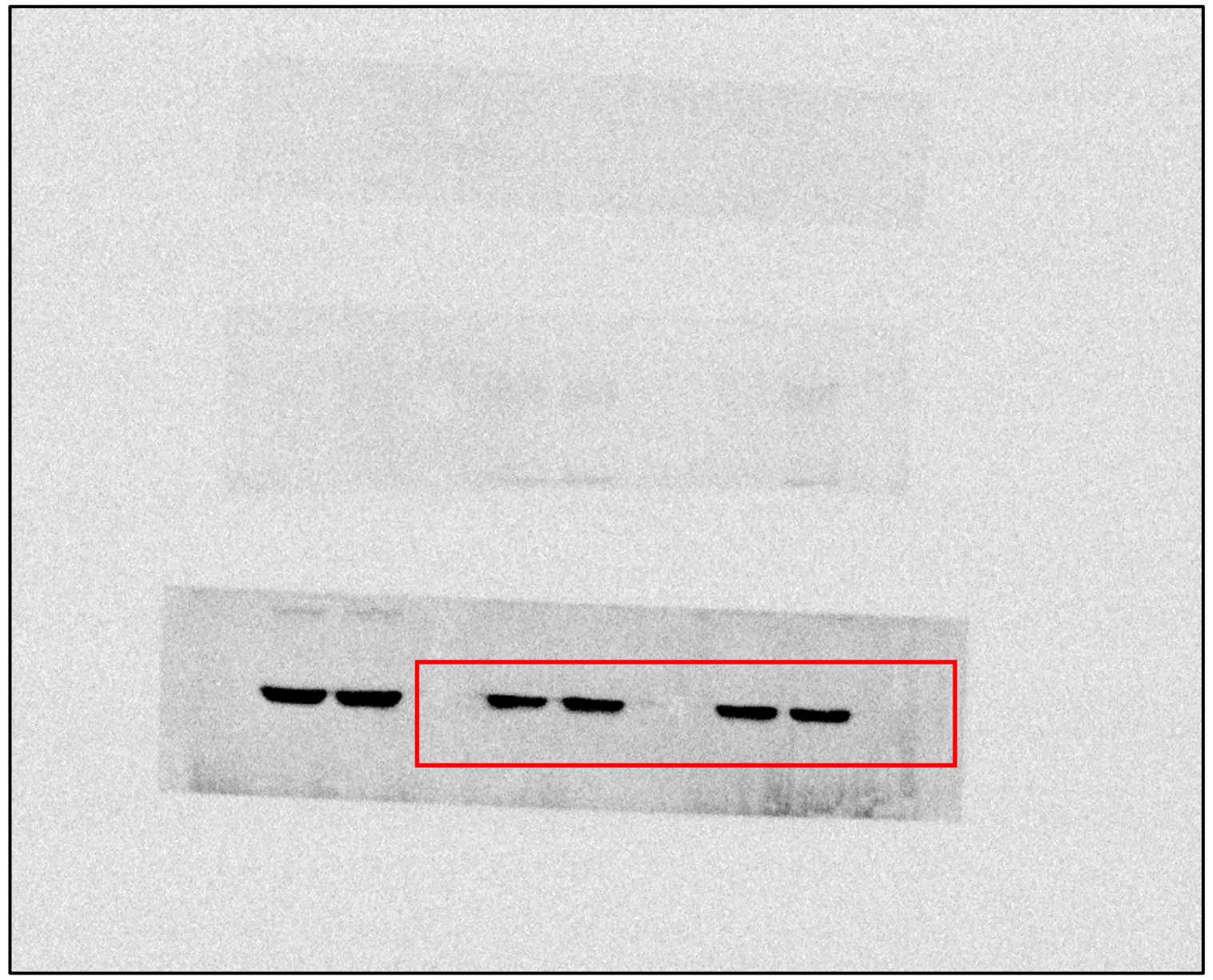

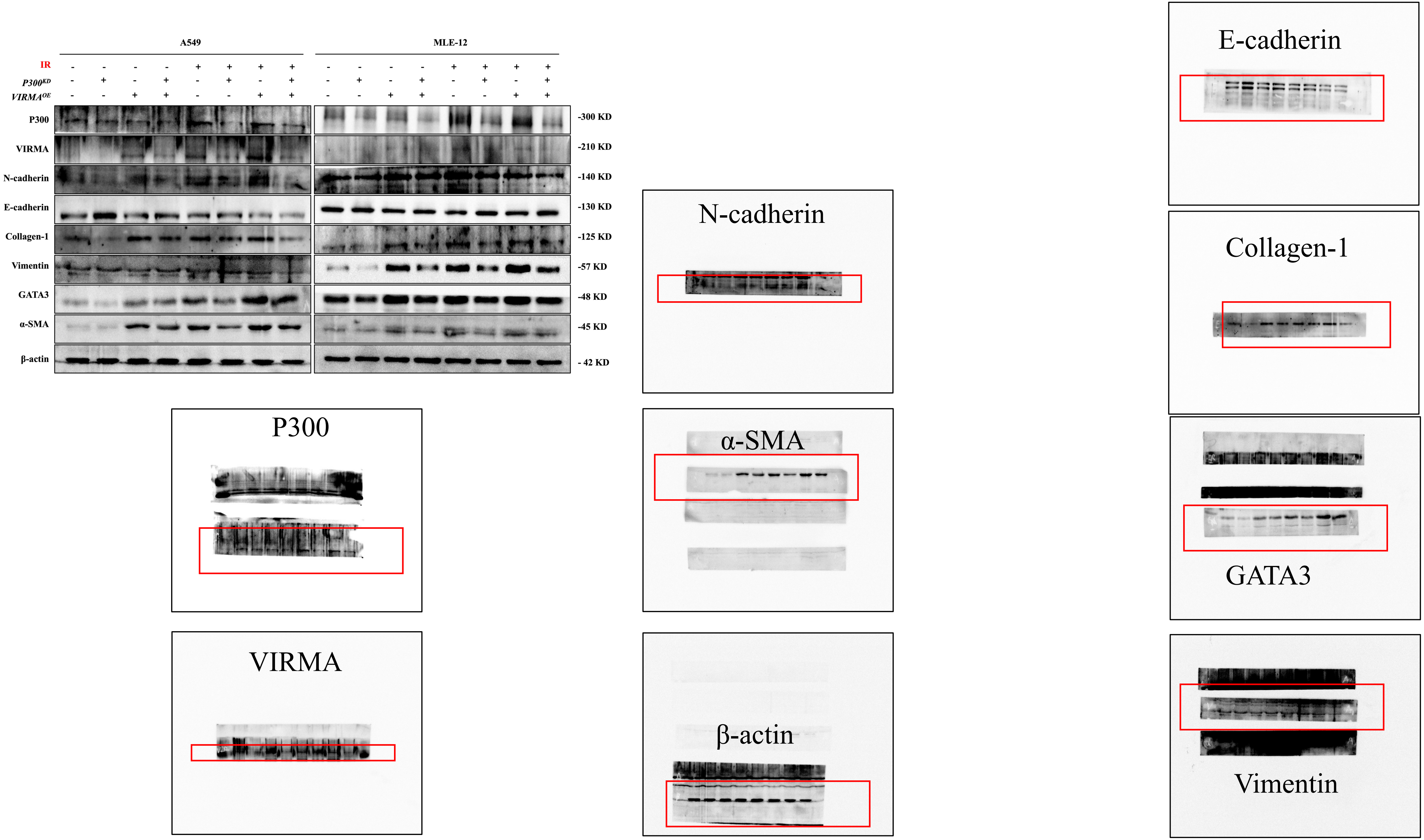

A549
MLE-12
IR
P300KD
VIRMAOE
P300
VIRMA
N-cadherin
E-cadherin
Collagen-1
Vimentin
GATA3
α-SMA
β-actin
-300 KD
-210 KD
-140 KD
-130 KD
-125 KD
-57 KD
-48 KD
-45 KD
- 42 KD
E-cadherin
N-cadherin
Collagen-1
P300
α-SMA
GATA3
VIRMA
β-actin
Vimentin

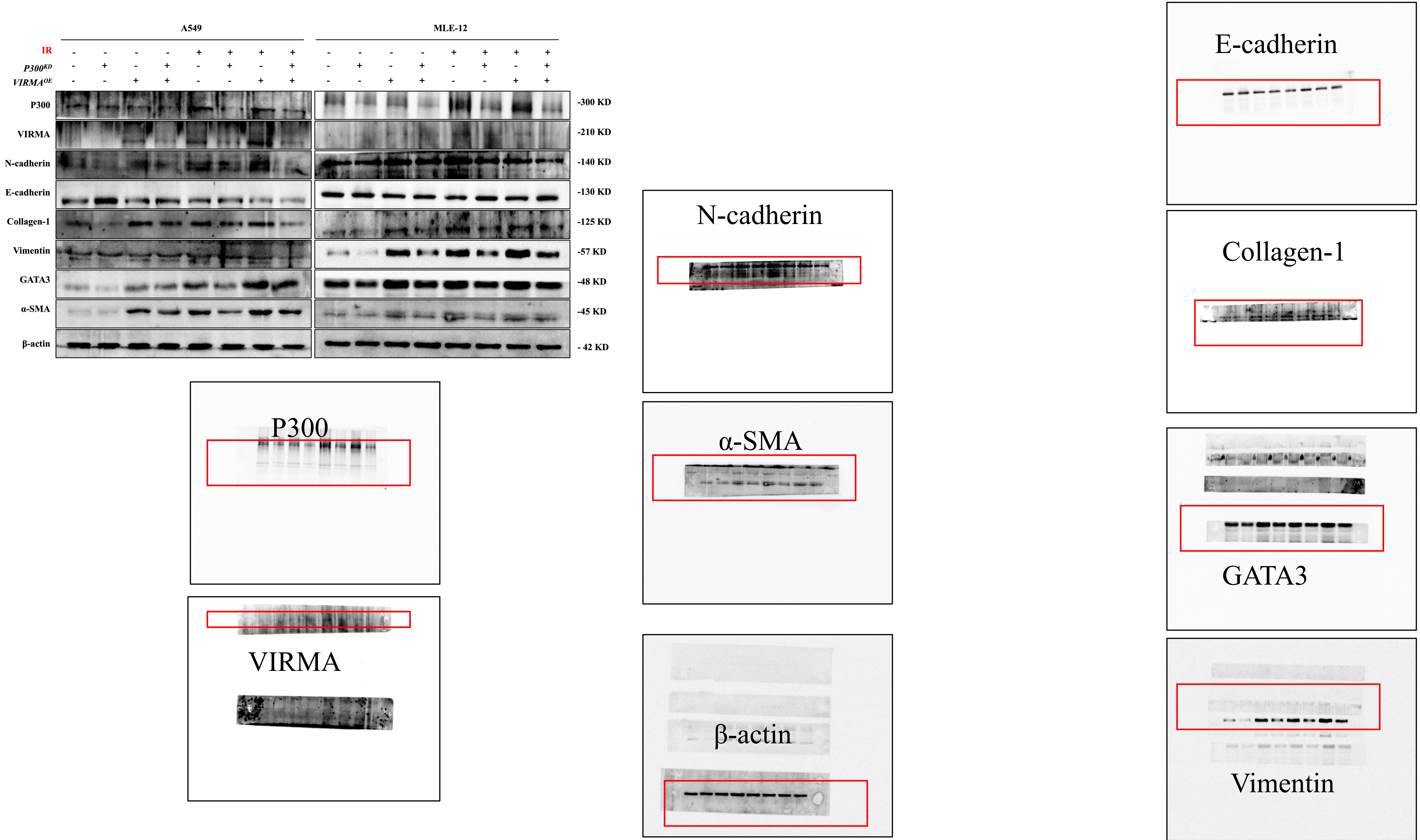
A549
MLE-12
IR
P300KD
VIRMAOE
P300
VIRMA
N-cadherin
E-cadherin
Collagen-1
Vimentin
GATA3
α-SMA
β-actin
-300 KD
-210 KD
-140 KD
-130 KD
-125 KD
-57 KD
-48 KD
-45 KD
- 42 KD
E-cadherin
N-cadherin
Collagen-1
P300
α-SMA
GATA3
VIRMA
β-actin
Vimentin

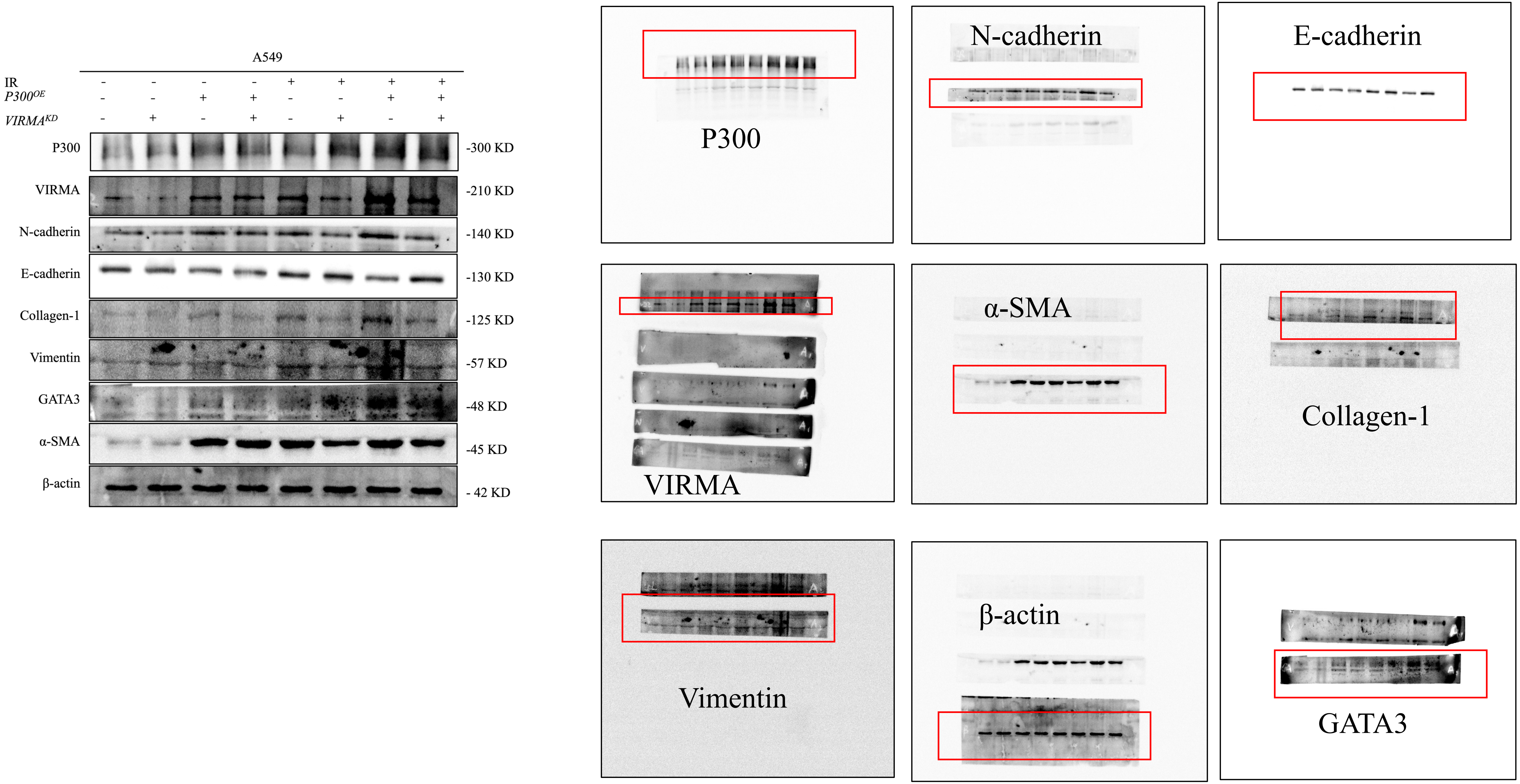

A549
IR - - - - + + + +
P300^OE - - + + - - + +
VIRMA^KD - + - + - + - +
P300
VIRMA
N-cadherin
E-cadherin
Collagen-1
Vimentin
GATA3
α-SMA
β-actin
-300 KD
-210 KD
-140 KD
-130 KD
-125 KD
-57 KD
-48 KD
-45 KD
- 42 KD
P300
N-cadherin
E-cadherin
VIRMA
α-SMA
Collagen-1
Vimentin
β-actin
GATA3

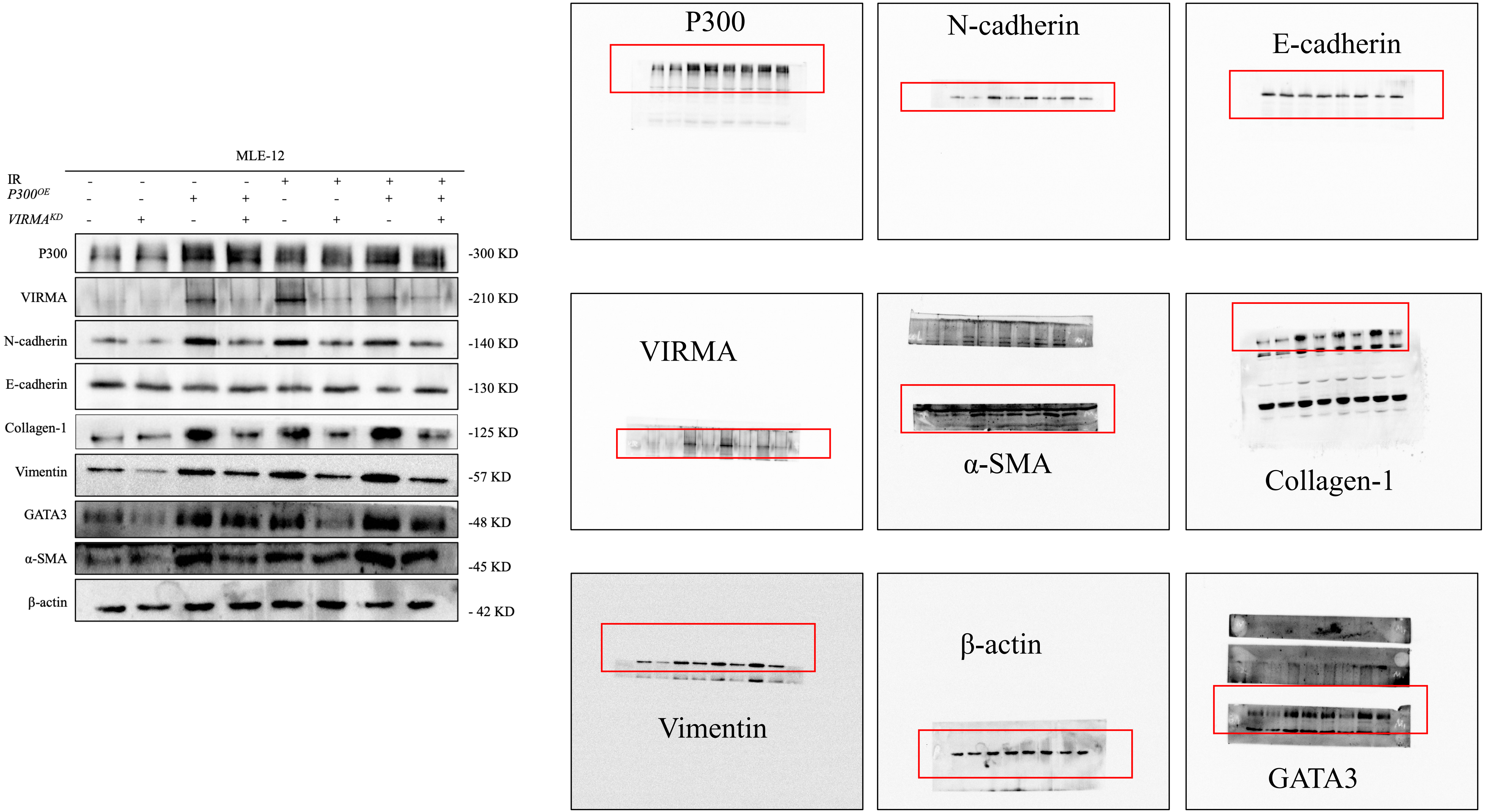
MLE-12
IR
$P300^{OE}$
$VIRMA^{KD}$
P300
VIRMA
N-cadherin
E-cadherin
Collagen-1
Vimentin
GATA3
α-SMA
β-actin
-300 KD
-210 KD
-140 KD
-130 KD
-125 KD
-57 KD
-48 KD
-45 KD
- 42 KD
P300
N-cadherin
E-cadherin
VIRMA
α-SMA
Collagen-1
Vimentin
β-actin
GATA3

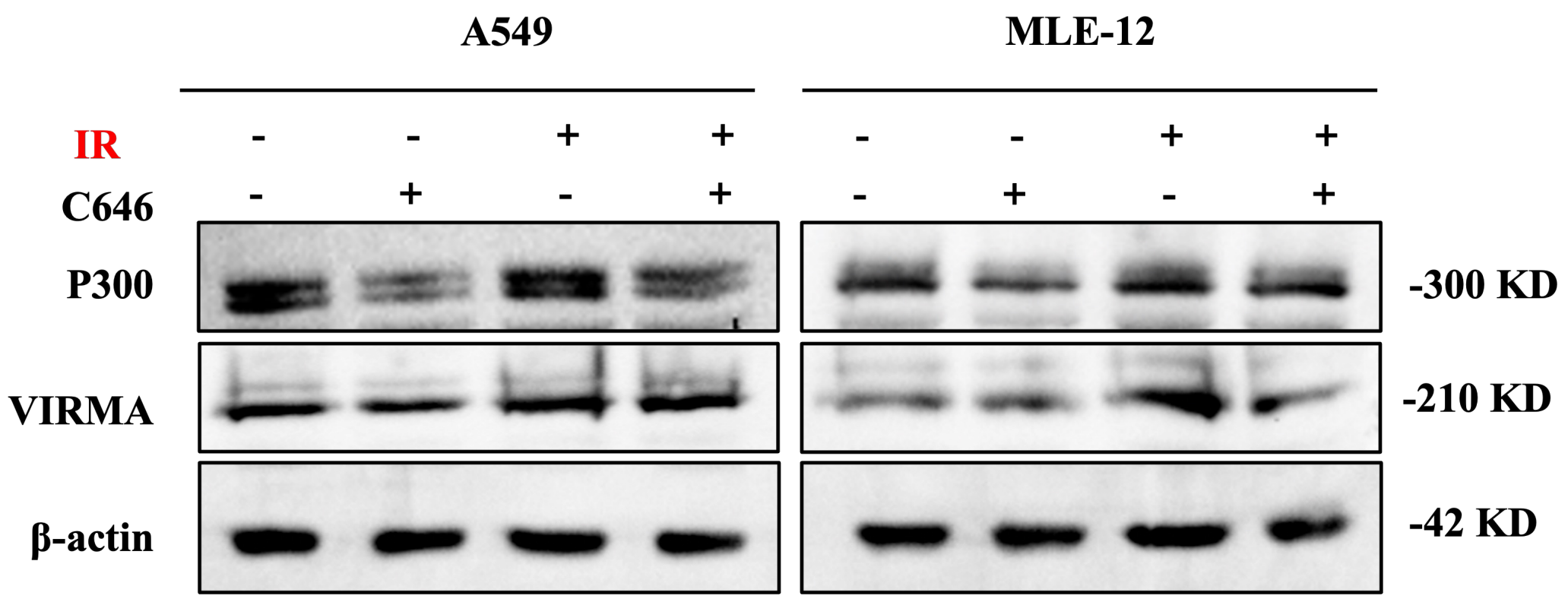

P300

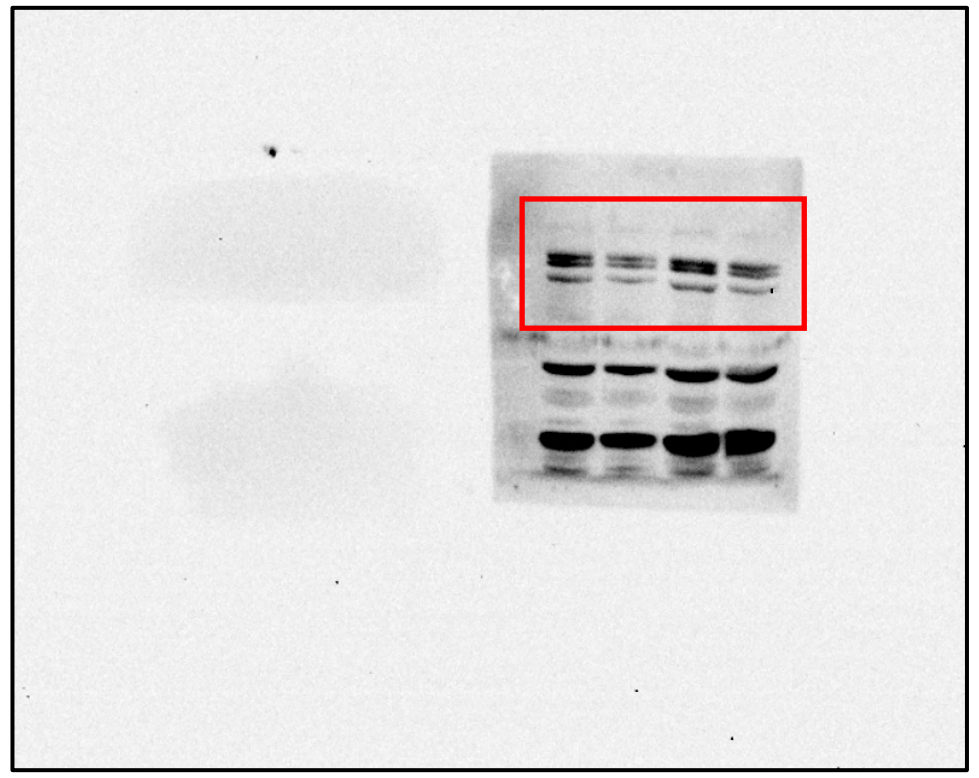

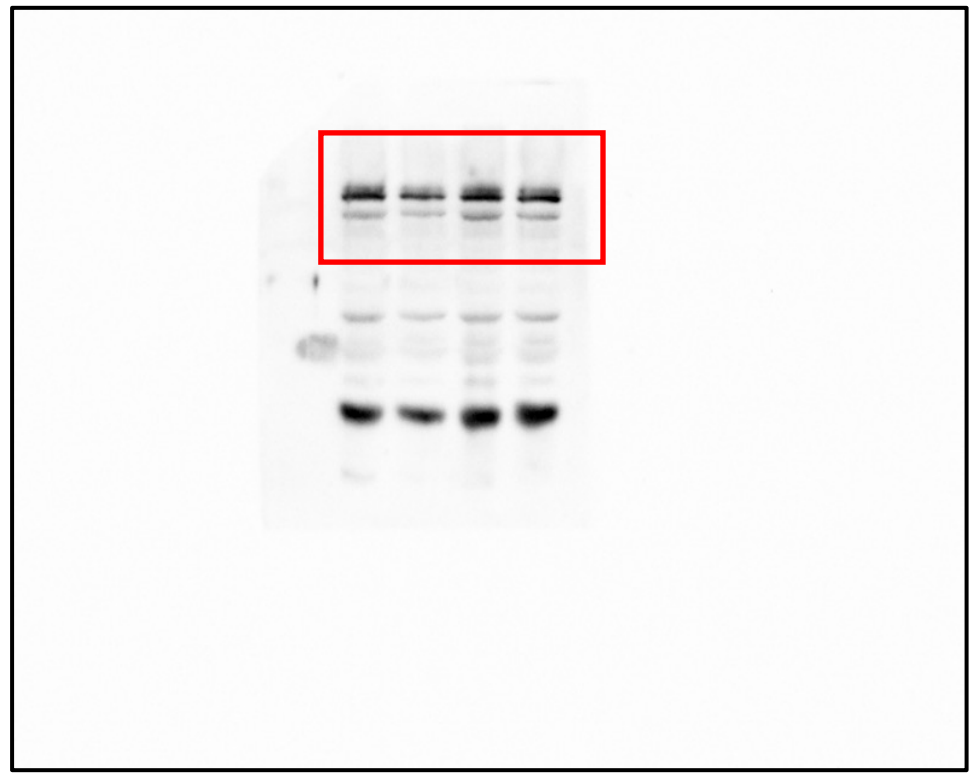

β-actin

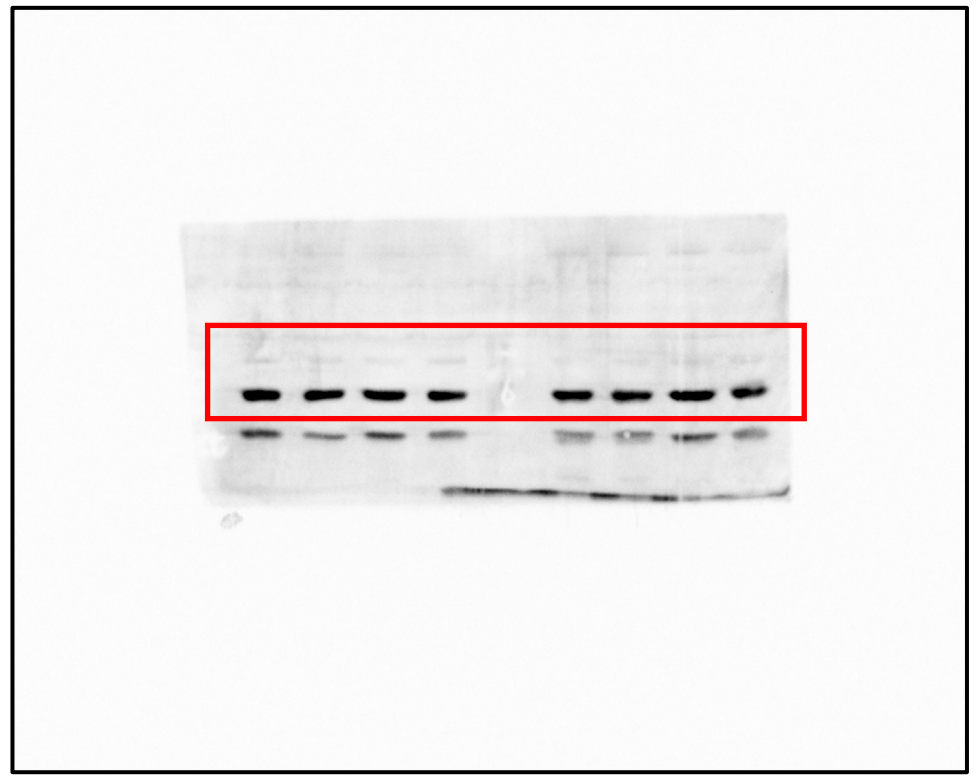

VIRMA

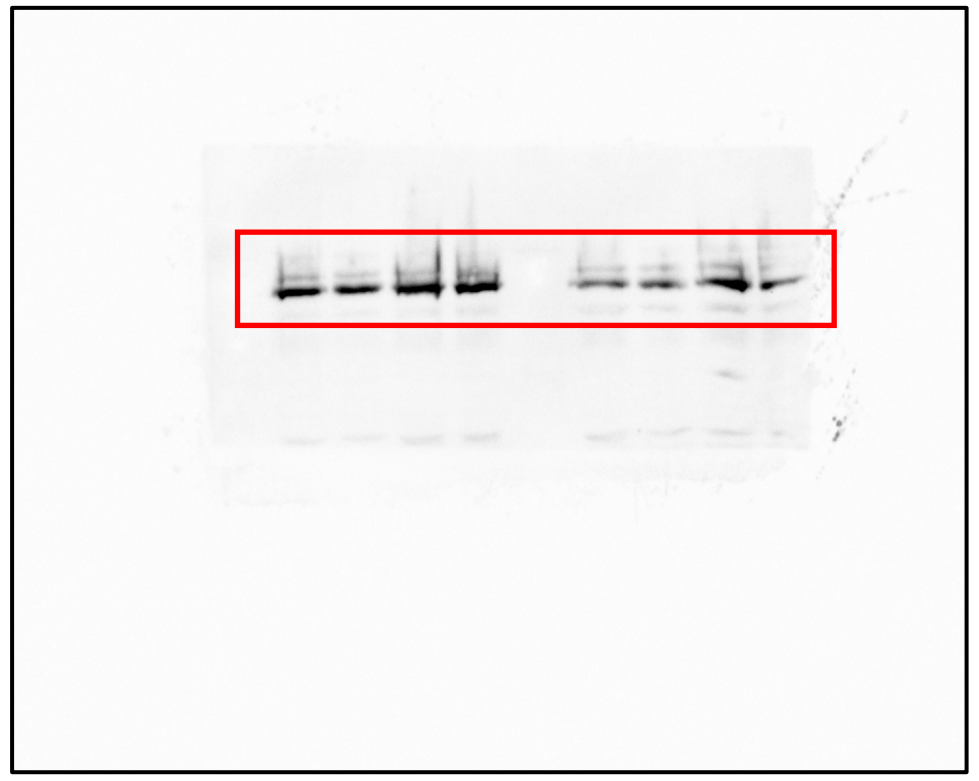

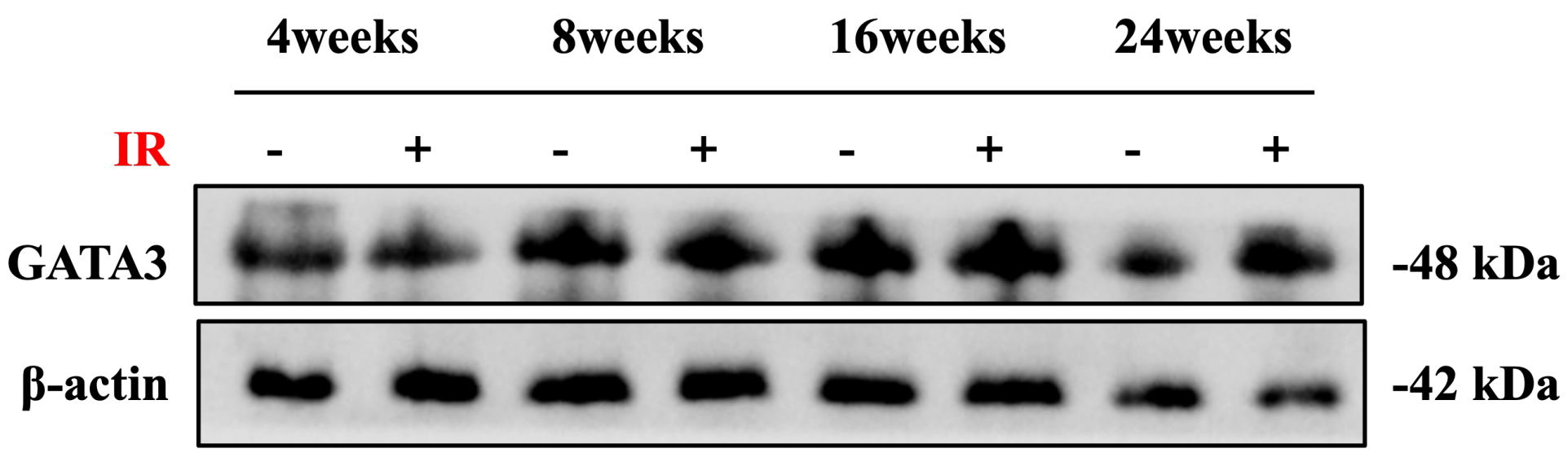
4weeks
8weeks
16weeks
24weeks
IR
- + - + - + - +
GATA3
β-actin
-48 kDa
-42 kDa

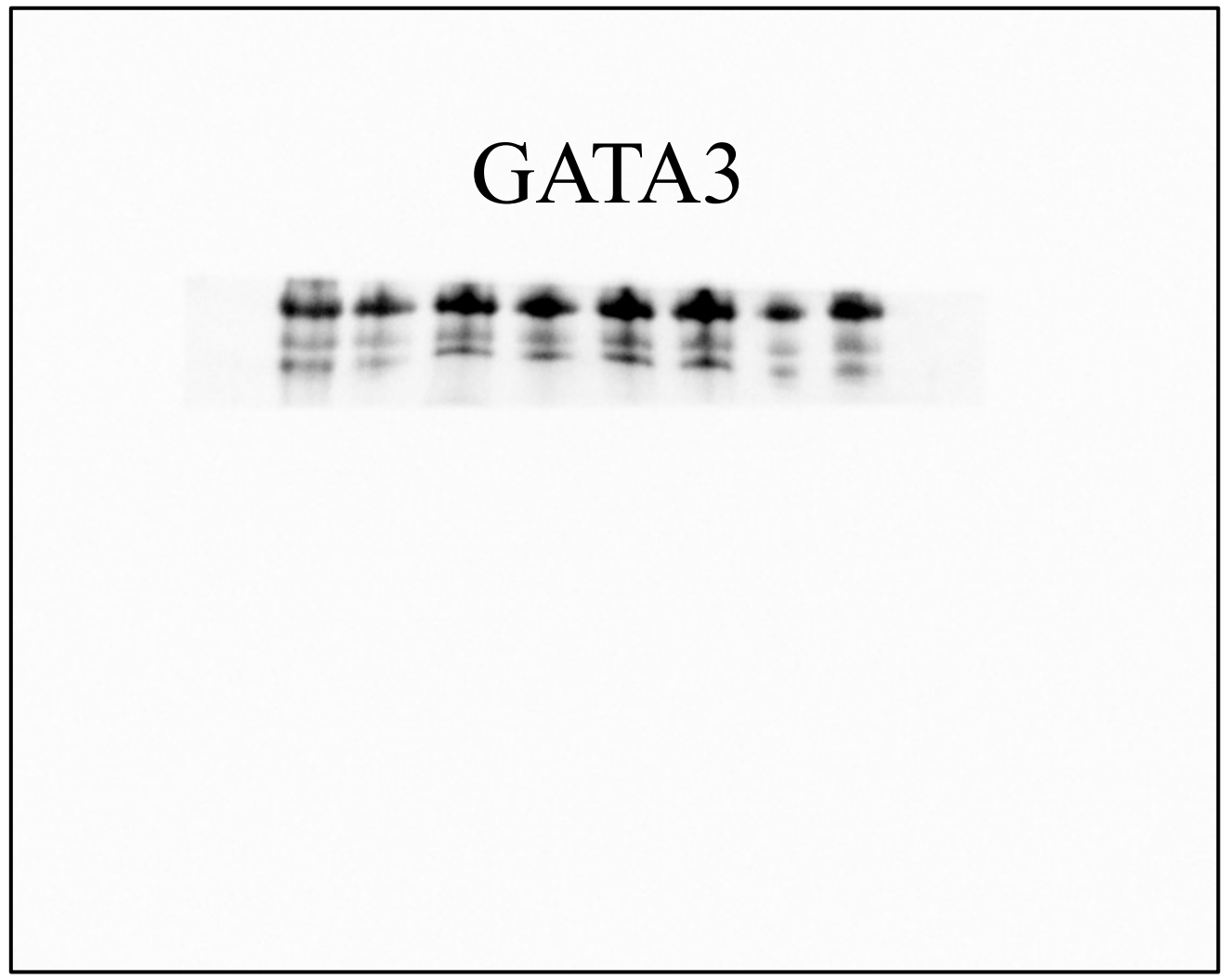
GATA3

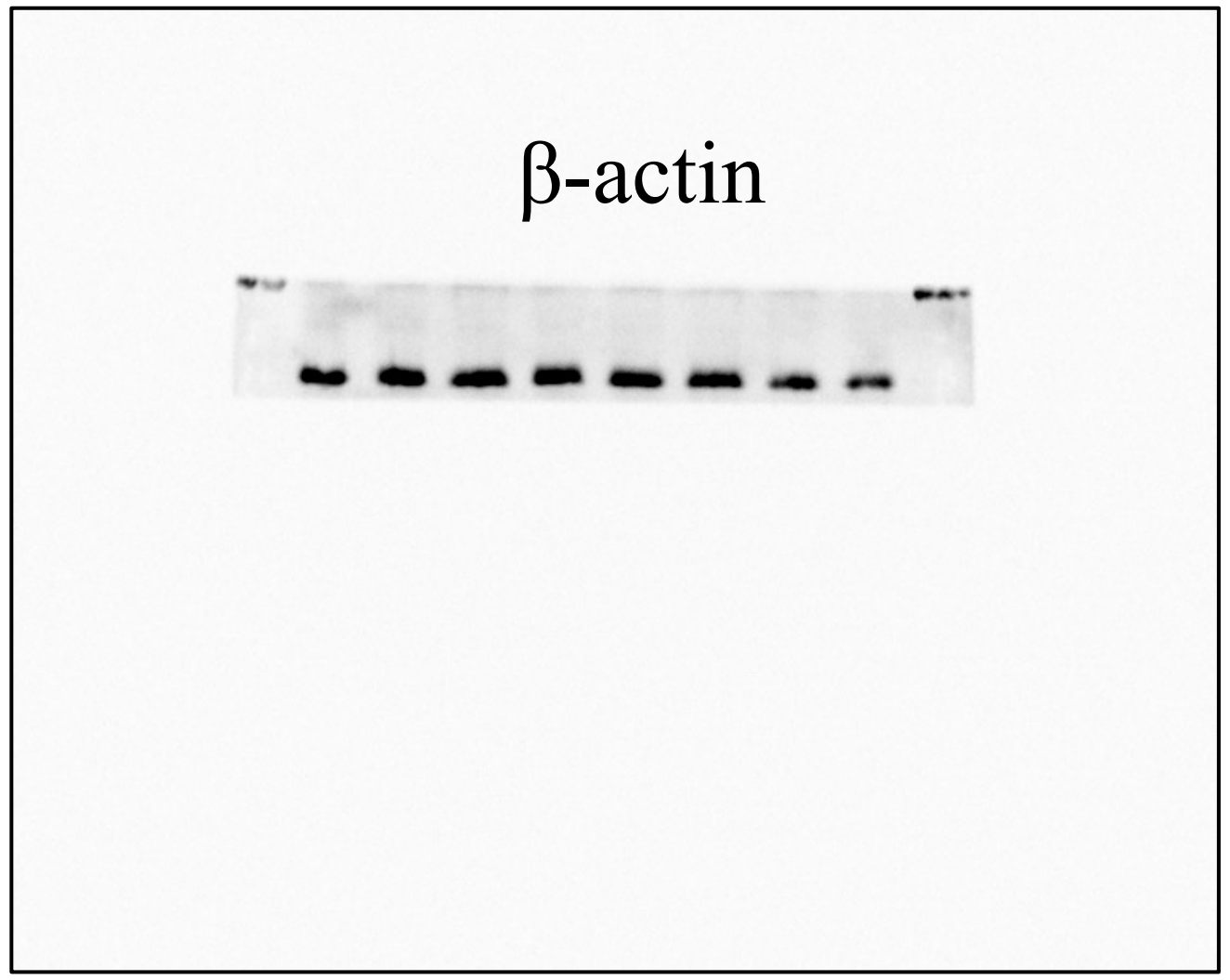
β-actin

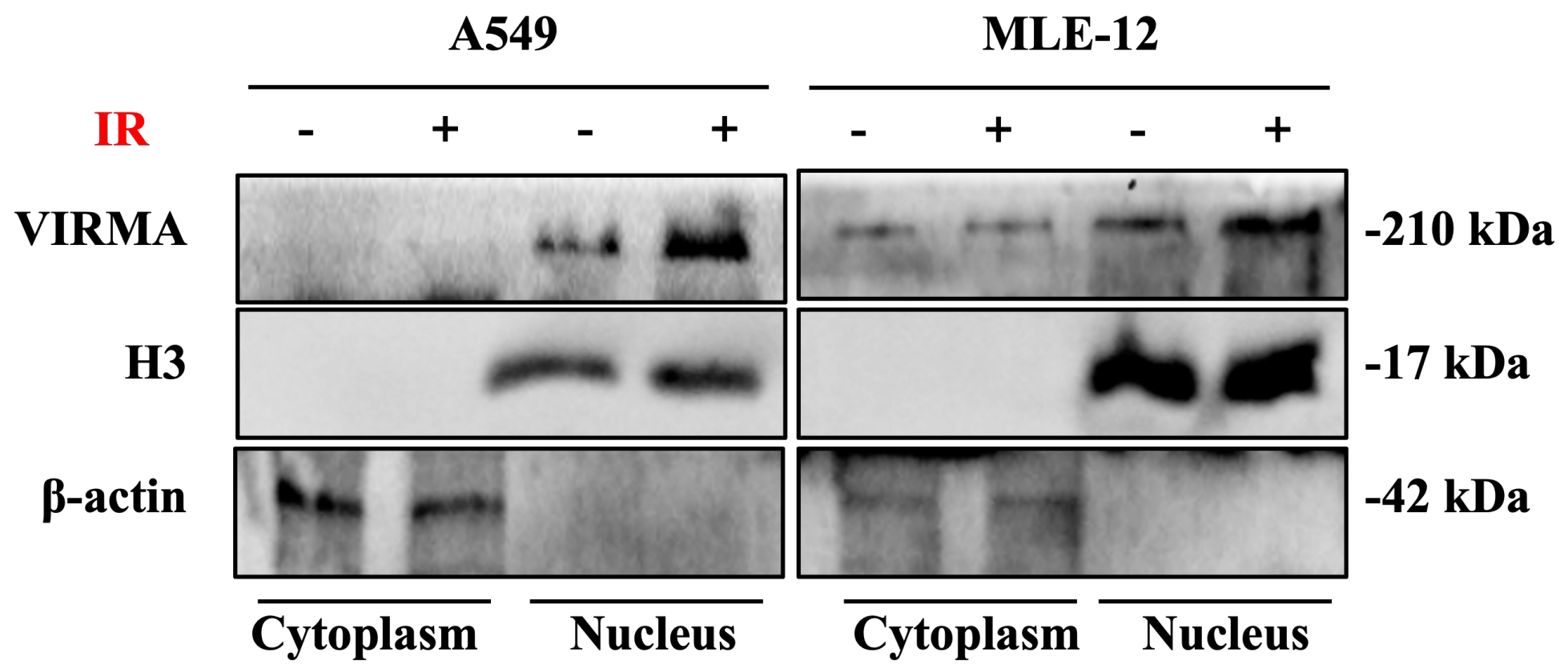
A549
MLE-12
IR
- + - +
- + - +
VIRMA
H3
β-actin
-210 kDa
-17 kDa
-42 kDa
Cytoplasm
Nucleus
Cytoplasm
Nucleus

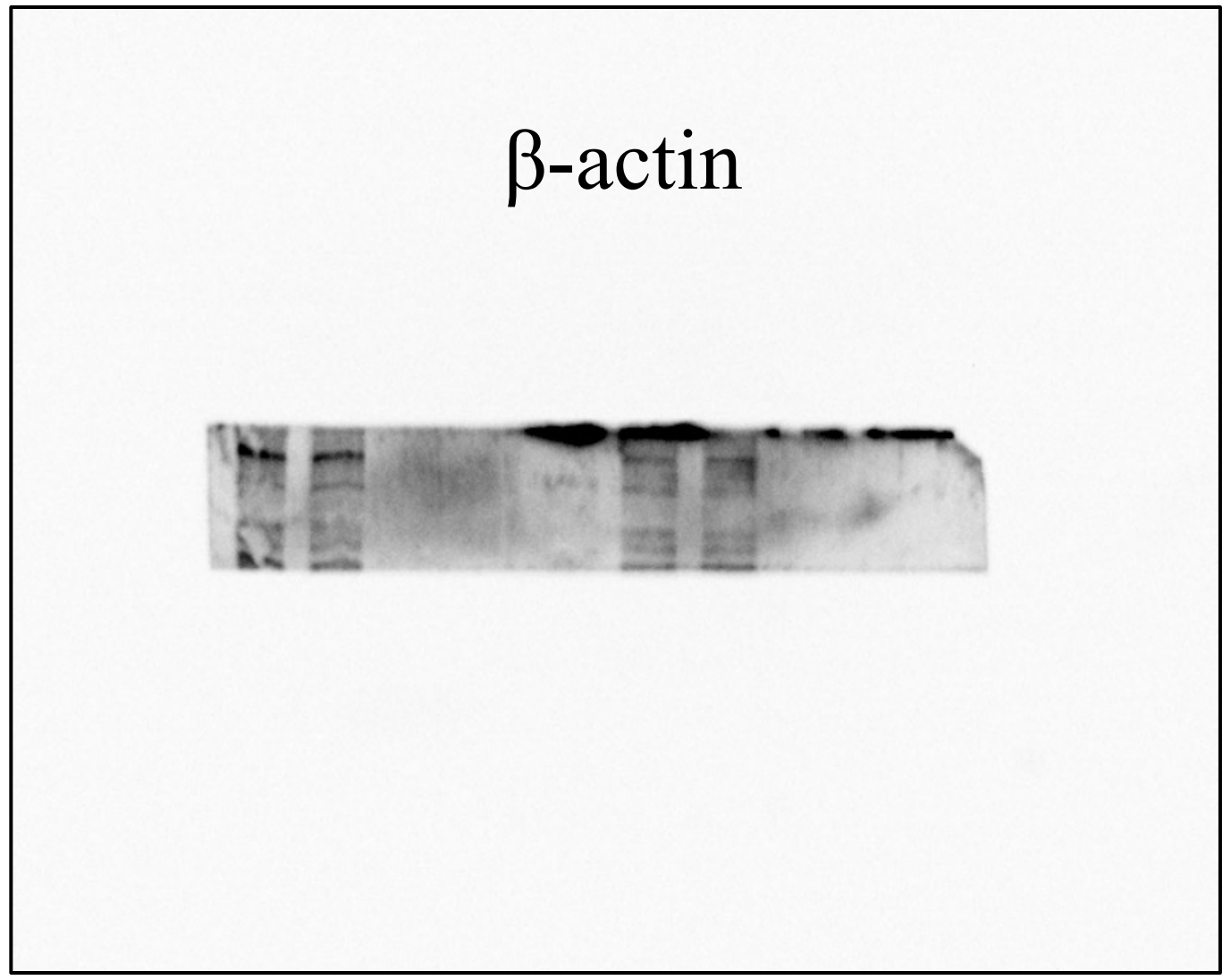
β-actin

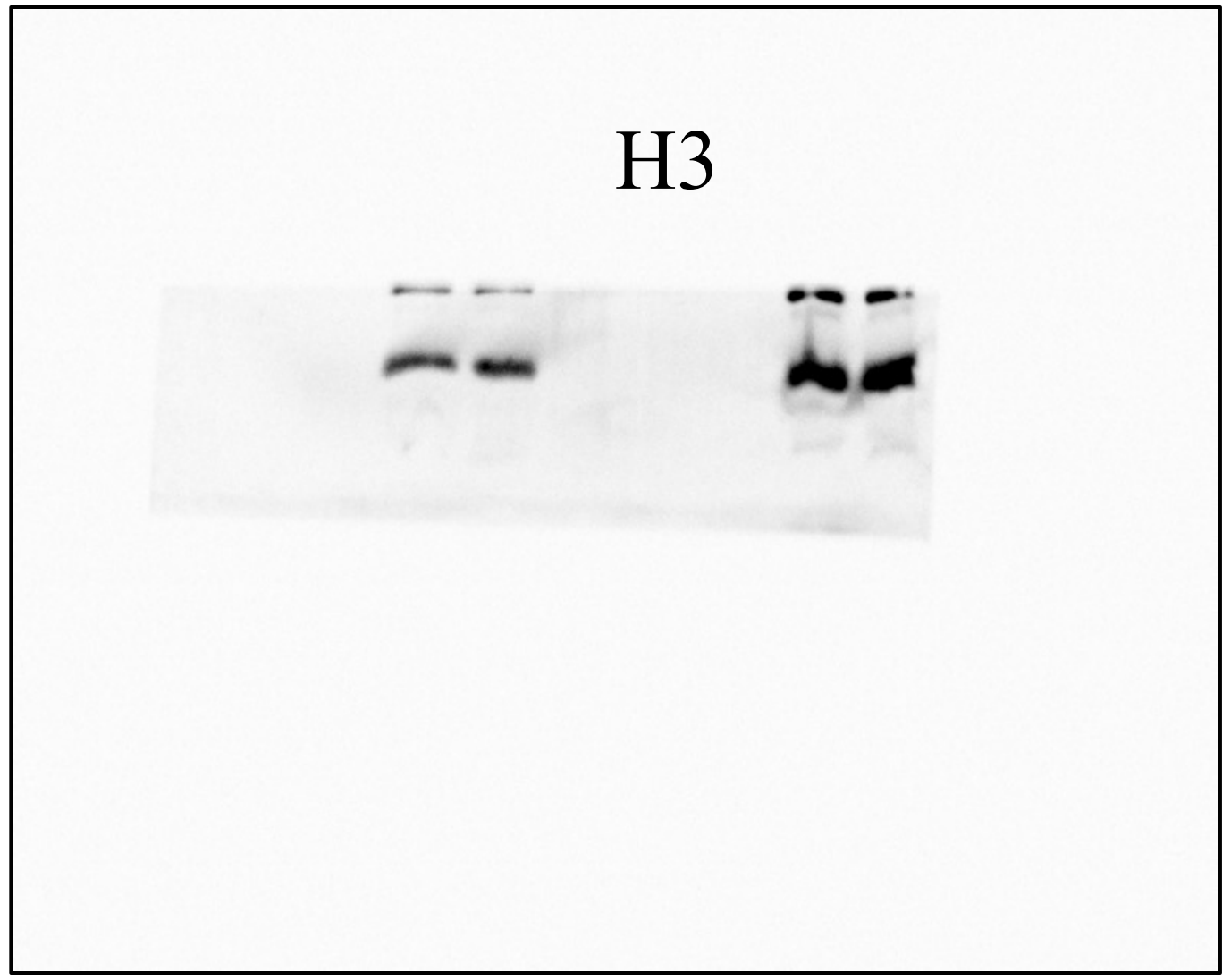
H3

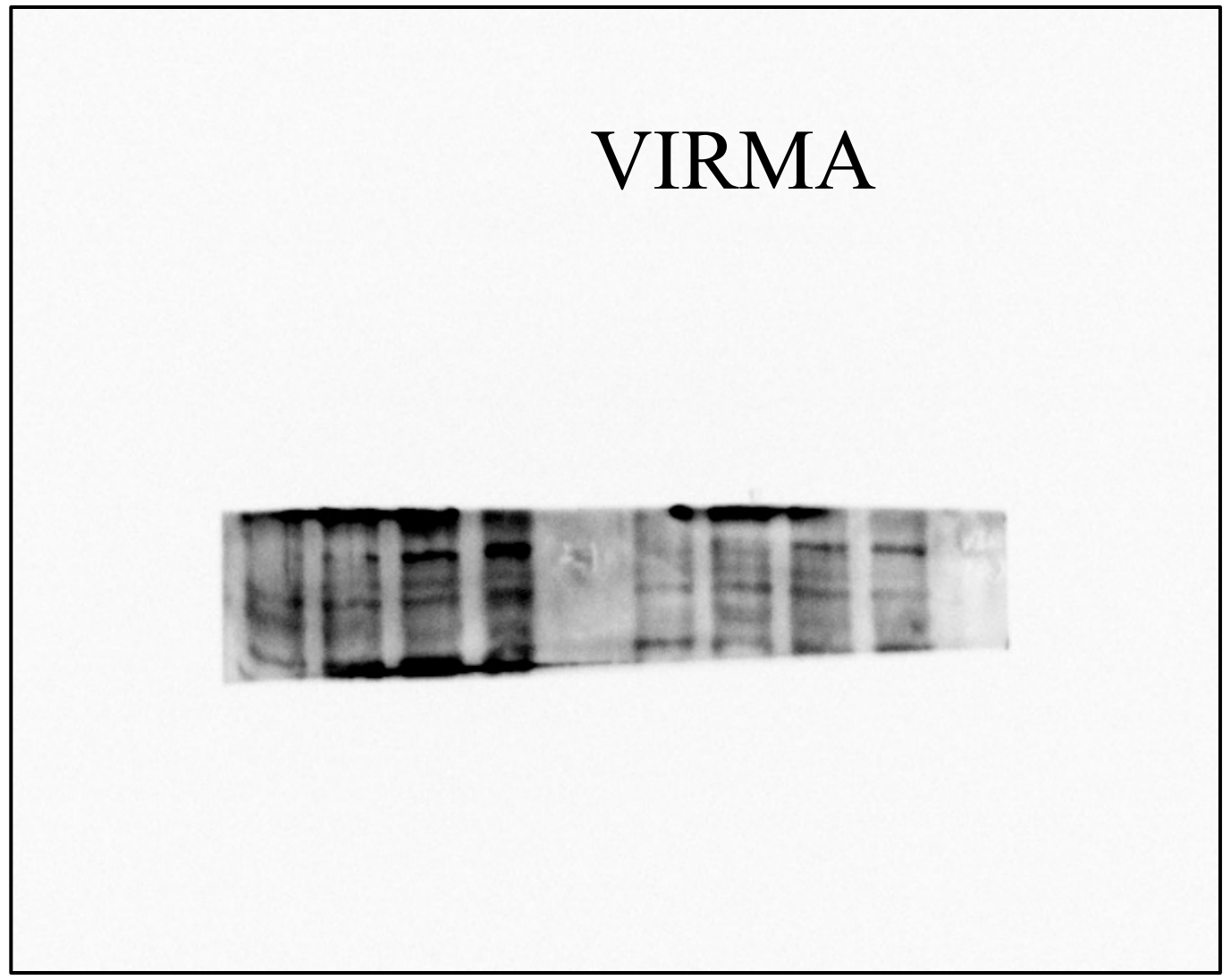
VIRMA

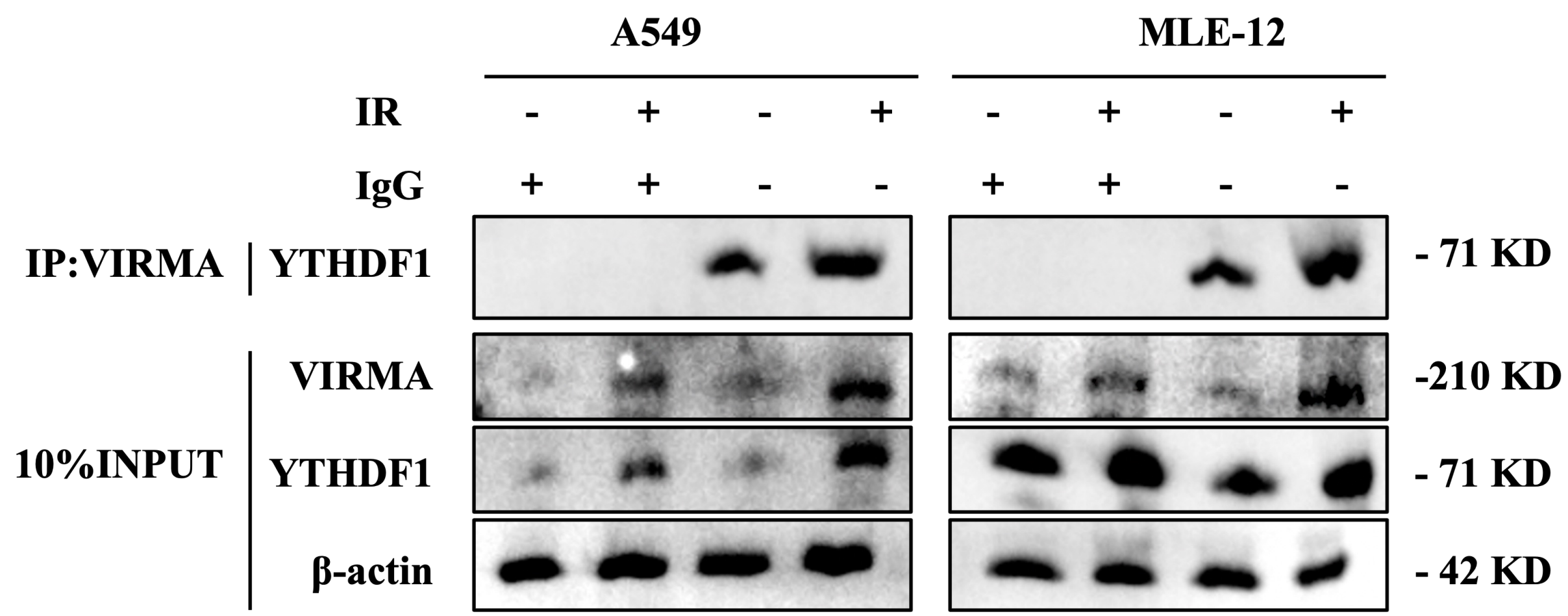
A549
MLE-12
IR - + - + - + - +
IgG + + - - + + - -
IP:VIRMA
YTHDF1
10%INPUT
VIRMA
YTHDF1
β-actin
- 71 KD
-210 KD
- 71 KD
- 42 KD

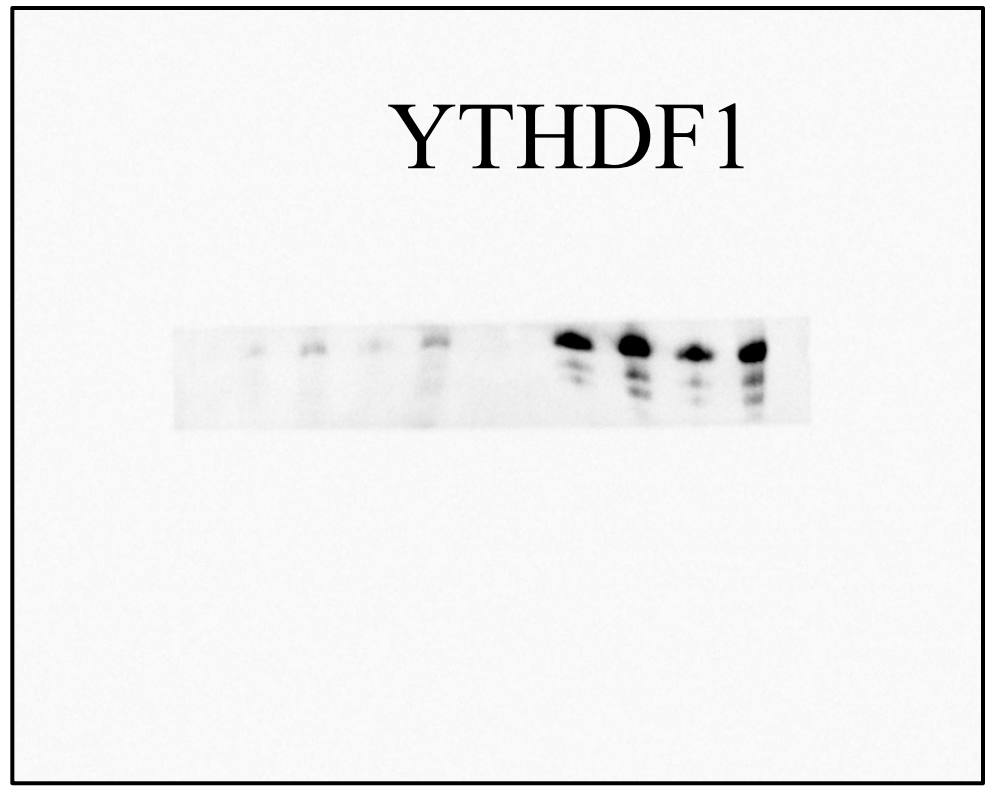
YTHDF1

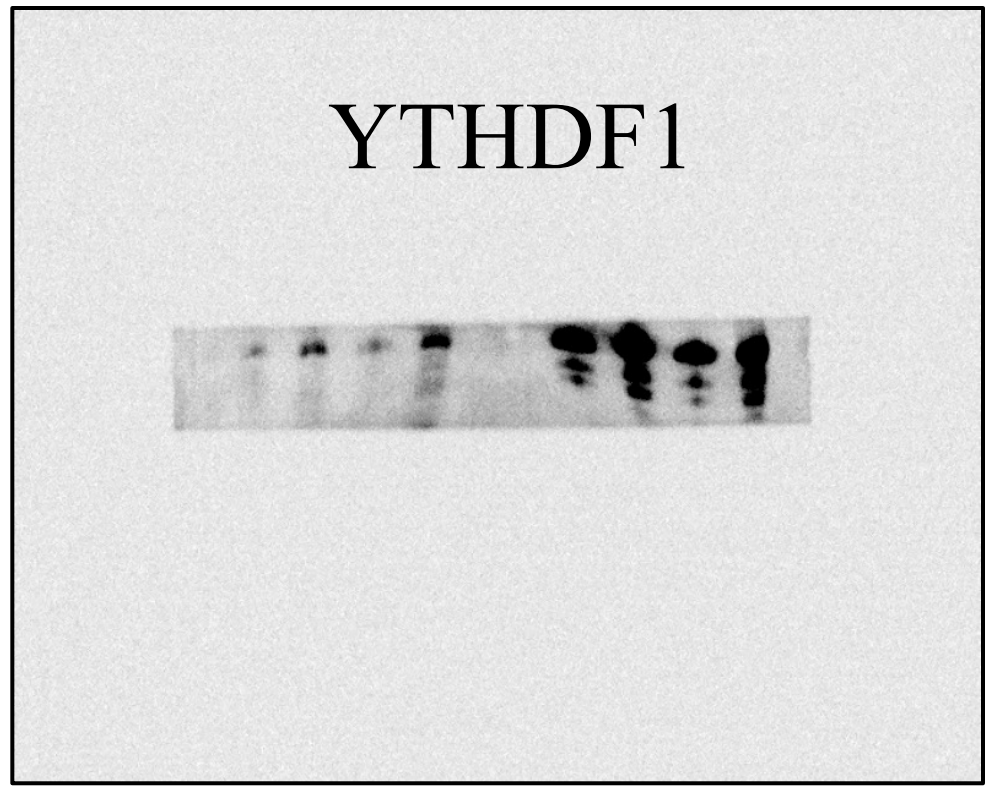
YTHDF1

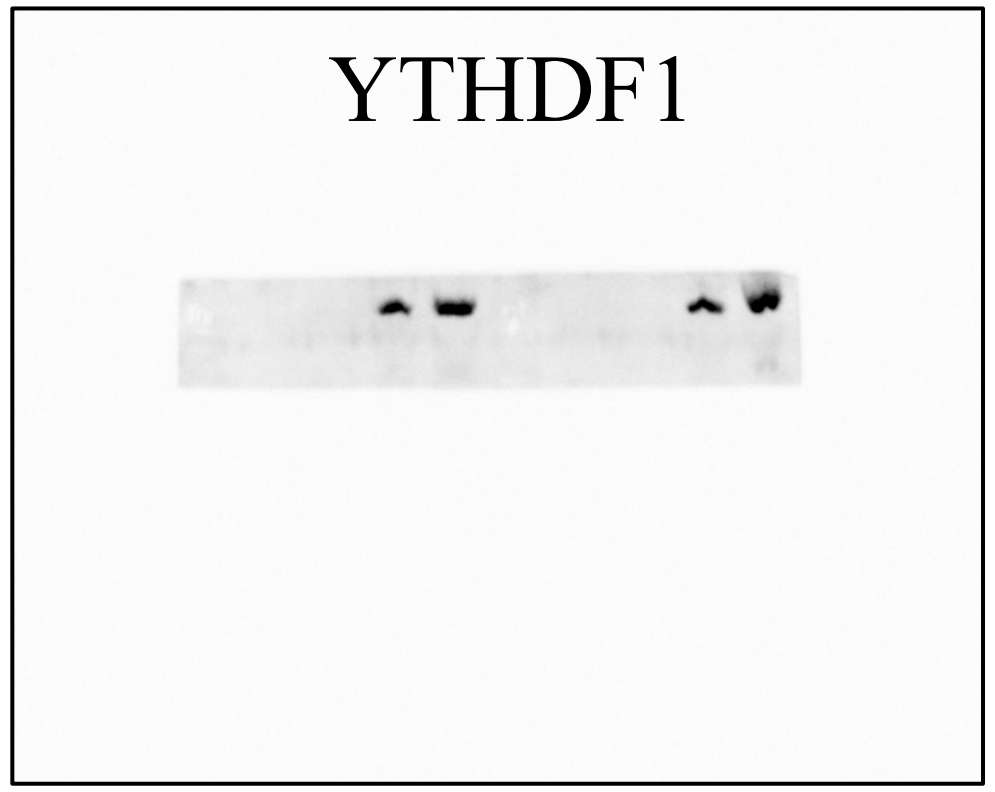
YTHDF1

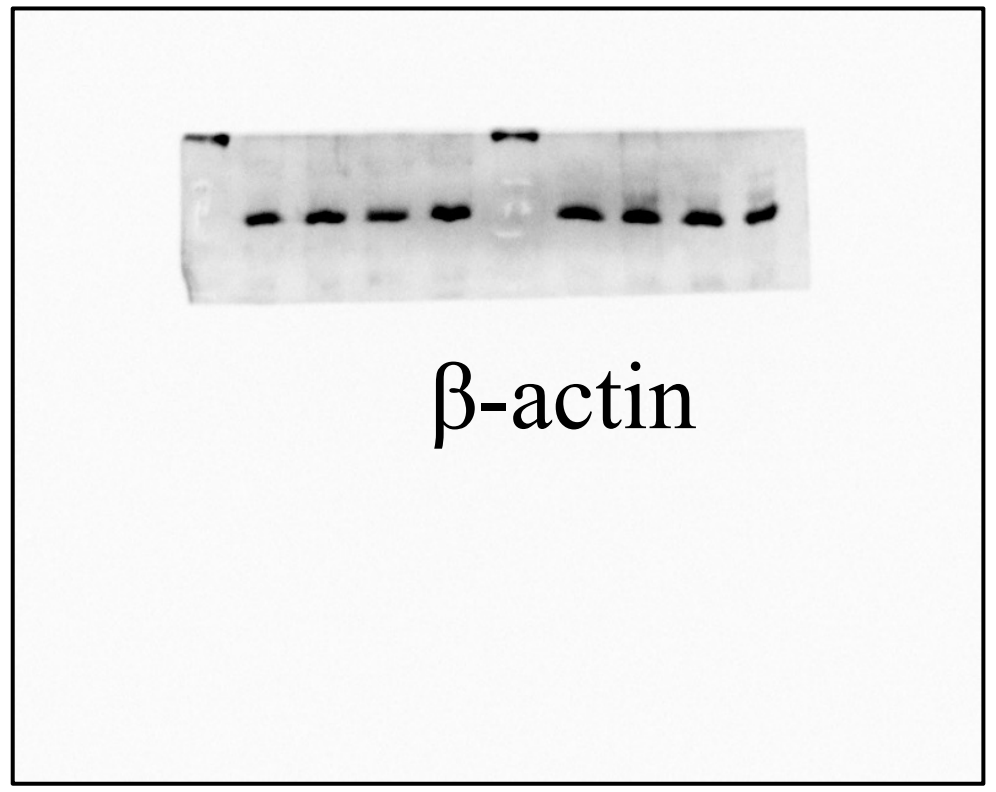
β-actin

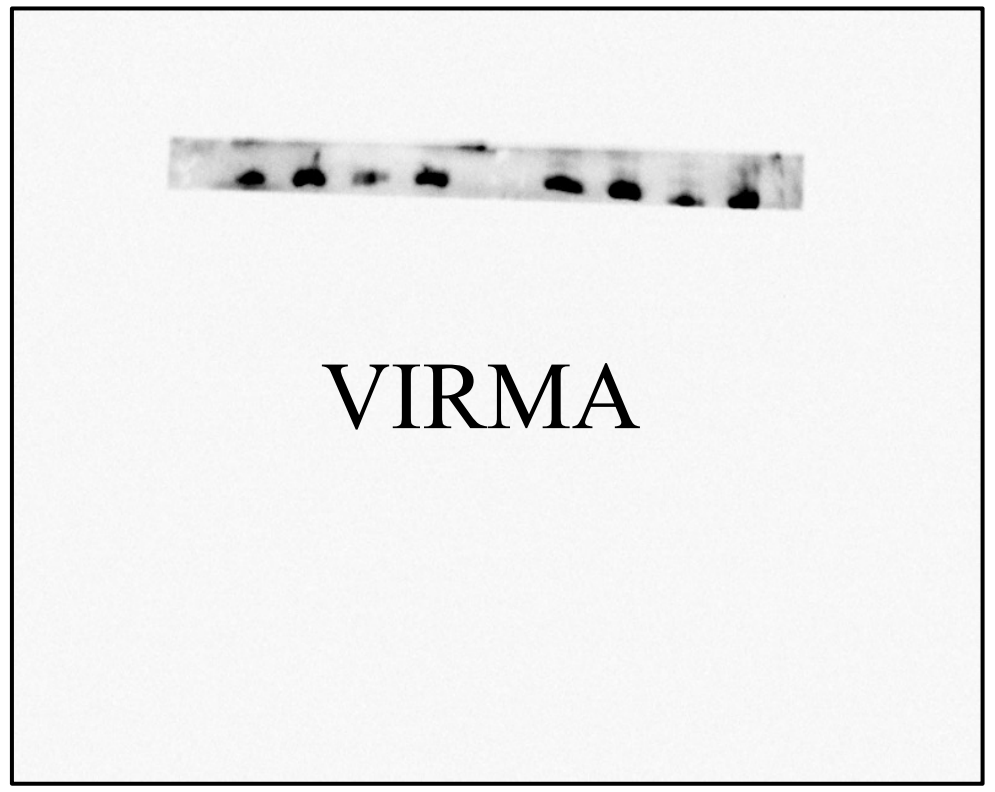
VIRMA

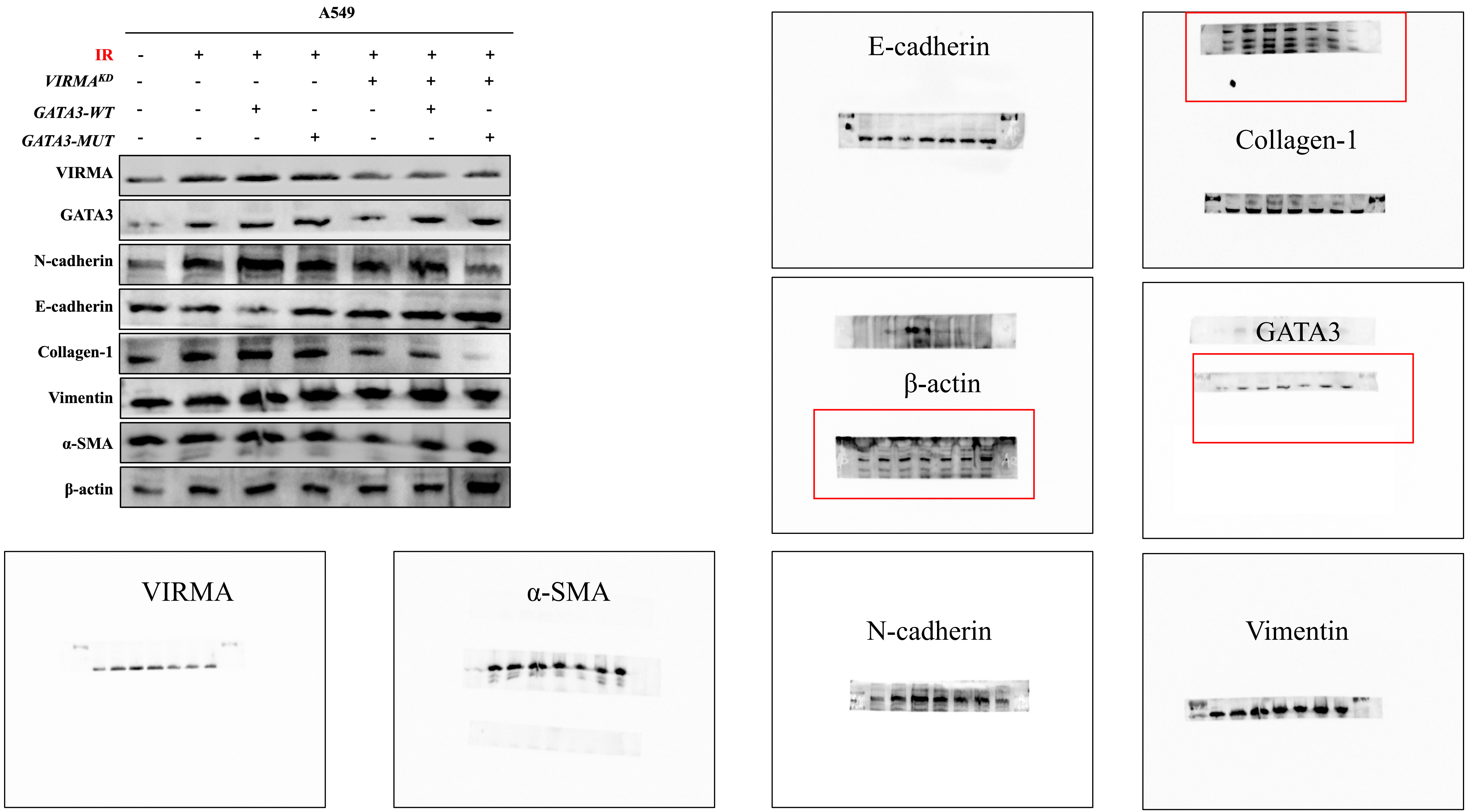
A549
IR - + + + + + +
VIRMA^KD - - - - + + +
GATA3-WT - - + - - + -
GATA3-MUT - - - + - - +
VIRMA
GATA3
N-cadherin
E-cadherin
Collagen-1
Vimentin
α-SMA
β-actin
E-cadherin
Collagen-1
β-actin
GATA3
VIRMA
α-SMA
N-cadherin
Vimentin

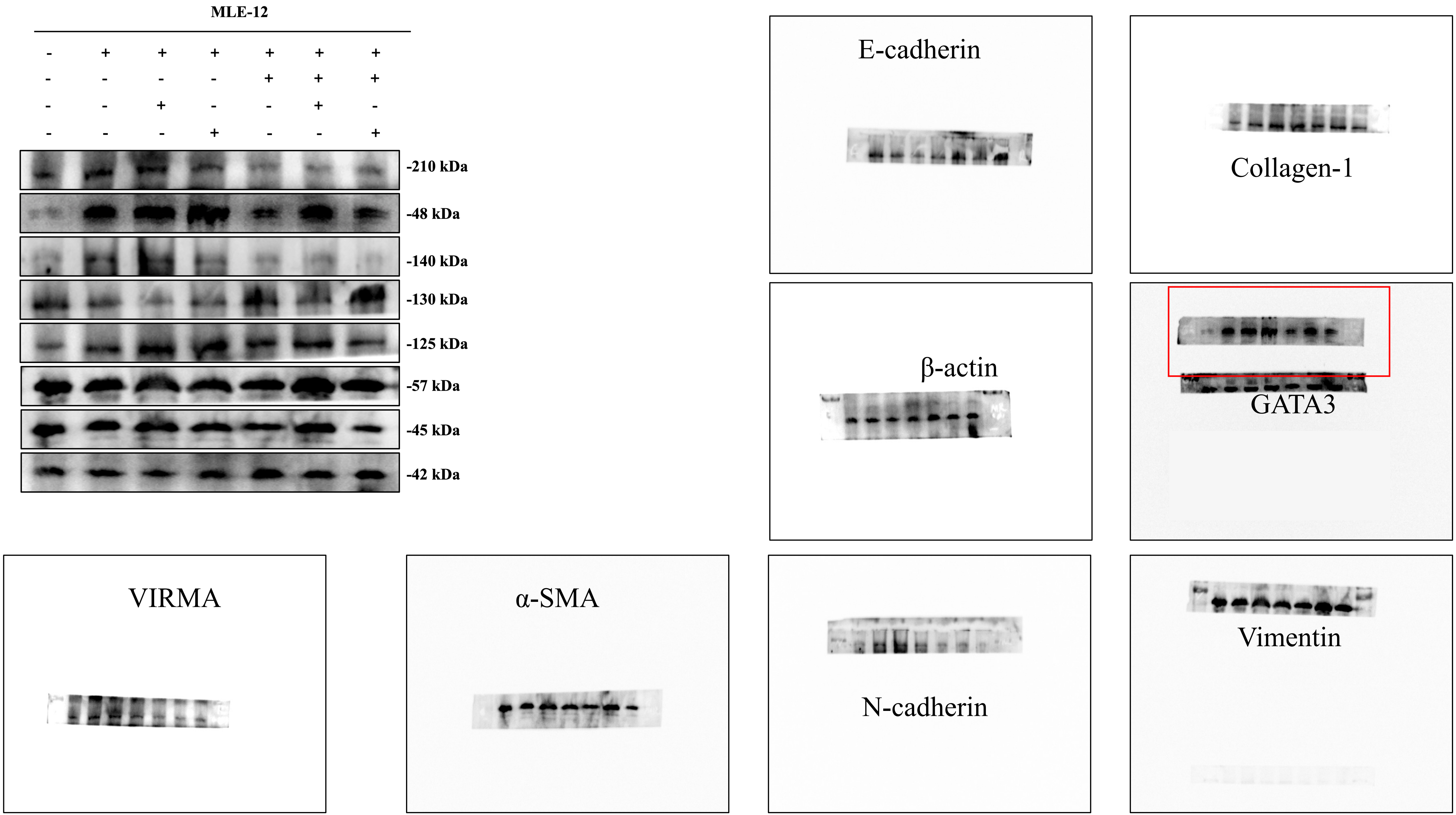
MLE-12
- + + + + + +
- - - - + + +
- - + - - + -
- - - + - - +
-210 kDa
-48 kDa
-140 kDa
-130 kDa
-125 kDa
-57 kDa
-45 kDa
-42 kDa
E-cadherin
Collagen-1
β-actin
GATA3
VIRMA
α-SMA
N-cadherin
Vimentin
