# Supplementary material for: p300‐Mediated H3K18 Lactylation Drives Radiation‐Induced Pulmonary Fibrosis via VIRMA‐Dependent m6A Modification of GATA3
Source: Adv Sci (Weinh). 2026 Aug 24;13(63):e77328. doi: 10.1002/advs.77328 (PMC13502275; doi:10.1002/advs.77328)
Supplement: Supplementary file 2 [file ADVS-13-e77328-s001.docx]

**Animal experimentation ethical license certificate**
