# Supplementary material for: p300‐Mediated H3K18 Lactylation Drives Radiation‐Induced Pulmonary Fibrosis via VIRMA‐Dependent m6A Modification of GATA3
Source: Adv Sci (Weinh). 2026 Aug 24;13(63):e77328. doi: 10.1002/advs.77328 (PMC13502275; doi:10.1002/advs.77328)
Supplement: Supplementary file 4 [file ADVS-13-e77328-s005.docx]

Table S1. The sequences of the si-RNAs oligonucleotides and the sh-RNA.

| m-si-NC | sense | UUCUCCGAACGUGUCACGUTT |
| --- | --- | --- |
|  | antisense | ACGUGACACGUUCGGAGAATT |
| m-si-GATA3 | sense | CCUGUGGGCUGUACUACAATT |
|  | antisense | UUGUAGUACAGCCCACAGGTT |
| h-sh-NC | sense | GTTCTCCGAACGTGTCACGTT |
| h-sh-VIRMA-1 | sense | GGATCAAGGTCCAGATAAA |
| h-sh-VIRMA-2 | sense | GGTAACAGCTTTAGAAGAA |
| h-sh-VIRMA-3 | sense | GGATAGTGATGAACAGAAA |
| m-sh-NC |  | GTTCTCCGAACGTGTCACGTT |
| m-sh-VIRMA | sense | TAATGACACTGTCCAAAGC |
| PmirGLO-GATA3-WT | sense | ATGACTTTATTGCATCTGGGTA  GCTGTAAGGCATGAAGGATGC  CAAGAAGTTTAAGGAATATGG  GAGAAATAGTGTGGAAATTAA  GAAGAAACTAGGTCTGATATTC  AAAT**GGAC**AAACTGCCAGTTTT  GTTTCCTTTCACTGGCCACAGT  TGTTTGATGCATTAAAAGAAAAT  AAAAAA |
| PmirGLO-GATA3-MUT | sense | ATGACTTTATTGCATCTGGGTA  GCTGTAAGGCATGAAGGATGC  CAAGAAGTTTAAGGAATATGG  GAGAAATAGTGTGGAAATTAA  GAAGAAACTAGGTCTGATATTC  AAAT**GGCC**AAACTGCCAGTTTT  GTTTCCTTTCACTGGCCACAGT  TGTTTGATGCATTAAAAGAAAAT  AAAAAA |

Table S2. The sequences of the qRT-PCR primers and MeRIP-qPCR-primers.

| m-β-actin | Forward | GGCTGTATTCCCCTCCATCG |
| --- | --- | --- |
|  | Reverse | CCAGTTGGTAACAATGCCATGT |
| m-N-cadherin | Forward | AGCGCAGTCTTACCGAAGG |
|  | Reverse | TCGCTGCTTTCATACTGAACTTT |
| m-E-cadherin | Forward | CAGGTCTCCTCATGGCTTTGC |
|  | Reverse | CTTCCGAAAAGAAGGCTGTCC |
| m-GATA3 | Forward | CCCCATTACCACCTATCCGC |
|  | Reverse | CCTCGACTTACATCCGAACCC |
| m-VIRMA | Forward | CTTGGCAAGTGGCTTGAACC |
|  | Reverse | ACGTAAGGCAGTGGTAAGGC |
| m-ACTA2 | Forward | GGACGTACAACTGGTATTGTGC |
|  | Reverse | TCGGCAGTAGTCACGAAGGA |
| m-collagen-1 | Forward | TAAGGGTCCCCAATGGTGAGA |
|  | Reverse | GGGTCCCTCGACTCCTACAT |
| m-intergenic region | Forward | AAGGGGCCTCTGCTTAAAAA |
|  | Reverse | AGAGCTCCATGGCAGGTAGA |
| m-VIRMA-promoter | Forward | ACTGCTTGTCAGCCAGAAATA |
|  | Reverse | AGAAAGAGGGAGGGAGACAA |
| h-GAPDH | Forward | ACGGATTTGGTCGTATTGGG |
|  | Reverse | TGATTTTGGAGGGATCTCGC |
| h-N-cadherin | Forward | GCCATCAAGCCTGTGGGAAT |
|  | Reverse | GGAGCCACTGCCTTCATAGT |
| h-E-cadherin | Forward | AAAGGCCCATTTCCTAAAAACCT |
|  | Reverse | TGCGTTCTCTATCCAGAGGCT |
| h-Vimentin | Forward | TACAGGAAGCTGCTGGAAGG |
|  | Reverse | ACCAGAGGGAGTGAATCCAG |
| h-GATA3 | Forward | GCCCCTCATTAAGCCCAAG |
|  | Reverse | TTGTGGTGGTCTGACAGTTCG |
| h-VIRMA | Forward | TACTTTGAGCCCATTTCTCCTGA |
|  | Reverse | GGAATACTGTCTACTGTTCGTCG |
| h-ACTA2 | Forward | GGCAAGTGATCACCATCGGA |
|  | Reverse | GTGGTTTCATGGATGCCAGC |
| h-collagen-1 | Forward | GAGGGCCAAGACGAAGACATC |
|  | Reverse | CAGATCACGTCATCGCACAAC |
| h-FTO | Forward | AACACCAGGCTCTTTACGGTC |
|  | Reverse | TGTCCGTTGTAGGATGAACCC |
| h-intergenic region | Forward | GATCTAGCTAGCTAGCTAGC |
|  | Reverse | CTAGCTAGCTAGCTAGCTAG |
| h-VIRMA-promoter | Forward | TATATTGCCCAGGCAGTTCTC |
|  | Reverse | CAGTGCTATGTTTACTAGCATGAAA |
| MeRIP-qPCR-GATA3 | Forward | TCTGACAGTTCGCACAGGAC |
|  | Reverse | CCTGCAGTCCCTTTCGACTT |

Table S3. A list of all antibodies used in this work and dilutions.

| Antibodies | Cat. | Company | Dilutions |
| --- | --- | --- | --- |
| Anti-β-actin | AP0060 | Bioworld | 1:1000 |
| Anti-VIRMA | NBP2-93159 | NOVUS | 1:1000 |
| Anti-YTHDF1 | NBP2-94472 | NOVUS | 1:1000 |
| Anti-α-SMA | BS70000 | Bioworld | 1:1000 |
| Anti-Collagen-1 | BS1530 | Bioworld | 1:1000 |
| Anti-N-cadherin | BS72312 | Bioworld | 1:1000 |
| Anti-E-cadherin | BS1098 | Bioworld | 1:1000 |
| Anti-Vimentin | BS1491 | Bioworld | 1:1000 |
| Anti-GATA3 antibody | ab199428 | Abcam | 1:1000 |
| Goat anti-Mouse IgG (H+L) HRP | BS12478 | Bioworld | 1:50000 |
| Goat anti-Rabbit IgG (H+L) HRP | BS13278 | Bioworld | 1:10000 |
| Cy3 Goat Antibody | AS007 | ABclonal | 1:250 |
| Normal Rabbit IgG | 2729S | CST | 1:100 |
