# Supplementary material for: p300‐Mediated H3K18 Lactylation Drives Radiation‐Induced Pulmonary Fibrosis via VIRMA‐Dependent m6A Modification of GATA3
Source: Adv Sci (Weinh). 2026 Aug 24:e77328. Online ahead of print. doi: 10.1002/advs.77328 (PMC13502275; doi:10.1002/advs.77328)
Supplement: Supplementary file 2 [file ADVS-9999-e77328-s001.docx]

**Supplementary Fig.1 Radiation-induced pulmonary fibrosis disrupts m6A homeostasis.**

A: The expression of HYP in RIPF mice model tissue. B: The mRNA level of ACTA2 and Collagen-1 in RIPF mice model tissue. C: Immunohistochemistry was used to detect the expression of α-SMA protein in RIPF mice model tissue. D: Immunohistochemistry was used to detect the expression of Collagen-1 protein in RIPF mice model tissue. E: The mRNA level of ACTA2 and Collagen-1 in the RIPF cell model. F: Gene ontology analysis of hyper-methylated m6A transcripts. G:GGAC was detected as the predominant consensus motif in the control and RIPF cell model. The data are presented as the mean ± SD (n= 3 or 5), **P*<0.05 vs. control, ** *P* <0.01 vs. control.

**Supplementary Fig.2 High levels of GATA3 promote the RIPF process**

A: The protein expression of GATA3 in RIPF mice model tissue by western blotting. B: The protein expression of GATA3 in RIPF cell model by western blotting. C: Immunohistochemistry was used to detect the expression of GATA3 protein in RIPF mice model tissue. D: The mRNA level of EMT and ECM marker after GATA3 knockdown. E: The protein expression of EMT and ECM marker after GATA3 overexpression. F: The mRNA level of EMT and ECM marker after GATA3 overexpression. The data are presented as the mean ± SD (n= 3), **P*<0.05 vs. control, ** *P* <0.01 vs. control, ^#^*P*<0.05 vs IR , ^##^ *P* <0.01 vs IR.

**Supplementary Fig.**3 VIRMA-mediated m6A modification exhibits tissue- and cell-specificity

A: VIRMA Expression in Single-Cell Sequencing of Lung Tissue. B: GATA3 Expression in Single-Cell Sequencing of Lung Tissue. C: Expression levels of VIRMA and GATA3 in the lung fibrosis database. D: Correlation of VIRMA and GATA3 in the lung fibrosis database. E: Expression levels of VIRMA and GATA3 in the kidney fibrosis database.

F: Correlation of VIRMA and GATA3 in the kidney fibrosis database. G: Expression levels of GATA3 in the intestinal fibrosis database. H: Expression levels of GATA3 in the liver fibrosis database. I: The mRNA level of VIRMA and GATA3 in the cells. J: The protein expression of α-SMA , Collagen-1, VIRMA and GATA3 in the cells. K: The levels of m6A in total RNA in lung cells. L: The protein expression of α-SMA , Collagen-1,VIRMA and GATA3 in the lung cells. M: The mRNA level of VIRMA and GATA3 in the lung cells. The data are presented as the mean ± SD (n= 3), **P*<0.05 vs. control, ** *P* <0.01 vs. control.

**Supplementary Fig.**4 Overexpression of GATA3 partially reverses the anti-fibrotic effects caused by VIRMA knockdown

A: The protein expression of EMT and ECM markers after VIRMA knockdown or GATA3 overexpression. B: The mRNA level of EMT and ECM markers after VIRMA knockdown or GATA3 overexpression. The data are presented as the mean ± SD (n= 3), **P*<0.05 vs. control, ** *P* <0.01 vs. control, ^#^*P*<0.05 vs VIRMA^KD^+IR , ^##^ *P* <0.05 vs VIRMA^KD^+IR.

**Supplementary Fig.**5 Targeting lactate metabolism reverses radiation-induced profibrotic phenotypes and H3K18la-mediated VIRMA upregulation.

A: Transcriptomic analysis of LDHA and MCT4 mRNA expression in fibrotic versus normal tissues utilizing public databases (GSE110147 and GSE24206). B: Quantification of intracellular ATP levels in control and irradiated RIPF cellular models. C: ECAR in control and irradiated RIPF cellular models. D: Western blotting analysis evaluating the protein expression of key glycolysis-related enzymes and transporters in RIPF cells. E: Real-time monitoring of ECAR in irradiated cells subsequent to co-treatment with exogenous sodium lactate (lac) or dichloroacetate (DCA). F: Western blotting validation of classical epithelial-mesenchymal transition (EMT) marker expression in response to radiation, Lac, or DCA treatment. G: Representative immunoblot showing H3K9la and H3K14la protein levels in the RIPF cellular model. H: ChIP-qPCR assays were performed in A549 and MLE-12 cells using IgG and H3K18la antibodies, followed by qRT-PCR with specific primers targeting the intergenic region. I: Integrative bioinformatics analysis of publicly available H3K18la ChIP-seq datasets (GSE171832 and GSE156675), revealing the specific enrichment profile of H3K18la at the VIRMA promoter region. The data are presented as the mean ± SD (n= 3), **P*<0.05 vs. control, ** *P* <0.01 vs. control.

**Supplementary Fig.6 Knocking down VIRMA partially reverses the fibrogenic effects caused by P300 overexpression**

A: The protein expression EMT and ECM of after VIRMA knockdown or P300 overexpression.

**Supplementary Fig.7 The P300 inhibitor C646 partially reverses RIPF**

A: The protein expression of GATA3 and VIRMA after C646. B: Lung tissue from a P300-inhibited RIPF mice model. C:CT scan of lung tissue from a P300-inhibited RIPF mice model. D: H&E staining of lung tissue from a P300-inhibited RIPF mice model. E: Masson staining of lung tissue in a P300-inhibited RIPF mice model. F:P300 inhibits the in situ expression of α-SMA in lung tissue from RIPF mice. G: P300 inhibits the in situ expression of collagen-1 in lung tissue from RIPF mice.
